# Shared 6mer Peptides of Human and Omicron (21K and 21L) at SARS-CoV-2 Mutation Sites

**DOI:** 10.3390/antib11040068

**Published:** 2022-10-25

**Authors:** Yekbun Adiguzel, Yehuda Shoenfeld

**Affiliations:** 1 Department of Medical Biology, School of Medicine, Atilim University, Ankara 06830, Turkey; 2 Zabludowicz Center for Autoimmune Diseases, Sheba Medical Center, Sackler Faculty of Medicine, Tel Aviv University, Ramat-Gan 52621, Israel

**Keywords:** HLA class I, peptide similarity, SARS-CoV-2, COVID-19, disease susceptibility, autoimmunity

## Abstract

We investigated the short sequences involving Omicron 21K and Omicron 21L variants to reveal any possible molecular mimicry-associated autoimmunity risks and changes in those. We first identified common 6mers of the viral and human protein sequences present for both the mutant (Omicron) and nonmutant (SARS-CoV-2) versions of the same viral sequence and then predicted the binding affinities of those sequences to the HLA supertype representatives. We evaluated change in the potential autoimmunity risk, through comparative assessment of the nonmutant and mutant viral sequences and their similar human peptides with common 6mers and affinities to the same HLA allele. This change is the lost and the new, or de novo, autoimmunity risk, associated with the mutations in the Omicron 21K and Omicron 21L variants. Accordingly, e.g., the affinity of virus-similar sequences of the Ig heavy chain junction regions shifted from the HLA-B*15:01 to the HLA-A*01:01 allele at the mutant sequences. Additionally, peptides of different human proteins sharing 6mers with SARS-CoV-2 proteins at the mutation sites of interest and with affinities to the HLA-B*07:02 allele, such as the respective SARS-CoV-2 sequences, were lost. Among all, any possible molecular mimicry-associated novel risk appeared to be prominent in HLA-A*24:02 and HLA-B*27:05 serotypes upon infection with Omicron 21L. Associated disease, pathway, and tissue expression data supported possible new risks for the HLA-B*27:05 and HLA-A*01:01 serotypes, while the risks for the HLA-B*07:02 serotypes could have been lost or diminished, and those for the HLA-A*03:01 serotypes could have been retained, for the individuals infected with Omicron variants under study. These are likely to affect the complications related to cross-reactions influencing the relevant HLA serotypes upon infection with Omicron 21K and Omicron 21L.

## 1. Introduction

COVID-19 pandemic had a distinct impact on our lives and will possibly affect us more due to its potentially prolonged health outcomes. The disease severity of COVID-19 is immune-related, but the relationship is not straightforward [1,2,3,4,5,6]. The immune responses of people with the disease can lead to autoimmune reactions through the involvement of HLA alleles [7,8,9,10]. Autoimmunity related features are observed in patients with COVID-19 [11,12,13,14,15,16]. Such a probable connection [17,18,19,20,21,22] also led to therapeutic suggestions [23,24,25]. Molecular mimicry is a possible mechanism of autoimmunity induction after infection and even vaccination, where Kanduc and Shoenfeld [26,27,28], and several authors have studied that possibility, along with disease severity upon infection [29,30,31,32,33,34,35,36,37]. A molecular mimicry map of SARS-CoV-2 was also generated [38], and earlier [39], autoimmune-linked MHC alleles (class I and class II) were published [38,40,41,42,43,44]. Emerging variants of concern, specifically the widespread Omicron variant, drew attention [45,46,47] without an Omicron-sourced autoimmunity focus, despite some literature with a broader or a different focus [48,49,50]. Changes in infectivity, prevention by vaccination, and other concerns [51,52,53,54], were of more interest. On a similar basis, there is a need to investigate the possible changes in molecular mimicry-based autoimmunity risk. In accordance, cross-reactivities of Ig antibodies and virus neutralization in mRNA vaccinated people were reported [55], implying the need for more studies. Consequently, the possible molecular mimicry-based autoimmunity risk of the Omicron Nextstrain clades 21K and 21L was investigated here.

We looked for SARS-CoV-2 and Omicron (21K and 21L) peptides at the respective mutation sites and identified those not only similar human proteins but also with affinities to the same HLA alleles as those binding strongly to their similar human peptides. The results were evaluated comparatively. Therefore, the purpose of this work was primarily to identify peptides of human proteins sharing the 6mer with the Omicron 21K and Omicron 21L variants, and with a cross-reaction risk, compared to the respective nonmutant SARS-CoV-2 peptides. This was suggested to pose a risk of molecular mimicry-based autoimmunity, in susceptible individuals, once infected.

## 2. Materials and Methods

This study is conducted with the dataset (Supplementary Data mentioned at the data availability statement, Figure S1 and Document S1–S10) generated to investigate the possible health effects of concern, aroused by human protein-similarities of Omicron (21K and 21L) sequences with mutations. Here, potentially susceptible HLA serotypes were identified through similar human proteins with high affinity peptides. We started this work by generating 6mer sequences of the viral peptides at mutation sites, including both nonmutant (SARS-CoV-2) and mutant (Omicron 21K and Omicron 21L) versions. Then we performed NCBI [56] Blastp [57] searches of these peptides by limiting the search to human. Afterwards, we identified the 6mer-sharing human proteins present for both mutant and nonmutant versions of the viral sequences at the same mutation sites. Human protein-sequences with the aligned 6mers were retrieved from UniProt [58] and NCBI [56] in fasta format. The 8mers of these identified similar virus and human peptide pairs were predicted for their HLA affinities [59,60,61,62,63,64,65]. Strong-binder (SB < 0.5% rank) and weak-binder (0.5% < WB < 2% rank) results of NetMHCcons, and epitope (E) results of NetCTLpan were high affinity peptides, also referred to as peptides with affinity. Peptide pairs with high affinities to the same HLA allele were deemed as autoimmunity risk-bearing peptides in the susceptible individuals with those serotypes, upon infection with the virus of concern. Viral/human peptide pairs with high affinities were evaluated to infer changes in the autoimmunity risks for the susceptible serotypes upon infection, through lost or gained affinities of the viral/human peptide pairs. We also evaluated changes in the alleles with high affinities to the viral/human peptide pairs.

Features of the proteins were outlined through the information retrieved from NCBI Entrez [66], UniProtKB/Swiss Prot [58], MalaCards [67,68], SuperPathways [69], and ProteinDB [70,71], collected from the dedicated websites of the GeneCards [72]. Network images were prepared with that information and the data, using Cytoscape [73] version 3.8.2, running with Java 11.0.6. Phylogeny images were generated at covariants.org, on 26 May 2022. Further details of the methodology are provided in the Appendix A, within Appendix A.1.

## 3. Results and Discussion

**Figure 1:**
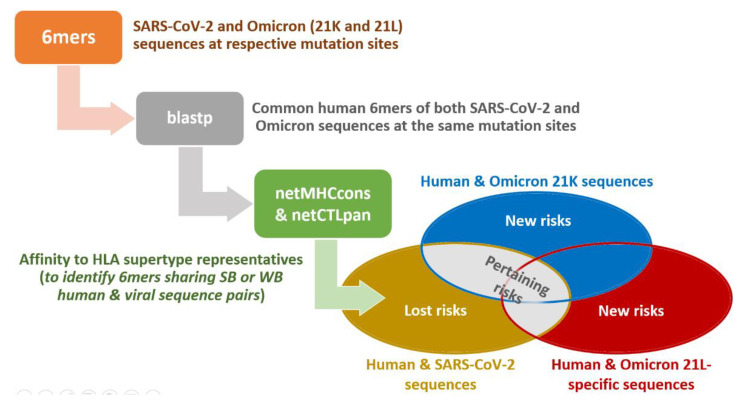
The outline of the methodology. We first prepared 6mer peptides at the Omicron 21K and Omicron 21L mutation sites, along with the SARS-CoV-2 peptides at the respective mutation sites, and then performed blastp searches to find human proteins containing those 6mers. Human peptides sharing 6mers with SARS-CoV-2 and Omicron sequences at the same mutation sites were selected. Selected SARS-CoV-2/human and Omicron/human peptide pairs were predicted for their binding affinities to the HLA supertype representatives, to identify strong-binder (SB) and weak-binder (WB) peptides. Those peptide pairs with such high affinities to the same alleles were evaluated as the lost cross-reaction risks in the susceptible individuals, upon infection, if they were exclusively SARS-CoV-2/human peptide pairs. Such peptide pairs were evaluated as the new, or de novo, risks, if they were exclusively Omicron/human peptide pairs. They were evaluated as pertaining risks if they were both SARS-CoV-2/human and Omicron/human peptide pairs of sequences at the same mutation sites. Omicron/human peptide pairs included Omicron sequences that were separated into Omicron 21K sequences and Omicron 21L-specific sequences, where the Omicron 21K sequences also involved sequences at mutation sites common to both Omicron 21K and Omicron 21L.

The methodology of this study is summarized in Figure 1. Targeting only the human/Omicron peptide pairs with affinities to the same HLA allele, namely, identifying the human peptides that can cross-react with peptides of the Omicron 21K and Omicron 21L, would have been a classical approach. Differently, evaluation of its results compared to the results of human peptides that can cross-react with SARS-CoV-2 peptides at the mutation sites is a novel approach. This approach enabled us to obtain the essence of Omicron 21K- and Omicron 21L-sourced changes. With this, one may recognize how molecular mimicry-based autoimmunity risk could shift from one susceptible group to the other.

### 3.1. Identified Human Proteins and Peptides

Information on the general features of the identified human proteins is provided in alphabetical order in the Appendix A, within Appendix A.2. The results of our current Blastp search extended the list of sequences obtained through our preliminary work [37] (Table 1). That preliminary work used more restricted parameters, and did not focus on 6mers, as in this work.

Table 2 (row 1 to 11) displays the first part of the current results, belonging to SARS-CoV-2 peptides containing the Omicron 21K-specific, and Omicron 21K- and Omicron 21L-common, mutation sites. Human peptides sharing 6mers with them and having affinity to the same HLA allele are presented along. Table 2 (row 12 to 21) also displays the results for the corresponding mutant sequences, along with their similar human peptide sequences. The two parts of the table, i.e., results until row 12 and the results afterwards, exclude each other. Accordingly, potential cross-reactive peptides until row 10 represent the diminished risks due to mutations and those after row 11, except those at rows 18 and 19, represent the novel risks in the susceptible individuals, upon getting infected. Viral peptides displayed at rows 10 and 11, and at rows 18 and 19 are nonmutant and mutant versions of the same mutation site, respectively. Accordingly, human peptides mimicking those represent a retaining risk in case of the HLA-B*15:01 serotypes.

Table 3 (row 1 to 16) displays the results for SARS-CoV-2 peptides at the sites mutated specifically in Omicron 21L, and the human peptides both shared 6mers with them and had affinity to the same HLA allele. Table 3 (row 17 to 29) also displays results for the viral peptides with Omicron 21L-specific mutations, and human peptides both sharing 6mers with them and having an affinity to the same HLA allele. Potential cross-reactive peptides with the sequences displayed until row 17, except the results in rows 3–6, represent the diminished risks with mutations and the remaining peptides, except that displayed at row 20, represent novel risks. However, some data in Table 3 can be interpreted as de novo risks. For example, human peptides in rows 12 and 29, which are at two separate parts of the table, both shared 6 aa with the corresponding viral peptides at positions 367–374 of the spike protein, had affinity to the same allele, and belonged to the same type of protein. In another case, human peptides in rows 6 and 20, also shared 6 aa with the corresponding viral peptides and had affinity to the same allele but did not belong to the same type of protein. Additionally, viral peptides at row 3, and at row 20 are nonmutant and mutant versions of the same mutation site, respectively. Accordingly, human peptides mimicking those represent a retaining risk in case of the HLA-A*03:01 serotypes.

The numerical results of the data in Table 2 and Table 3 are presented in Table 4. WB/SB/E peptides of human proteins sharing 6mers with SARS-CoV-2 sequences at Omicron 21L-specific mutation sites in the Orf1ab protein region decreased the most (from 7 to 3). Deletions were more common than insertions among the mutations of interest in Omicron. Accordingly, a decrease in the number of sequences that can cross-react with human proteins was expected. However, this was not the case (Table 4).

**Figure 2:**
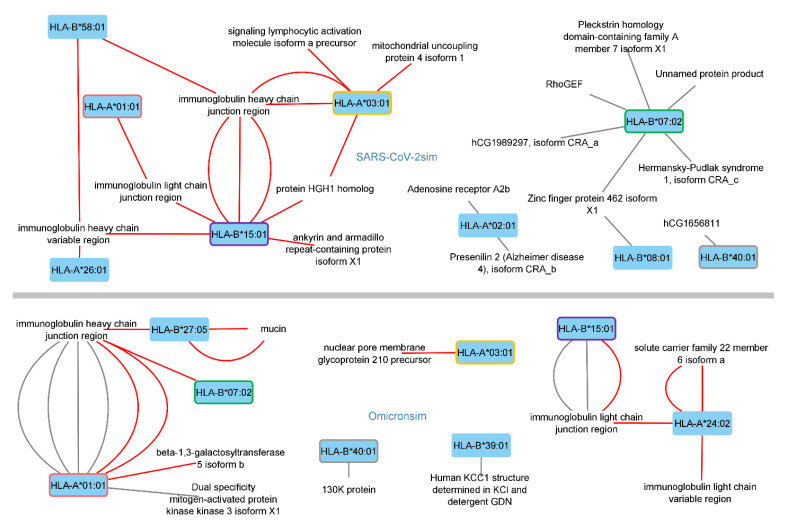
Networks of human proteins with virus-similar peptides at the mutation sites of interest and the HLA alleles, to which they had affinity. Alleles are connected to the proteins through the peptide of that protein mimicking the viral peptide and with strong affinity to the connected allele, such as the mimicked viral peptide. The top part displays those of human proteins with SARS-CoV-2 similar (SARS-CoV-2sim) peptides. The bottom part displays those of human proteins with Omicron similar (Omicronsim) peptides. Alleles at both parts are encircled with the same color indicator of that allele. Other alleles are not encircled. Red edges (i.e., connections) belong to the human proteins sharing 6mers with SARS-CoV-2 sequences at Omicron 21L-specific mutation sites (on **top**), and to the human proteins sharing 6mers with sequences containing Omicron 21L-specific mutations (at the **bottom**). Affinity refers to weak-binder/strong-binder/epitope (WB/SB/E). (Ring Finger Protein 10 was identified as an unnamed protein product in the Blastp alignment document).

Figure 2 presents the numbers of SARS-CoV-2 and Omicron (21K and 21L) similar human peptides (SARS-CoV-2sim and Omicronsim) with predicted-affinities to the given HLA alleles of interest. Figure 2 indicates a possible shift of the alleles, which could put the individuals at risk. One can roughly view the SARS-CoV-2sim data in Figure 2 as the lost risks due to the mutations and the Omicronsim data as the new or de novo risks, with exceptions of those termed as retaining risks, mentioned above. Six of the Omicron-similar peptides with HLA-A*01:01 affinities were immunoglobulin (Ig) heavy chain junction regions (Table 2, rows 12−15, and Table 3, rows 17−18). Ig light chain or heavy chain parts made-up 5 of the 7 SARS-CoV-2 similar peptides with affinities to the HLA-B*15:01 allele (Table 3, rows 10–16). Such peptides can lead to the generation of anti-idiotypic autoantibodies. These results were interpreted as a shift of the Ig heavy chain junction-sourced peptide affinities from the HLA-B*15:01 allele to the HLA-A*01:01 allele. This interpretation was based additionally on the overall comparison of the data in Table 2 and Table 3. This shift is also illustrated in Figure 2. Along with this shift, there was also a decrease in the potential risk of anti-idiotypic antibodies generated against the Ig heavy chain variable regions.

Differences in the peptides with HLA-A*24:02 affinities were due to Omicron 21L-specific mutations, as they are observed exclusively in the second part of Table 3, which belongs to the respective results of the 21L-specific mutations. These mutations led to new, similar human peptides with WB/SB affinity. Differences in the peptides with HLA-B*07:02 affinities were due to mutations other than the Omicron 21L-specific ones, which led to the loss of similar human peptides with affinities to that allele (rows 3−7, Table 2). Additionally, in that case, peptides sourced by different types of proteins shared the same 6mer of the SARS-CoV-2 peptide. This is well illustrated in Figure 2 as well. Finally, any possible molecular mimicry-associated novel risk seemed to be the most prominent in Omicron 21L-infected HLA-A*24:02 and HLA-B*27:05 serotypes (Figure 2), based on the present data.

### 3.2. Disorders, Pathways, and Expression Sites

**Figure 3:**
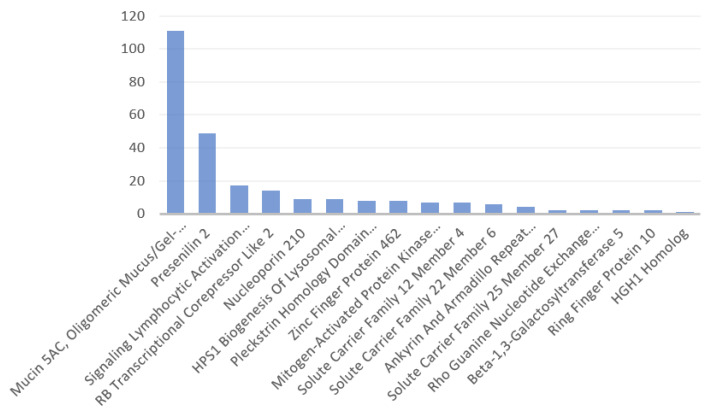
Number of disorders associated with the identified human proteins in descending order. Mucin is outstanding with the highest number of associated disorders, compared to the other proteins with the respective data (Appendix A, Table A1).

Figure 3 displays the number of disorders per protein identified here, excluding those without data at Genecards. Mucin, viral-peptide mimicking part of which was identified to be involving in a novel risk for the HLA-B*27:05 serotypes, was associated with the highest number of disorders, and the next protein was presenilin 2, which was suggested to be rather in a lost risk due to containing a SARS-CoV-2 mimicking peptide with affinity to the HLA-A*02:01 allele. The identified proteins did not share the associated disorders. Table A1 at Appendix A presents the list of disorders associated with the identified proteins.

**Figure 4:**
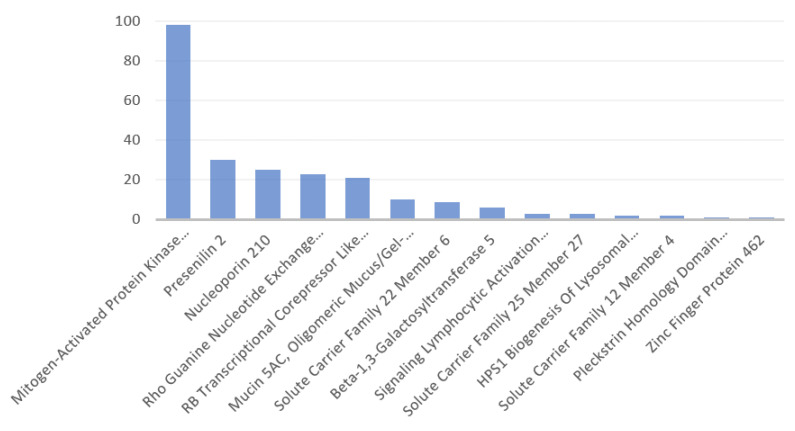
Number of associated superpathways of the identified human proteins in descending order. Mitogen activated protein kinase kinase 3 is outstanding with the highest number of associated superpathways, compared to the other proteins with the respective data (Appendix A, Table A2).

Figure 4 presents the number of the associated superpathways with the identified proteins. The majority of involved superpathways were associated with only one identified protein. Each identified protein associated with several numbers of different superpathways, as revealed by the excess of associated superpathways compared to the present number of identified proteins. In four cases, more than 2 proteins associated with a superpathway, as follows:

Mucin 5AC (MUC5AC), mitogen activated protein kinase kinase 3 (MAP2K3), and nucleoporin 210 (NUP210) share the innate immune system.

Presenilin 2 (PSEN2), MAP2K3, and Rho Guanine Exchange Factor 4 (ARHGEF4) share ERK signaling.

NUP210, beta-1,3-galactosyltransferase 5, and solute carrier family 25 member, 27 share metabolism.

MUC5AC, NUP210, MAP2K3, and PSEN2 share the superpathway disease.

The number of superpathways shared by 2 proteins was 22. MAP2K3 was the most frequently (i.e., 13) observed protein in those superpathways shared by 2 proteins. Among those superpathways, MAPK-Erk was shared by the proteins MAP2K3 and RB transcriptional corepressor like 2 (130K protein). Although viral-peptide mimicking part of the 130K protein was identified here to be involving in a retained risk for the HLA-B*40:01 serotypes, that of the MAP2K3 protein was found to carry a potential of leading to a new autoimmune reaction risk in the HLA-A*01:01 serotypes. The risk would have been more if the respective peptide of 130K protein and the Omicron peptide it mimicked both had affinities to the HLA-A*01:01 allele.

PSEN2 and NUP210 were the two succeeding proteins associated with the highest number of superpathways (Figure 4), ARHGEF4 had the second-highest rate of presence (i.e., 7) in the superpathways shared by 2 proteins. ARHGEF4 and MAP2K3 comprised the two proteins in 5 superpathways shared by 2 proteins, but ARHGEF4 viral-peptide mimicking part of it was identified to be involving in a lost risk for the HLA-B*07:02 serotypes. Table A2 at Appendix A presents the list of superpathways associated with the identified proteins.

**Figure 5:**
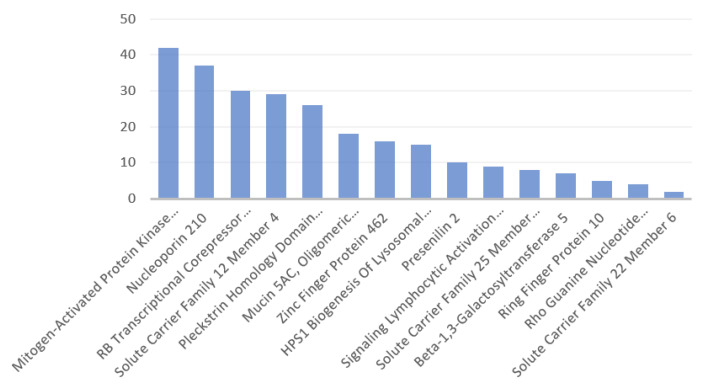
Number of tissues expressing the identified human proteins in descending order. The highest number of tissues express mitogen activated protein kinase kinase 3, compared to the expression of the other proteins with the respective data (Appendix A, Table A3).

**Figure 6:**
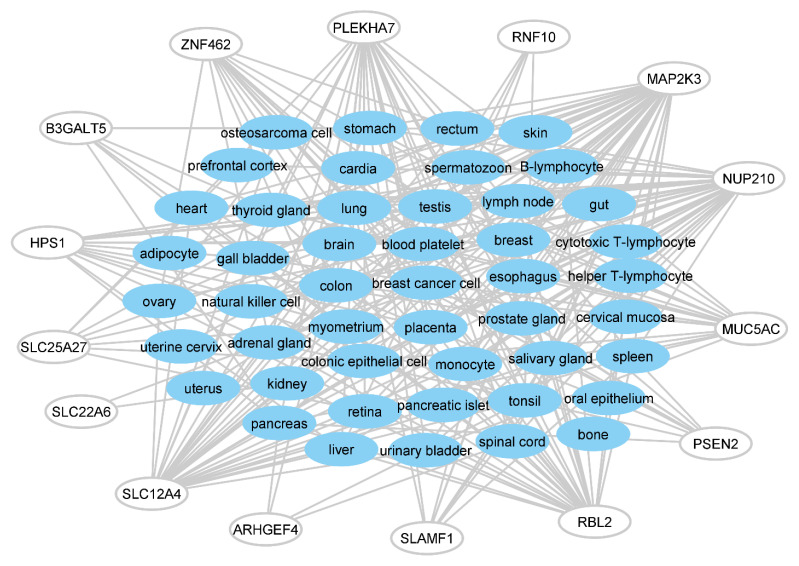
Networks of the identified human proteins with the tissues expressing them. Proteins with the abbreviations in the figure: Beta-1,3-Galactosyltransferase 5 (B3GALT5), HPS1 Biogenesis Of Lysosomal Organelles Complex 3 Subunit 1 (HPS1), Mitogen-Activated Protein Kinase Kinase 3 (MAP2K3), Mucin 5AC, Oligomeric Mucus/Gel-Forming (MUC5AC), Nucleoporin 210 (NUP210), Pleckstrin Homology Domain Containing A7 (PLEKHA7), Presenilin 2 (PSEN2), RB Transcriptional Corepressor Like 2 (RBL), Rho Guanine Nucleotide Exchange Factor 4 (ARHGEF4), Ring Finger Protein 10 (RNF10), Signaling Lymphocytic Activation Molecule Family Member 1 (SLAMF1), Solute Carrier Family 12 Member 4 (SLC12A4), Solute Carrier Family 22 Member 6 (SLC22A6), Solute Carrier Family 25 Member 27 (SLC25A27), Zinc Finger Protein 462 (ZNF462). (Identified proteins without the relevant data in the database are not represented in the figures. See Appendix A.2).

Table A3 at Appendix A presents the list of tissues expressing the identified proteins, along with the expression levels. The total number of tissues expressing MAP2K3 was the highest (i.e., 42, Figure 5). It is expressed in almost all tissues displayed in Figure 6, except the prefrontal cortex, osteosarcoma cells, spermatozoon, cervical mucosa, and bone. Therefore, if infected, cross-reaction of the Omicron 21K-mimicking peptide of MAP2K3 in the HLA-A*01:01 serotypes could involve several tissues and organs. Among those, adipocyte, oral epithelium, skin, uterine cervix, and uterus are expressing only MAP2K3, while cervical mucosa is expressing only MUC5AC, and cardia is expressing MAP2K3 and MUC5AC, among the identified proteins (Appendix A, Table A3).

**Figure 7:**
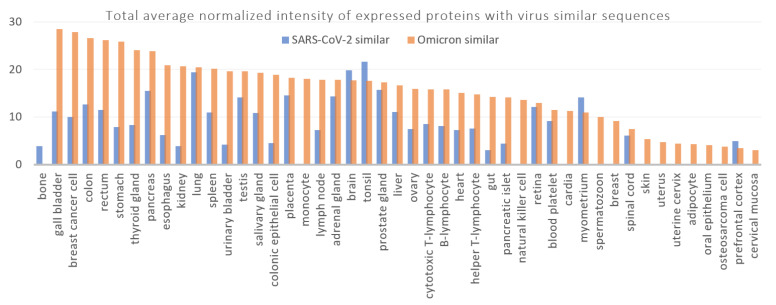
Total average normalized intensities of the identified human protein expression levels in the given tissues, in descending order of the total average normalized intensities of the expressed proteins with Omicron-similar peptides.

If we look at the total average normalized intensities of the expression levels of the identified human proteins, gall bladder has the highest expressions of the identified proteins with Omicron-similar sequences, followed by breast cancer cell, colon, rectum, stomach, thyroid glands, and pancreas (Figure 7). High expression of the given proteins in those tissues could categorize them as potentially the most vulnerable targets if an autoimmune reaction is developed against those proteins, in the susceptible individuals who are infected with the Omicron variant. The total average normalized intensity of the expressed proteins exclusively with Omicron similar sequences was approximately two times greater than that of proteins exclusively with SARS-CoV-2 similar sequences. It should be reminded that any suggested biological relevance is limited to the possible effects of the mutation sites of the Omicron 21K and Omicron 21L variants.

**Figure 8:**
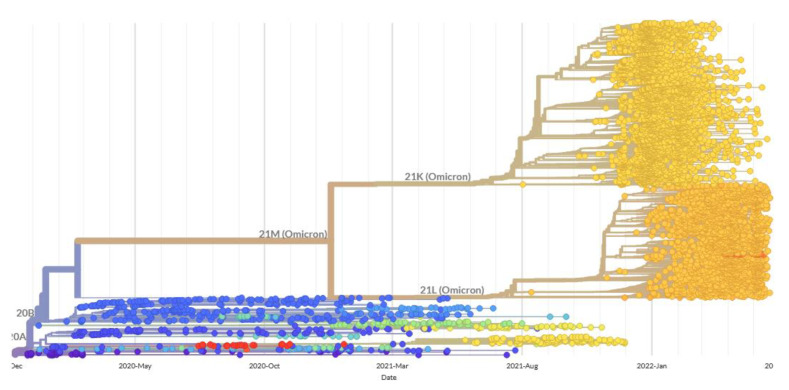
Phylogenetic analysis of the SARS-CoV-2 clusters, including 21K (Omicron) and 21L (Omicron). Image generated at covariants.org, on 26 May 2022.

**Figure 9:**
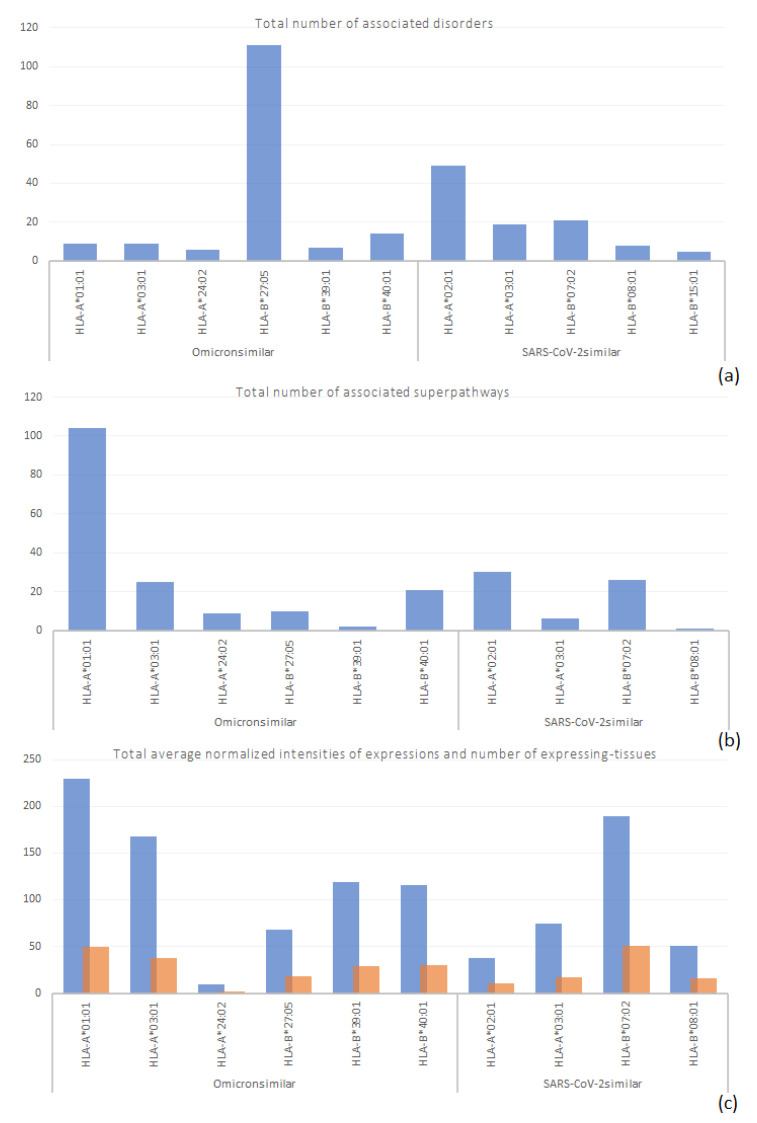
Total number of associated disorders, pathways, and expression levels (blue), along with the number of expressing tissues (orange), of the identified human proteins, with respect to the high affinity alleles of the viral peptide mimicking parts of those proteins. The total number of associated disorders reveals a possible risk in case of the HLA-B*27:05 serotypes (**a**). The total number of associated superpathways reveals a possible risk in case of the HLA-A*01:01 serotypes upon getting infected with the Omicron variant (**b**), which is supported by the total number of expression levels in (**c**). Collective data of approximately 7–8 identified proteins with the relevant information were used to plot the graphs.

The efforts in this study were to specify the serotypes at risk and to explain a possible mechanism of the shift in disease severity among certain serotypes, due to mutations in Omicron 21K and Omicron 21L. However, other than individual susceptibilities, there is also the possibility of becoming infected with a different variant, which is immense even among the Omicron 21K and Omicron 21L, in addition to the other variants (Figure 8). Studies such as this one aim to provide a generalized understanding. In line with this aim, Section 3.2 of this study revealed that associated disorders and superpathways of the identified human proteins with Omicron mimicking peptides revealed possible new risk for the HLA-B*27:05 and HLA-A*01:01 serotypes, respectively (Figure 9). The latter is supported by the tissue-expression data (Figure 9). On the other hand, risk for the HLA-B*07:02 serotypes could have been diminished (Figure 9) and that for the HLA-A*03:01 serotypes could have been retained. Finally, high affinity peptides of the human proteins identified here are not yet observed in vivo or in vitro as autoantigens. However, that is likely because of lacking experimental studies aiming to detect those autoantibodies. In support of the possibility of demonstrating the presence of autoantibodies, cross-reaction of peptide PFERD at 463–467 positions of the spike protein receptor binding domain (S1-RBD) of SARS-CoV-2 with the human cell receptor angiotensin-converting enzyme 2 was delicately identified by Lai et al. [74], through several experimental steps, which are the demonstration of cross-reaction in patients’ sera (1), demonstration of cross-reaction in sera of mice immunized with recombinant S1-RBD (2), identification of monoclonal antibodies (mAbs) that could cross-react (3), and finding the cross-reactive antigenic peptide that could bind strongly to the autoreactive mAb (4).

This work focused on human molecular mimicry-based autoimmunity risk changes in different HLA serotypes, by considering only the sequences at mutation sites of the nonmutant SARS-CoV-2 and mutant Omicron (21K and 21L) sequences into account. Such changes can influence viral evolution, yet the involvement of the HLA interactions with the spike protein [75] could be the major driving factor, along with its effects on transmissibility [76], and with the contribution of vaccines to this phenomenon. Accordingly, amendments of our work can involve conducting a study with a broader perspective, by including considerations on different aspects of HLA interactions, in addition to evaluating the missed and eliminated data due to selected search parameters/criteria, including a possible future work on the shared 5mers. Studying mutations of the other variants, plus their recombination [77], and predicting affinities to the other alleles, including especially the class II alleles, are of importance.

## 4. Conclusions

A change in the potential autoimmunity risk is any loss in the potential autoimmunity risk due to mutations, with any new or de novo risks associated with those mutation sites. We identified the lost and gained similarities with the human peptides, as a risk of triggering autoimmunity due to cross-reactivity in susceptible individuals infected with Omicron 21K and Omicron 21L. Among all, any possible molecular mimicry-associated novel risk seemed to be the most prominent in HLA-B*27:05 and maybe also in HLA-A*24:02 serotypes who are infected with Omicron 21L. Results further supported possible new risk for the HLA-B*27:05 and HLA-A*01:01 serotypes, while the risk for the HLA-B*07:02 serotypes could have been lost or diminished, and that for the HLA-A*03:01 serotypes could have been retained, for the individuals infected with Omicron variants under study. While the results require clinical validation, they may provide an explanation for such a possible autoimmunity-related new or lost symptoms in Omicron 21K- or Omicron 21L-infected susceptible individuals.

## Figures and Tables

**Table 1:** Omicron 21K and Omicron 21L spike protein sequences with similar sequences in the human proteome and with affinities to the same HLA alleles as those of the human sequences. Omicron/human common residues are written in bold, and residues with mutations are additionally underlined. Only the highlighted results at the fifth results-line are specific to Omicron 21L [37]. Adapted with permission from Kenes.

Omicron Peptide	Human Peptide	Human Protein Name	Human Protein ID	Allele	Tool for Prediction
N**L**A**PFFTF**	L**L**S**PFFTF**	Ig kappa chain variable region	ABA71433.1	HLA-A*24:02	NetCTLpan
N**L**A**PFFTF**	L**L**S**PFFTF**	Ig kappa chain variable region	ABA71433.2	HLA-B*15:01	NetCTLpan
N**L**A**PFFT**F	Y**L**S**PFFT**Y	hCG2003071	EAW54993.1	HLA-B*15:01	NetCTLpan
YN**L**A**PFFT**F	YY**L**S**PFFT**Y	hCG2003071	EAW54993.1	HLA-A*24:02	NetCTLpan/NetMHCcons
N**FAPF**-**FAF**	**FAPF**L**FAF**	hCG2023603	EAW76558.1	HLA-A*24:02	NetCTLpan
**FPLRS**Y**S**F	**FPLRS**F**S**Y	Ig heavy chain junction region	MOM40044.1	HLA-B*07:02	NetCTLpan

**Table 2:** Virus and human peptides sharing 6mers at the mutation sites of interest and having affinity to the same HLA. The first 11 data rows are the respective SARS-CoV-2 and human peptides. The corresponding SARS-CoV-2 peptides are those at the Omicron 21K-specific mutation sites, and at the mutation sites common to Omicron 21K and Omicron 21L (i.e., 21K + 21K/21L). The rows from 12 to the end display human peptides sharing 6mers with the respective Omicron (21K + 21K/21L) sequences. Empty cells indicate that the data is the same as the data in the last filled cell above that row. Shared residues in the human peptides are written in bold.

	**HLA ^1^**	**SARS-CoV-2 Peptide**	**Prediction ^2^**	**Human Peptide**	**Prediction ^2^**	**Human Protein Name**	**Human Protein ID ^3^**
1	A2	TLACFVLA	WB	**TLACFV**AI	WB	Presenilin 2 (Alzheimer disease 4), isoform CRA_b	EAW69797.1
2				F**LACFVL**V	SB	Adenosine receptor A2b	NP_000667.1
3	B7	SPRRARSV	SB/E	**SPRRAR**II	SB	Zinc finger protein 462 isoform X1	XP_006717272.1
4				**SPRRAR**GH	WB	Pleckstrin homology domain-containing family A member 7 isoform X1	XP_047282382.1
5				G**PRRARS**A	WB	Unnamed protein product ^4^	BAG54301.1
6				P**PRRARS**V	WB	RhoGEF	AAF79955.1
7				S**PRRARS**S	WB	Hermansky-Pudlak syndrome 1, isoform CRA_c	EAW49879.1
8	B7	PPTSFGPL	WB	V**PTSFGPL**	SB	hCG1989297, isoform CRA_a	EAW55845.1
9	B8	SPRRARSV	WB/E	**SPRRAR**II	WB	Zinc finger protein 462 isoform X1	XP_006717272.1
10	B44	SEETGTLI	WB/E	P**ETGTLI**V	WB	hCG1656811	EAW75628.1
11	B44	EETGTLIV	WB				
	**HLA ^1^**	**Omicron Peptide**	**Prediction ^2^**	**Human Peptide**	**Prediction ^2^**	**Protein Name**	**Human Protein ID ^3^**
12	A1	SGNYNYLY	WB/E	GL**SGNYNY**	**WB**	Immunoglobulin heavy chain junction region	MOL95178.1
13				YG**SGNYNY**	**WB**	Immunoglobulin heavy chain junction region	MOL73314.1
14				G**SGNYNY**Y	**SB**	Immunoglobulin heavy chain junction region	MBB1884951.1
15				**SGNYNY**FY	**WB**	Immunoglobulin heavy chain junction region	MOL21912.1
16	A1	LTSFGPLV	WB	I**LTSFGP**Y	**WB**	Dual specificity mitogen-activated protein kinase kinase 3 isoform X1	XP_016880346.2
17	B39	MHSALRLV	WB	DR**HSALRL**	**WB**	Human KCC1 structure determined in KCl and detergent GDN	6KKR_A
18	B44	SEEIGTLI	WB/E	AE**EEIGTL**	**SB**	130K protein ^5^	CAA53661.1
19	B44	EEIGTLIV	WB/E				
20	B62	FLARGVVF	SB/E	AG**ARGVVF**	**WB**	Immunoglobulin light chain junction region	MCC96497.1
21				SG**ARGVVF**	**WB**	Immunoglobulin light chain junction region	MCB29717.1

^1^ A1: HLA-A*01:01, A2: HLA-A*02:01, A3: HLA-A*03:01, A24: HLA-A*24:02, A26: HLA-A*26:01, B7: HLA-B*07:02, B8: HLA-B*08:01, B62: HLA-B*15:01, B27: HLA-B*27:05, B39: HLA-B*39:01, B44: HLA-B*40:01, B58: HLA-B*58:01. ^2^ Weak-binder (WB) and strong-binder (SB) predictions by NetMHCcons, and epitope (E) predictions by NetCTLpan. ^3^ Only one protein ID, commonly the first one that appeared in the alignments, is provided. ^4^ Ring Finger Protein 10 was identified as an unnamed protein product in the Blastp alignment document. ^5^ RB Transcriptional Corepressor Like 2 was identified as 130K protein in the Blastp alignment document.

**Table 3:** Viral (SARS-CoV-2 and Omicron 21L) and human peptides that share 6mers at the Omicron 21L-specific mutation sites and have affinity to the same HLA. The first 16 data rows are the respective SARS-CoV-2 and human peptides. The rest are the Omicron 21L and human peptides. (Table format features are the same as the relevant explanation at the caption of Table 2).

	**HLA ^1^**	**SARS-CoV-2 Peptide**	**Prediction ^2^**	**Human Peptide**	**Prediction ^2^**	**Human Protein Name**	**Human Protein ID ^3^**
1	A1	RTQLPPAY	WB/E	SI**QLPPAY**	E	Immunoglobulin light chain junction region	MCD11024.1
2	A3	FLGVYYHK	WB/E	GT**FLGVYY**	WB	Immunoglobulin heavy chain junction region	MBN4196023.1
3	A3	VLLPLTQY	WB	R**LLPLTQY**	WB	Protein HGH1 homolog	NP_057542.2
4				R**LLPLTQ**R	WB	Mitochondrial uncoupling protein 4 isoform 1	NP_004268.3
5				**VLLPLT**YY	WB	Immunoglobulin heavy chain junction region	MBN4485217.1
6				K**VLLPLT**Y	WB	Signaling lymphocytic activation molecule isoform a precursor	NP_001317683.1
7	A26	NSASFSTF	E	SV**ASFSTF**	SB	Immunoglobulin heavy chain variable region, partial	UNJ97266.1
8	B58	RTQLPPAY	E	I**QLPPAY**W	SB	Immunoglobulin heavy chain junction region	MOQ03906.1
9	B58	NSASFSTF	WB/E	**ASFSTF**TI	WB	Immunoglobulin heavy chain variable region, partial	UNJ97266.1
10	B62	RTQLPPAY	WB	Y**QLPPAY**Y	WB	Immunoglobulin heavy chain junction region	MCG70934.1
11				C**QLPPAY**Y	WB	Ankyrin and armadillo repeat-containing protein isoform X1	XP_011508975.1
12	B62	VLYNSASF	SB/E	**YNSASF**TF	WB	Immunoglobulin light chain junction region	MBB1719028.1
13	B62	NSASFSTF	WB/E	SV**ASFSTF**	SB	Immunoglobulin heavy chain variable region, partial	UNJ97266.1
14	B62	KGAGGHSY	WB	Q**GAGGHSY**	WB	Immunoglobulin heavy chain junction region	MBN4552893.1
15	B62	VLLPLTQY	WB	**VLLPLT**YY	WB	Immunoglobulin heavy chain junction region	MBN4485217.1
16				R**LLPLTQY**	WB	Protein HGH1 homolog	NP_057542.2
	**HLA ^1^**	**Omicron 21L Peptide**	**Prediction ^2^**	**Human Peptide**	**Prediction ^2^**	**Human Protein Name**	**Human Protein ID ^3^**
17	A1	FLDVYYHK	WB	**FLDVYY**GM	WB	Immunoglobulin heavy chain junction region	MBN4448374.1
18				**FLDVYY**YY	SB	Immunoglobulin heavy chain junction region	MCG72449.1
19				**FLDVYY**NL	WB	Beta-1,3-galactosyltransferase 5 isoform b	NP_149362.2
20	A3	VLLPFTQY	WB/E	K**VLLPFT**R	WB	Nuclear pore membrane glycoprotein 210 precursor	NP_079199.2
21	A24	DYSVLYNF	WB/E	SQ**SVLYNF**	WB	Immunoglobulin light chain variable region, partial	AHZ09416.1
22	A24	LYNFAPFF	SB/E	**YNFAPF**TF	WB	Immunoglobulin light chain junction region	MCE34472.1
23	A24	NFAPFFAF	SB/E	VS**APFFAF**	WB	Solute carrier family 22 member 6 isoform a	NP_004781.2
24				S**APFFAF**F	WB	Solute carrier family 22 member 6 isoform a	NP_004781.2
25	B7	FPLRSYGF	WB/E	S**PLRSYG**M	WB	Immunoglobulin heavy chain junction region	MBB2034746.1
26	B27	HRYGADLK	SB/E	**HRYGAD**YY	WB	Immunoglobulin heavy chain junction region	MBB1980753.1
27	B27	ARLCAKHY	WB/E	LR**ARLCAK**	SB	Mucin, partial	AAC15950.1
28				**ARLCAK**GV	WB	Mucin, partial	AAC15950.1
29	B62	VLYNFAPF	SB/E	**YNFAPF**TF	WB	Immunoglobulin light chain junction region	MCE34472.1

^1^ A1: HLA-A*01:01, A2: HLA-A*02:01, A3: HLA-A*03:01, A24: HLA-A*24:02, A26: HLA-A*26:01, B7: HLA-B*07:02, B8: HLA-B*08:01, B62: HLA-B*15:01, B27: HLA-B*27:05, B39: HLA-B*39:01, B44: HLA-B*40:01, B58: HLA-B*58:01. ^2^ Weak-binder (WB) and strong-binder (SB) predictions by NetMHCcons, and epitope (E) predictions by NetCTLpan. ^3^ Only one protein ID, commonly the first one that appeared in the alignments, is provided. e.g., FLDVYYGM was also a part of immunoglobulin heavy chain alpha VDJ region, partial (ID: AAD15877.1).

**Table 4:** The number of WB/SB/E predictions of human proteins (i.e., similar) sharing 6mer with SARS-CoV-2 or Omicron (21K and 21L) at mutation sites and having affinity to the same HLA allele. The first 4 data-columns exclude the relevant data of the Omicron 21L-specific mutation sites. The last 4 columns are the relevant data of the Omicron 21L-specific mutation sites.

	SARS-CoV-2 Similar Sequences at Omicron (21K + 21K/21L) Sites		Similar of Omicron (21K + 21K/21L) Sequences with Mutations		SARS-CoV-2 Similar Sequences at Omicron 21L Sites		Similar of Sequences with Omicron 21L-Specific Mutations	
	WB	SB	WB	SB	WB	SB	WB	SB
Orf1ab	0	1	3	0	7 ^1^	0	3	1
Spike	5	1	3	1	6	3	8	1
Orf9b	0	0	1	0	0	0	0	0
Envelope	1	0	0	1	0	0	0	0
Matrix	1	1	0	0	0	0	0	0
Total	7	3	7	2	13	3	11	2

One different sequence was predicted as E by NetCTLpan. It was included in the WB column. The other respective predictions of NetCTLpan were common to NetMHCcons.

## Data Availability

Data is contained within the article or supplementary materials.

## Supplementary Materials

The following supporting information can be downloaded at: https://www.mdpi.com/article/10.3390/antib11040068/s1, and available as Mendeley Data [78]. Figure S1: Relevant mutations displayed at covariants.org, on 26 May 2022. Document S1: Blastp search input sequences involving mutations specific for Omicron 21K and mutations common to both Omicron 21K and Omicron 21L, along with the respective SARS-CoV-2 sequences. Document S2: Blastp search input sequences involving mutations specific for Omicron 21L, along with the respective SARS-CoV-2 sequences. Document S3: Alignment output of Blastp search with the input sequences involving mutations specific for Omicron 21K and mutations common to both Omicron 21K and Omicron 21L. Document S4: Alignment output of Blastp search with the input sequences involving mutations specific for Omicron 21L. Document S5: NetCTLpan HLA prediction-results of 364 human sequences sharing 6mers with sequences involving Omicron 21K-specific mutations and with sequences involving mutations common to both Omicron 21K and Omicron 21L (*Last 1-peptide prediction was performed after the initial 363-peptides prediction*). Document S6: NetMHCcons HLA prediction-results of 364 human sequences sharing 6mers with sequences involving Omicron 21K-specific mutations and with sequences involving mutations common to both Omicron 21K and Omicron 21L (*Last 1-peptide prediction was performed after the initial 363-peptides prediction*). Document S7: NetCTLpan HLA prediction-results of 242 human sequences sharing 6mers with sequences involving Omicron 21L-specific mutations (*Last 9-peptide prediction was performed after the 233-peptides prediction results*). Document S8: NetMHCcons HLA prediction-results of 242 human sequences sharing 6mers with sequences involving Omicron 21L-specific mutations (*Last 9-peptide prediction was performed after the 233-peptides prediction results*). Document S9: Source organisms of the initially predicted 363 sequences in documents S5 and S6. (*Includes deleted results after predictions indicated with a stroke-through the content at the respective lines*). Document S10: Source organisms of the initially predicted 233 sequences in documents S7 and S8. (*Includes corrected names after predictions, at ID#217–219*). Document S11: NetCTLpan HLA prediction-results of 333 sequences involving Omicron 21K-specific mutations and sequences involving mutations common to both Omicron 21K and Omicron 21L. Document S12: NetMHCcons HLA prediction-results of 333 sequences involving Omicron 21K-specific mutations and sequences involving mutations common to both Omicron 21K and Omicron 21L. Document S13: NetCTLpan HLA prediction-results of 206 sequences involving Omicron 21L-specific mutations. Document S14: NetMHCcons HLA prediction-results of 206 sequences involving Omicron 21L-specific mutations.

## Appendix A

### Appendix A.1. Extended Materials and Methods

#### Appendix A.1.1. The Blastp Searches

National Center for Biotechnology (NCBI) was the main source for sequence information of SARS-CoV-2 reference proteins [56]. These proteins were open reading frame (Orf)1ab (ID: YP_009724389.1) containing Orf1a, nonstructural protein (Nsp)3, Nsp4, Nsp5, Nsp6, and Orf1b; spike glycoprotein (S, ID: YP_009724390.1); Orf9b (ID: P0DTD2); envelope protein (E, ID: YP_009724392.1); nucleocapsid protein (N, ID: YP_009724397.2); and matrix protein (M, ID: YP_009724393.1). Mutations of the Omicron Nextstrain clades 21K and 21L were obtained from covariants.org, on 26 May 2022. Six amino acid (aa)-long sequences (6mers) at the mutation sites of the viral proteins were generated by a sliding-window approach, namely by including all respective sequences with possible different positions of a mutation, starting from the first to the last, i.e., the sixth, position. These 6mers were used in Blastp [57] searches at NCBI, as input. The Blastp search parameters (algorithm-options) were as follows: max target sequences 10, no automatic adjustment for short sequences, expect threshold 50, word size 2, max matches in a query range 0, matrix PAM30, gap costs 9, 1, no compositional adjustment. Searches were limited to *Homo sapiens* (taxid: 9606). The resulting alignments were analyzed manually following the search. Alignment results with 6mer matches were selected. Human sequences mimicking Omicron 21K and/or Omicron 21L were selected when there were also human peptides with 6mer matches with the respective nonmutant SARS-CoV-2 sequences.

#### Appendix A.1.2. HLA Affinity Predictions

Human protein-sequences with the aligned 6mers were retrieved from UniProt [58] and NCBI [56] in fasta format, to include 1 or 2 aa from either side of the 6mers before HLA affinity predictions of the 8mers. HLA affinity predictions were completed for the HLA supertype representatives (HLA-A*01:01, HLA-A*02:01, HLA-A*03:01, HLA-A*24:02, HLA-A*26:01, HLA-B*07:02, HLA-B*08:01, HLA-B*15:01, HLA-B*27:05, HLA-B*39:01, HLA-B*40:01, HLA-B*58:01), using NetMHCcons and NetCTLpan. NetMHCcons v1.1 [59] predicts HLA affinities by integrating NetMHC v4.0 [60,61], PickPocket v1.1 [62], and NetMHCpan v4.1 [63]. NetCTLpan (v1.1 [64] and v1.2 [65]) predicts cytotoxic T lymphocyte epitopes. Affinity to HLA meant strong binder (SB) and weak binder (WB) predictions by NetMHCcons, and epitope (E) predictions by NetCTLpan. The threshold for strong binders (SBs) percent rank was 0.5 and that of the weak binders (WBs) was 2, in the case of NetMHCcons. NetCTLpan performed instead epitope (E) assignment, where the threshold for identification was 1, by default. SB peptides below the specified percent ranks and WB peptides between the specified (2 for WB, until 0.5 for SB) percent ranks were identified. Percent rank was the percentile of the predicted binding affinity, which was compared to the distribution of affinities that were calculated on a set of (at least) 200.000 random natural 9mer peptides, as informed at the respective websites: https://services.healthtech.dtu.dk/service.php?NetCTLpan-1.1 (accessed on 17 October 2022) (for NetCTLpan) and https://services.healthtech.dtu.dk/service.php?NetMHCcons-1.1 (accessed on 17 October 2022) (for NetMHCcons) These results were considered significant. The resulting viral/human peptide pairs with high affinities were considered to suggest changes in the autoimmunity risks for the susceptible serotypes, upon getting infected, through lost affinities of the SARS-CoV-2/human peptide pairs or gained affinities of the Omicron/human peptide pairs. We also evaluated changes in the alleles with a high affinity to the viral/human peptide pairs.

#### Appendix A.1.3. Protein Features and Images

Features of the proteins were outlined through the information retrieved from NCBI Entrez [66], UniProtKB/Swiss Prot [58], MalaCards [67,68], SuperPathways [69], and ProteinDB [70,71], collected from the dedicated websites of the GeneCards [72]. Network images were prepared with that information and the data, using Cytoscape [73] version 3.8.2 running with Java 11.0.6. Phylogeny images were generated at covariants.org, on 26 May 2022.

This study additionally separated the results related to sequences with mutations specific for Omicron 21L. When its data was presented separately, the results with sequences of the Omicron variant were commonly denoted either as 21L, standing for the sequences with mutations specific for Omicron 21L, or as “21K + 21K/21L,” standing for the sequences with mutations specific for Omicron 21K plus mutations that are common to both Omicron 21K and Omicron 21L. Therefore, the data with Omicron 21L excludes the data of sequences with mutations common to both Omicron 21K and Omicron 21L.

### Appendix A.2. General Features of the Identified Human Proteins

- Ankyrin and Armadillo Repeat Containing (ANKAR) protein is predicted to be an integral membrane-component and the Gene Ontology annotations related to its gene include binding, binding to the nuclear receptor (Entrez, GeneCards). It is expressed in the heart and pancreatic juice (*information from the estimated protein expression figure*, GeneCards)
- Beta-1,3-galactosyltransferase 5 is a membrane-bound glycoprotein with galactosyltransferase and UDP-galactose:beta-N-acetylglucosamine beta-1,3-galactosyltransferase activities (Entrez, GeneCards).
- HGH1 homolog protein includes Maturity-Onset Diabetes of The Young, Type 3, as the associated disease (GeneCards). It is expressed in plasma, peripheral blood mononuclear cells, heart, bone, and pancreas (*information retrieved from the estimated protein expression figure*, GeneCards).
- HPS1 Biogenesis of Lysosomal Organelles Complex 3 Subunit 1 involves in the Hermansky-Pudlak Syndrome 1. Membrane trafficking and RAB GEF nucleotide exchange are among the pathways of its related superpathways, i.e., vesicle-mediated transport and Rab regulation of trafficking (GeneCards).
- The immunoglobulin (Ig) heavy chain variable region participates in antigen recognition, and membrane-bound immunoglobulins trigger clonal expansion and differentiation of B lymphocytes into Ig-secreting plasma cells (UniProtKB/Swiss-Prot, Entrez). Variable domains of one heavy and one (associated) light chain form two antigen binding sites with high affinity for an antigen (UniProtKB/Swiss-Prot, Entrez). Accordingly, Ig heavy chain and light chain variable regions, and the respective junction regions, are parts of the immune response.
- Mitogen-activated protein kinase kinase 3 is a dual specificity kinase, has transferase and protein tyrosine kinase activities, and its activation by cytokines, mitogens, environmental stress, and insulin is reported while the accumulation of its active form is observed during Ras oncogene expression, followed by oncogenic transformation (GeneCards, UniProtKB/Swiss-Prot, Entrez). Its inhibition is involved in the pathogenesis of *Yersinia pseudotuberculosis* (Entrez).
- Mucin 5AC, Oligomeric Mucus/Gel-Forming, is an extracellular matrix structural constituent, a gel-forming, protective glycoprotein of gastric and respiratory tract epithelia and interacts with *H. pylori* (GeneCards, UniProtKB/Swiss-Prot).
- Nucleoporin 210 is a glycoprotein and is essential for the assembly, fusion, spacing, and integrity of the nuclear pore complex, which regulates macromolecular flow (Entrez, UniProtKB/Swiss-Prot). SARS-CoV-2 infection is among the pathways in which it is involved (Superpathways, GeneCards).
- The pleckstrin homology domain containing A7 enables delta-catenin binding activity in many cellular components, resulting in epithelial cell–cell adhesion, pore complex assembly, and zonula adherens maintenance (Entrez).
- Presenilin 2 is likely a part of the catalytic subunit of the gamma-secretase complex, which is an endoprotease complex catalyzing intramembrane cleavage of integral membrane proteins (e.g., Notch receptors, amyloid-beta precursor) (UniProtKB/Swiss-Prot). It is also suggested to take part in cytoplasmic protein partitioning, intracellular signaling and gene expression, and other cellular events (UniProtKB/Swiss-Prot).
- RB Transcriptional Corepressor Like 2 (*identified as 130K protein in the Blastp alignment document*) is the main regulator of entry into the cell division (UniProtKB/Swiss-Prot). It “enables promoter-specific chromatin binding activity” (Entrez), can lead to (epigenetic) transcriptional repression by recruiting chromatin-modifier enzymes, histone methyltransferases, and may be involved in the transforming capacity of the adenovirus E1A protein, as well as acting as a tumor suppressor (GeneCards, UniProtKB/Swiss-Prot).
- Rho guanine nucleotide exchange factor 4 complexes with G proteins; acts as guanine nucleotide exchange factor; and stimulates Rho-dependent signals, thus participating in many extracellularly stimulated processes, as well as tumor angiogenesis (Entrez, UniProtKB/Swiss-Prot). It may play a role in intestinal adenoma formation and tumor progression (UniProtKB/Swiss-Prot).
- Ring Finger Protein 10 (*identified as an unnamed protein product in the Blastp alignment document*) related Gene Ontology annotations include activity of ubiquitin-protein transferase, and binding of transcription cis-regulatory region, and is involved in protein–protein interactions (GeneCards, Entrez). It is a Schwann cell differentiation and myelination regulator (UniProtKB/Swiss-Prot). *Please note that the identified “unnamed protein product” had a similar sequence to the Ring Finger Protein 10 (RNF 10), although the RFN10 did not contain the region with the sequence in our results. However, the rest of its sequence was the same. Hence, the disorders, pathways, and expression sites related to RNF10 were included in the presented data.*
- Signaling Lymphocytic Activation Molecule Family Member 1 is a self-ligand receptor of the signaling lymphocytic activation molecule (SLAM) family and is thus involved in modulation of the immune cell activation and differentiation, innate and adaptive immune response regulation and interconnection (UniProtKB/Swiss-Prot).
- Solute carrier family 12 member 4 (human KCC1 structure determined in KCl and detergent GDN) mediates the coupled transport of potassium and chloride ions across the plasma membrane, upon activation by the swelling of the cell (Entrez, UniProtKB/Swiss-Prot).
- Solute carrier family 22 member 6 is involved in the sodium-dependent transport and the renal elimination of endogenous and exogenous organic anions, some of which can be toxic; exchanges organic anions with a coupling; and mediates several sodium-independent uptakes (UniProtKB/Swiss-Prot, Entrez).
- Solute carrier family 25 member 27 (mitochondrial uncoupling protein 4) uncouples oxidative phosphorylation from ATP synthesis, and energy is dissipated in the form of heat as a result (UniProtKB/Swiss-Prot).
- Zinc finger protein 462 is probably involved in transcriptional regulation through the structure and organization of chromatin, leading to the regulation of, for example, pluripotency and differentiation of embryonic stem cells, and the development and differentiation of neurons (Entrez, UniProtKB/Swiss-Prot).

**Table A1:** Disorders associated with the identified proteins, in the order of ascending number of associated diseases. (Information retrieved from the resources detailed in the Materials and Methods).

**Protein** Disorder(s)
**HGH1 Homolog**
Maturity-Onset Diabetes Of The Young, Type 3
**Solute Carrier Family 25 Member 27**
Ecthyma
Hepatocellular Carcinoma
**Rho Guanine Nucleotide Exchange Factor 4**
Epidermolysis Bullosa Simplex 1a, Generalized Severe
Locked-In Syndrome
**Beta-1,3-Galactosyltransferase 5**
Mood Disorder
Pancreatic Cancer
**Ring Finger Protein 10**
Spastic Paraplegia 80, Autosomal Dominant
superficial keratitis
**Ankyrin And Armadillo Repeat Containing**
Cowden Syndrome
Cowden Syndrome 1
Hemochromatosis, Type 4
Loeys-Dietz Syndrome
**Solute Carrier Family 22 Member 6**
Acute Kidney Failure
Fanconi Syndrome
Fanconi-Like Syndrome
Methotrexate Toxicity
N-Acetylglutamate Synthase Deficiency
Tubulointerstitial Kidney Disease, Autosomal Dominant, 1
**Mitogen-Activated Protein Kinase Kinase 3**
Breast Cancer
Cardiomyopathy, Familial Hypertrophic, 25
Colorectal Cancer
Cutaneous Anthra
Inhalation Anthrax
Parkinson Disease, Late-Onset
Von Hippel-Lindau Syndrome
**Solute Carrier Family 12 Member 4**
Agenesis Of The Corpus Callosum with Peripheral Neuropathy
Bartter Disease
Chronic Cervicitis
Fish-Eye Disease
Hemoglobin C Disease
Hypomagnesemia 4, Renal
Sickle Cell Disease
**Pleckstrin Homology Domain Containing A7**
Blepharocheilodontic Syndrome 1
Cleft Lip With Or Without Cleft Palate
Glaucoma, Primary Open Angle
Marshall Syndrome
Nanophthalmos
Primary Angle-Closure Glaucoma
Renal Adenoma
Stickler Syndrome
**Zinc Finger Protein 462**
Acrofacial Dysostosis 1, Nager Type
Craniosynostosis
Hypermobility Syndrome
Metopic Ridging-Ptosis-Facial Dysmorphism Syndrome
Premature Menopause
Ptosis
Syndromic Intellectual Disability
Weiss-Kruszka Syndrome
**Nucleoporin 210**
Achalasia-Addisonianism-Alacrima Syndrome
Amelogenesis Imperfecta, Type Ie 64
Autoimmune Cholangitis
Autoimmune Disease Of Gastrointestinal Tract
Cholangitis
Cholangitis, Primary Sclerosing
Crest Syndrome
Peliosis Hepatis
Primary Biliary Cholangitis
**HPS1 Biogenesis Of Lysosomal Organelles Complex 3 Subunit 1**
Albinism
Chediak-Higashi Syndrome
Hermansky-Pudlak Syndrome
Hermansky-Pudlak Syndrome 1
Hermansky-Pudlak Syndrome Due To Bloc-3 Deficiency
Melanoma In Congenital Melanocytic Nevus
Nonspecific Interstitial Pneumonia
Oculocutaneous Albinism
Pulmonary Fibrosis
**RB Transcriptional Corepressor Like 2**
Bilateral Retinoblastoma
Brunet-Wagner Neurodevelopmental Syndrome 6 109
Burkitt Lymphoma
Extraocular Retinoblastoma
Eye Disease
Hypoglycemia, Leucine-Induced
Ocular Cancer
Osteogenic Sarcoma
Papilloma
Retinal Cancer
Retinoblastoma
Spastic Paraplegia 27, Autosomal Recessive
Spastic Paraplegia 36, Autosomal Dominant
Unilateral Retinoblastoma
**Signaling Lymphocytic Activation Molecule Family Member 1**
Dysgammaglobulinemia
Herpangina
Immune Deficiency Disease
Leukemia, Acute Myeloid
Lymphoma, Hodgkin, Classic
Lymphoproliferative Syndrome 2
Lymphoproliferative Syndrome
Lymphoproliferative Syndrome, X-Linked, 1
Lymphoproliferative Syndrome, X-Linked, 2
Measles
Pfeiffer Syndrome
Postinfectious Encephalitis
Selective Immunoglobulin Deficiency Disease
Subacute Sclerosing Panencephalitis
Systemic Lupus Erythematosus
Trochlear Nerve Disease
Viral Infectious Disease
**Presenilin 2**
Acute Conjunctivitis
Acute Hemorrhagic Conjunctivitis
Agraphia
Alzheimer Disease 2
Alzheimer Disease 3
Alzheimer Disease 4
Alzheimer Disease, Familial, 1
Alzheimer’S Disease 1
Amyloidosis
Amyotrophic Lateral Sclerosis 1
Apperceptive Agnosia
Basal Ganglia Calcification
Breast Cancer
Cardiomyopathy, Dilated, 1v
Cerebral Amyloid Angiopathy, App-Related
Cerebral Amyloid Angiopathy, Cst3-Related
Cerebral Amyloid Angiopathy, Itm2b-Related
Chromosomal Disease
Chromosomal Duplication Syndrome
Conjunctival Folliculosis
Dementia
Dementia, Lewy Body
Dilated Cardiomyopathy
Disease Of Mental Health
Dyscalculia
Early-Onset Autosomal Dominant Alzheimer Disease
Familial Isolated Dilated Cardiomyopathy
Frontotemporal Dementia
Gerstmann Syndrome
Gerstmann-Straussler Disease
Huntington Disease-Like Syndrome
Hyperlucent Lung
Ideomotor Apraxia
Leber Congenital Amaurosis 7
Mild Cognitive Impairment
Mitochondrial Dna Depletion Syndrome 12b
Movement Disease
Nervous System Disease
Pharyngoconjunctival Fever
Pick Disease Of Brain
Polycystic Lipomembranous Osteodysplasia With Sclerosing Leukoencephalopathy 1
Posterior Cortical Atrophy
Prosopagnosia
Shipyard Eye
Simultanagnosia
Speech And Communication Disorders
Supranuclear Palsy, Progressive, 1
Tactile Agnosia
Visual Agnosia
**Mucin 5AC, Oligomeric Mucus/Gel-Forming**
Acute Cholangitis
Acute Dacryocystitis
Acute Inflammation Of Lacrimal Passage
Adenocarcinoma
Adenoma
Ampulla Of Vater Adenocarcinoma
Ampulla Of Vater Cancer
Anal Canal Adenocarcinoma
Anal Gland Adenocarcinoma
Androgen Insensitivity Syndrome
Anus Adenocarcinoma
Appendix Cancer
Appendix Disease
Asthma
Atopic Keratoconjunctivitis
Barrett Esophagus
Bile Duct Adenocarcinoma
Bile Duct Cancer
Bile Duct Cystadenocarcinoma
Bile Duct Mucinous Adenocarcinoma
Bile Duct Mucoepidermoid Carcinoma
Bile Reflux
Biliary Papillomatosis
Biliary Tract Benign Neoplasm
Biliary Tract Disease
Bladder Benign Neoplasm
Blepharitis
Breast Mucoepidermoid Carcinoma
Bronchial Disease
Bronchiolo-Alveolar Adenocarcinoma
Cap Polyposis
Cholangiocarcinoma
Cholecystitis
Chronic Asthma
Chronic Conjunctivitis
Chronic Ethmoiditis
Colloid Carcinoma Of The Pancreas
Colorectal Cancer
Colorectal Cancer, Hereditary Nonpolyposis, Type 8
Common Cold
Complete Androgen Insensitivity Syndrome
Conjunctival Disease
Corneal Ulcer
Cystadenocarcinoma
Cystadenoma
Cystic Fibrosis
Cystic Teratoma
Dacryocystitis
Diverticulitis
Dry Eye Syndrome
Duodenum Adenocarcinoma
Duodenum Cancer
Duodenum Disease
Endobronchial Lipoma
Endocervical Adenocarcinoma
Endometrial Mucinous Adenocarcinoma
Exercise-Induced Bronchoconstriction
Eye Disease
Eyelid Disease
Filamentary Keratitis
Gastric Cancer
Gastric Tubular Adenocarcinoma
Inflammatory Bowel Disease
Interstitial Lung Disease 2
Intrahepatic Biliary Papillomatosis
Intrahepatic Cholangiocarcinoma
Keratoconjunctivitis Sicca
Keratoconjunctivitis
Lacrimal Apparatus Disease
Limbal Stem Cell Deficiency
Lung Cancer Susceptibility 3
Lung Disease
Lung Mucoepidermoid Carcinoma
Meconium Ileus
Microinvasive Gastric Cancer
Middle Ear Disease
Mucinous Adenocarcinoma
Mucinous Cystadenocarcinoma Of Pancreas
Mucinous Intrahepatic Cholangiocarcinoma
Mucoepidermoid Carcinoma
Neurotrophic Keratoconjunctivitis
Otitis Media
Ovarian Cancer
Ovarian Cystadenocarcinoma
Ovarian Mucinous Adenocarcinoma
Ovarian Mucinous Neoplasm
Pancreatic Cancer
Pancreatic Ductal Carcinoma
Pancreatic Mucinous Cystadenoma
Pancreatic Signet Ring Cell Adenocarcinoma
Poikiloderma With Neutropenia
Polyposis, Skin Pigmentation, Alopecia, And Fingernail Changes
Primary Ciliary Dyskinesia
Pseudomyxoma Peritonei
Pulmonary Disease, Chronic Obstructive
Punctate Epithelial Keratoconjunctivitis
Respiratory Allergy
Respiratory Failure
Senile Ectropion
Severe Cutaneous Adverse Reaction
Signet Ring Cell Adenocarcinoma
Silo Filler’S Disease
Small Intestine Adenocarcinoma
Small Intestine Cancer
Solid Adenocarcinoma With Mucin Production
Status Asthmaticus
T2-Low Asthma
Tubular Adenocarcinoma
Urinary Bladder Villous Adenoma
Vernal Conjunctivitis
Villous Adenoma

**Table A2:** Associated superpathways of the identified proteins, in the order of ascending number of associated superpathways. (Information retrieved from the resources detailed in the Materials and Methods).

**Superpathway** Protein(s)
**Jak-Stat Signaling Pathway**
Mitogen-Activated Protein Kinase Kinase 3
**16p11.2 Proximal Deletion Syndrome**
Mitogen-Activated Protein Kinase Kinase 3
**JNK (c-Jun kinases) Phosphorylation and Activation Mediated by Activated Human TAK1**
Mitogen-Activated Protein Kinase Kinase 3
**4-Hydroxytamoxifen, Dexamethasone, and Retinoic Acids Regulation of p27 Expression**
Mitogen-Activated Protein Kinase Kinase 3
**LKB1 Signaling Events**
Presenilin 2
**ABH and Lewis Epitopes Biosynthesis from Type 1 Precursor Disaccharide**
Beta-1,3-Galactosyltransferase 5
**Macrophage Differentiation and Growth Inhibition by METS**
RB Transcriptional Corepressor Like 2
**Acyclovir/Ganciclovir Pathway, Pharmacokinetics/Pharmacodynamics**
Solute Carrier Family 22 Member 6
**Malignant Pleural Mesothelioma**
Mitogen-Activated Protein Kinase Kinase 3
**Akt Signaling**
Mitogen-Activated Protein Kinase Kinase 3
**MAP Kinase Signaling**
Mitogen-Activated Protein Kinase Kinase 3
**Alzheimers Disease Pathway**
Presenilin 2
**MAPK Signaling Pathway**
Mitogen-Activated Protein Kinase Kinase 3
**Angiopoietin-Like Protein 8 Regulatory Pathway**
Mitogen-Activated Protein Kinase Kinase 3
**MAPK Signaling: Oxidative Stress**
Mitogen-Activated Protein Kinase Kinase 3
**Apoptosis and Survival_Anti-Apoptotic Action of Nuclear ESR1 and ESR2**
Mitogen-Activated Protein Kinase Kinase 3
**Mesodermal Commitment Pathway**
Zinc Finger Protein 462
**Zidovudine Pathway, Pharmacokinetics/Pharmacodynamics**
Solute Carrier Family 22 Member 6
**Methotrexate Pathway, Pharmacokinetics**
Solute Carrier Family 22 Member 6
**Bacterial Infections in CF Airways**
Mitogen-Activated Protein Kinase Kinase 3
**MicroRNAs in Cardiomyocyte Hypertrophy**
Mitogen-Activated Protein Kinase Kinase 3
**Beta-2 Adrenergic-Dependent CFTR Expression**
Mitogen-Activated Protein Kinase Kinase 3
**MIF Mediated Glucocorticoid Regulation**
Mitogen-Activated Protein Kinase Kinase 3
**Blood Group Systems Biosynthesis**
Beta-1,3-Galactosyltransferase 5
**Mitotic G1 Phase and G1/S Transition**
RB Transcriptional Corepressor Like 2
**Canonical and Non-Canonical Notch Signaling**
Presenilin 2
**Monoamine Transport**
RB Transcriptional Corepressor Like 2
**Cell adhesion_Plasmin Signaling**
Mitogen-Activated Protein Kinase Kinase 3
**Nanog in Mammalian ESC Pluripotency**
Mitogen-Activated Protein Kinase Kinase 3
**Cell Cycle Regulation of G1/S Transition (Part 1)**
RB Transcriptional Corepressor Like 2
**Nervous System Development**
Presenilin 2
**Cellular Roles of Anthrax Toxin**
Mitogen-Activated Protein Kinase Kinase 3
**Neuropathic Pain-Signaling in Dorsal Horn Neurons**
Mitogen-Activated Protein Kinase Kinase 3
**Ceramide Pathway**
Mitogen-Activated Protein Kinase Kinase 3
**Neuroscience**
Presenilin 2
**CLEC7A (Dectin-1) Signaling**
Mucin 5AC, Oligomeric Mucus/Gel-Forming
**NFAT and Cardiac Hypertrophy**
Mitogen-Activated Protein Kinase Kinase 3
**Colorectal Cancer Metastasis**
Mitogen-Activated Protein Kinase Kinase 3
**NgR-p75(NTR)-Mediated Signaling**
Rho Guanine Nucleotide Exchange Factor 4
**CXCR3-Mediated Signaling Events**
Mitogen-Activated Protein Kinase Kinase 3
**Non-Canonical Wnt Pathway**
Mitogen-Activated Protein Kinase Kinase 3
**Death Receptor Signaling**
Mitogen-Activated Protein Kinase Kinase 3
**Notch Pathway**
Presenilin 2
**Dendritic Cells Developmental Lineage Pathway**
Signaling Lymphocytic Activation Molecule Family Member 1
**Notch Signaling (Qiagen)**
Presenilin 2
**Development A3 Receptor Signaling**
Mitogen-Activated Protein Kinase Kinase 3
**Notch Signaling (WikiPathways)**
Presenilin 2
**Development FGFR Signaling Pathway**
Mitogen-Activated Protein Kinase Kinase 3
**Notch Signaling Pathways**
Presenilin 2
**Development Notch Signaling Pathway**
Presenilin 2
**NOTCH2 Activation and Transmission of Signal to the Nucleus**
Presenilin 2
**Development_TGF-beta Receptor Signaling**
Mitogen-Activated Protein Kinase Kinase 3
**O-linked Glycosylation of Mucins**
Mucin 5AC, Oligomeric Mucus/Gel-Forming
**Diseases of Glycosylation**
Mucin 5AC, Oligomeric Mucus/Gel-Forming
**p38 MAPK signaling pathway (Pathway Interaction Database)**
Mitogen-Activated Protein Kinase Kinase 3
**DNA Damage**
RB Transcriptional Corepressor Like 2
**P38 MAPK Signaling Pathway (sino)**
Mitogen-Activated Protein Kinase Kinase 3
**Endoderm Differentiation**
Mitogen-Activated Protein Kinase Kinase 3
**p70S6K Signaling**
Mitogen-Activated Protein Kinase Kinase 3
**Epithelial to Mesenchymal Transition in Colorectal Cancer**
Mitogen-Activated Protein Kinase Kinase 3
**Phospholipase-C Pathway**
Rho Guanine Nucleotide Exchange Factor 4
**FoxO Family Signaling**
RB Transcriptional Corepressor Like 2
**Physiological and Pathological Hypertrophy of the Heart**
Mitogen-Activated Protein Kinase Kinase 3
**G0 and Early G1**
RB Transcriptional Corepressor Like 2
**PI3K-Akt Signaling Pathway**
RB Transcriptional Corepressor Like 2
**G-AlphaQ Signaling**
Rho Guanine Nucleotide Exchange Factor 4
**Pre-NOTCH Expression and Processing**
Presenilin 2
**Gene Silencing by RNA**
Nucleoporin 210
**Presenilin-Mediated Signaling**
Presenilin 2
**Glycolysis (REACTOME)**
Nucleoporin 210
**Processing of Capped Intron-Containing Pre-mRNA**
Nucleoporin 210
**GPER1 Signaling**
Mitogen-Activated Protein Kinase Kinase 3
**Rab Regulation of Trafficking**
HPS1 Biogenesis Of Lysosomal Organelles Complex 3 Subunit 1
**G-Protein Signaling Regulation of p38 and JNK Signaling Mediated by G-proteins**
Mitogen-Activated Protein Kinase Kinase 3
**RAC1 GTPase Cycle**
Rho Guanine Nucleotide Exchange Factor 4
**Guidance Cues and Growth Cone Motility**
Rho Guanine Nucleotide Exchange Factor 4
**RAF/MAP Kinase Cascade**
Presenilin 2
**Hematopoietic Stem Cells and Lineage-Specific Markers**
Signaling Lymphocytic Activation Molecule Family Member 1
**Regulation of Actin Cytoskeleton**
Rho Guanine Nucleotide Exchange Factor 4
**HIV Life Cycle**
Nucleoporin 210
**Regulation of p38-alpha and p38-beta**
Mitogen-Activated Protein Kinase Kinase 3
**IL-9 Signaling Pathways**
Mitogen-Activated Protein Kinase Kinase 3
**Regulation of TP53 Activity**
RB Transcriptional Corepressor Like 2
**Immune Response_Role of Integrins in NK Cells Cytotoxicity**
Mitogen-Activated Protein Kinase Kinase 3
**Respiratory Electron Transport, ATP Synthesis by Chemiosmotic Coupling, and Heat Production by Uncoupling Proteins**
Solute Carrier Family 25 Member 27
**Influenza Infection**
Nucleoporin 210
**RhoA Signaling Pathway**
Mitogen-Activated Protein Kinase Kinase 3
**Integrin-Mediated Cell Adhesion**
Mitogen-Activated Protein Kinase Kinase 3
**RHOC GTPase Cycle**
Rho Guanine Nucleotide Exchange Factor 4
**4-1BB Pathway**
Mitogen-Activated Protein Kinase Kinase 3
**RhoGDI Pathway**
Rho Guanine Nucleotide Exchange Factor 4
**Actin Nucleation by ARP-WASP Complex**
Rho Guanine Nucleotide Exchange Factor 4
**SARS-CoV-2 Infection**
Nucleoporin 210
**Alzheimer’s Disease and miRNA Effects**
Presenilin 2
**Senescence and Autophagy in Cancer**
Mitogen-Activated Protein Kinase Kinase 3
**Antiviral Mechanism by IFN-Stimulated Genes**
Nucleoporin 210
**Serotonin HTR1 Group and FOS Pathway**
Mitogen-Activated Protein Kinase Kinase 3
**B Cell Receptor Signaling Pathway**
Mitogen-Activated Protein Kinase Kinase 3
**Sertoli-Sertoli Cell Junction Dynamics**
Mitogen-Activated Protein Kinase Kinase 3
**Beta-Adrenergic Signaling**
Mitogen-Activated Protein Kinase Kinase 3
**Signaling by ERBB4**
Presenilin 2
**CCR3 Pathway in Eosinophils**
Mitogen-Activated Protein Kinase Kinase 3
**Signaling by NOTCH3**
Presenilin 2
**Cellular Response to Heat Stress**
Nucleoporin 210
**Signaling by Receptor Tyrosine Kinases**
Presenilin 2
**Chromatin Regulation/Acetylation**
RB Transcriptional Corepressor Like 2
**Signaling by Rho GTPases**
Rho Guanine Nucleotide Exchange Factor 4
**Constitutive Signaling by NOTCH1 HD+PEST Domain Mutants**
Presenilin 2
**Signaling by Slit**
Rho Guanine Nucleotide Exchange Factor 4
**Defective Binding of RB1 Mutants to E2F1,(E2F2, E2F3)**
RB Transcriptional Corepressor Like 2
**Signaling Events Mediated by HDAC Class I**
Nucleoporin 210
**Development Beta-Adrenergic Receptors Regulation of ERK**
Mitogen-Activated Protein Kinase Kinase 3
**Signaling Events Mediated by Hepatocyte Growth Factor Receptor (c-Met)**
Rho Guanine Nucleotide Exchange Factor 4
**Development VEGF signaling via VEGFR2—Generic Cascades**
Mitogen-Activated Protein Kinase Kinase 3
**Signaling Events Mediated by VEGFR1 and VEGFR2**
Mitogen-Activated Protein Kinase Kinase 3
**Disorders of Transmembrane Transporters**
Nucleoporin 210
**Signaling Mediated by p38-gamma and p38-delta**
Mitogen-Activated Protein Kinase Kinase 3
**EPH-Ephrin Signaling**
Presenilin 2
**Stabilization and Expansion of the E-cadherin Adherens Junction**
Pleckstrin Homology Domain Containing A7
**FOXO-mediated Transcription**
RB Transcriptional Corepressor Like 2
**Statin Pathway—Generalized, Pharmacokinetics**
Solute Carrier Family 22 Member 6
**GDNF-Family Ligands and Receptor Interactions**
Mitogen-Activated Protein Kinase Kinase 3
**Sumoylation by RanBP2 Regulates Transcriptional Repression**
Nucleoporin 210
**GPCR Downstream Signalling**
Rho Guanine Nucleotide Exchange Factor 4
**superpathway of glycosphingolipids biosynthesis**
Beta-1,3-Galactosyltransferase 5
**G-protein Signaling—Regulation of RAC1 Activity**
Rho Guanine Nucleotide Exchange Factor 4
**Sweet Taste Signaling**
Mitogen-Activated Protein Kinase Kinase 3
**HIF1Alpha Pathway**
Mitogen-Activated Protein Kinase Kinase 3
**Tacrolimus/Cyclosporine Pathway, Pharmacodynamics**
Mitogen-Activated Protein Kinase Kinase 3
**Immune Response Fc Epsilon RI Pathway**
Mitogen-Activated Protein Kinase Kinase 3
**TCR Signaling (Qiagen)**
Mitogen-Activated Protein Kinase Kinase 3
**Integrin Pathway**
Mitogen-Activated Protein Kinase Kinase 3
**Tenofovir/Adefovir Pathway, Pharmacokinetics**
Solute Carrier Family 22 Member 6
**A-beta Plaque Formation and APP Metabolism**
Presenilin 2
**Termination of O-glycan Biosynthesis**
Mucin 5AC, Oligomeric Mucus/Gel-Forming
**AMPK Enzyme Complex Pathway**
Mitogen-Activated Protein Kinase Kinase 3
**TGF-beta Signaling Pathways**
Mitogen-Activated Protein Kinase Kinase 3
**BAFF in B-Cell Signaling**
Mitogen-Activated Protein Kinase Kinase 3
**The Fatty Acid Cycling Model**
Solute Carrier Family 25 Member 27
**Cell cycle**
RB Transcriptional Corepressor Like 2
**Thermogenesis**
Mitogen-Activated Protein Kinase Kinase 3
**CNTF Signaling**
Mitogen-Activated Protein Kinase Kinase 3
**TNF Signaling**
Mitogen-Activated Protein Kinase Kinase 3
**Development A2A Receptor Signaling**
Mitogen-Activated Protein Kinase Kinase 3
**TNF Superfamily—Human Ligand-Receptor Interactions and their Associated Functions**
Mitogen-Activated Protein Kinase Kinase 3
**Diseases Associated with O-glycosylation of Proteins**
Mucin 5AC, Oligomeric Mucus/Gel-Forming
**Toll Comparative Pathway**
Mitogen-Activated Protein Kinase Kinase 3
**fMLP Pathway**
Rho Guanine Nucleotide Exchange Factor 4
**Toll-Like receptor Signaling Pathways**
Mitogen-Activated Protein Kinase Kinase 3
**Globo Sphingolipid Metabolism**
Beta-1,3-Galactosyltransferase 5
**TP53 Regulates Transcription of Cell Cycle Genes**
RB Transcriptional Corepressor Like 2
**HCMV Infection**
Nucleoporin 210
**TRAF6 Mediated Induction of NFkB and MAP Kinases upon TLR7/8 or 9 Activation**
Mitogen-Activated Protein Kinase Kinase 3
**Inclusion Body Myositis**
Presenilin 2
**Translation Insulin Regulation of Translation**
Mitogen-Activated Protein Kinase Kinase 3
**Adipogenesis**
RB Transcriptional Corepressor Like 2
**Transport of Mature Transcript to Cytoplasm**
Nucleoporin 210
**Breast Cancer Pathway**
Mitogen-Activated Protein Kinase Kinase 3
**Transport of the SLBP Independent Mature mRNA**
Nucleoporin 210
**Cytoskeleton Remodeling Regulation of Actin Cytoskeleton by Rho GTPases**
Presenilin 2
**Trk Receptor Signaling Mediated by the MAPK Pathway**
Mitogen-Activated Protein Kinase Kinase 3
**DNA Damage Response (Only ATM Dependent)**
RB Transcriptional Corepressor Like 2
**tRNA processing**
Nucleoporin 210
**G-protein Signaling RAC1 in Cellular Process**
Mitogen-Activated Protein Kinase Kinase 3
**Uptake and Actions of Bacterial Toxins**
Mitogen-Activated Protein Kinase Kinase 3
**Interferon Gamma Signaling**
Nucleoporin 210
**Uricosurics Pathway, Pharmacodynamics**
Solute Carrier Family 22 Member 6
**Cellular Senescence**
Mitogen-Activated Protein Kinase Kinase 3
**VEGF Pathway (Qiagen)**
Mitogen-Activated Protein Kinase Kinase 3
**G12-G13 in Cellular Signaling**
Mitogen-Activated Protein Kinase Kinase 3
**VEGF Signaling Pathway**
Mitogen-Activated Protein Kinase Kinase 3
**Atenolol Pathway, Pharmacokinetics**
Solute Carrier Family 22 Member 6
**Vesicle-mediated Transport**
HPS1 Biogenesis Of Lysosomal Organelles Complex 3 Subunit 1
**IL12-mediated Signaling Events**
Mitogen-Activated Protein Kinase Kinase 3
**Vitamin D in Inflammatory Diseases**
Mitogen-Activated Protein Kinase Kinase 3
**Development Ligand-independent Activation of ESR1 and ESR2**
Mitogen-Activated Protein Kinase Kinase 3
**Wnt/Hedgehog/Notch**
Presenilin 2
**MAPK-Erk Pathway**
Mitogen-Activated Protein Kinase Kinase 3
RB Transcriptional Corepressor Like 2
**Transport of Inorganic Cations/Anions and Amino Acids/Oligopeptides**
Solute Carrier Family 12 Member 4
Solute Carrier Family 22 Member 6
**IL-17 Family Signaling Pathways**
Mitogen-Activated Protein Kinase Kinase 3
Mucin 5AC, Oligomeric Mucus/Gel-Forming
**Cytokine Signaling in Immune System**
Mitogen-Activated Protein Kinase Kinase 3
Nucleoporin 210
**TGF-Beta Pathway**
Mitogen-Activated Protein Kinase Kinase 3
Rho Guanine Nucleotide Exchange Factor 4
**Signal Transduction**
Presenilin 2
Rho Guanine Nucleotide Exchange Factor 4
**Gene expression (Transcription)**
Nucleoporin 210
RB Transcriptional Corepressor Like 2
**Regulation of Activated PAK-2p34 by Proteasome Mediated Degradation**
Presenilin 2
RB Transcriptional Corepressor Like 2
**Toll-like Receptor Signaling Pathway**
Mitogen-Activated Protein Kinase Kinase 3
Signaling Lymphocytic Activation Molecule Family Member 1
**Metabolism of Proteins**
Mucin 5AC, Oligomeric Mucus/Gel-Forming
Nucleoporin 210
**Proximal Tubule Transport**
Solute Carrier Family 12 Member 4
Solute Carrier Family 22 Member 6
**Glycosaminoglycan Metabolism**
Beta-1,3-Galactosyltransferase 5
Nucleoporin 210
**CREB Pathway**
Mitogen-Activated Protein Kinase Kinase 3
Rho Guanine Nucleotide Exchange Factor 4
**Cell Cycle, Mitotic**
Nucleoporin 210
RB Transcriptional Corepressor Like 2
**Cellular Responses to Stimuli**
Mitogen-Activated Protein Kinase Kinase 3
Nucleoporin 210
**GPCR Pathway**
Mitogen-Activated Protein Kinase Kinase 3
Rho Guanine Nucleotide Exchange Factor 4
**Apoptotic Pathways in Synovial Fibroblasts**
Mitogen-Activated Protein Kinase Kinase 3
Rho Guanine Nucleotide Exchange Factor 4
**Interferon Pathway**
Mitogen-Activated Protein Kinase Kinase 3
Rho Guanine Nucleotide Exchange Factor 4
**Prolactin Signaling**
Mitogen-Activated Protein Kinase Kinase 3
Presenilin 2
**IL-1 Family Signaling Pathways**
Mitogen-Activated Protein Kinase Kinase 3
Mucin 5AC, Oligomeric Mucus/Gel-Forming
**Thyroid Stimulating Hormone (tsh) Signaling Pathway**
Mitogen-Activated Protein Kinase Kinase 3
RB Transcriptional Corepressor Like 2
**p75 NTR Receptor-Mediated Signalling**
Presenilin 2
Rho Guanine Nucleotide Exchange Factor 4
**Innate Immune System**
Mucin 5AC, Oligomeric Mucus/Gel-Forming
Mitogen-Activated Protein Kinase Kinase 3
Nucleoporin 210
**ERK Signaling**
Presenilin 2
Mitogen-Activated Protein Kinase Kinase 3
Rho Guanine Nucleotide Exchange Factor 4
**Metabolism**
Nucleoporin 210
Beta-1,3-Galactosyltransferase 5
Solute Carrier Family 25 Member 27
**Disease**
Mucin 5AC, Oligomeric Mucus/Gel-Forming
Nucleoporin 210
Mitogen-Activated Protein Kinase Kinase 3
Presenilin 2

**Table A3:** Tissues expressing the identified proteins, in ascending order according to the total average normalized intensities. (Information retrieved from the resources detailed in the Materials and Methods).

**Expressing Tissue** Protein(s)	**Total Average Normalized Intensity** Average Normalized Intensity
**Cervical Mucosa**	**3.02**
Mucin 5AC, Oligomeric Mucus/Gel-Forming	3.02
**Osteosarcoma Cell**	**3.73**
Solute Carrier Family 12 Member 4	3.73
**Bone**	**3.85**
Zinc Finger Protein 462	3.85
**Oral Epithelium**	**4.09**
Mitogen-Activated Protein Kinase Kinase 3	4.09
**Adipocyte**	**4.27**
Mitogen-Activated Protein Kinase Kinase 3	4.27
**Uterine Cervix**	**4.42**
Mitogen-Activated Protein Kinase Kinase 3	4.42
**Uterus**	**4.67**
Mitogen-Activated Protein Kinase Kinase 3	4.67
**Skin**	**5.31**
Mitogen-Activated Protein Kinase Kinase 3	5.31
**Prefrontal Cortex**	**8.39**
Nucleoporin 210	3.47
Solute Carrier Family 25 Member 27	4.92
**Breast**	**9.12**
Mitogen-Activated Protein Kinase Kinase 3	4.77
Nucleoporin 210	4.35
**Spermatozoon**	**9.98**
Nucleoporin 210	5.64
RB Transcriptional Corepressor Like 2	4.34
**Cardia**	**11.24**
Mitogen-Activated Protein Kinase Kinase 3	5.31
Mucin 5AC, Oligomeric Mucus/Gel-Forming	5.93
**Spinal Cord**	**13.50**
Mitogen-Activated Protein Kinase Kinase 3	3.99
Nucleoporin 210	3.47
Pleckstrin Homology Domain Containing A7	3.02
Rho Guanine Nucleotide Exchange Factor 4	3.02
**Natural Killer Cell**	**13.58**
Mitogen-Activated Protein Kinase Kinase 3	5.29
Nucleoporin 210	5.41
RB Transcriptional Corepressor Like 2	2.88
**Gut**	**17.30**
Mitogen-Activated Protein Kinase Kinase 3	4.10
Mucin 5AC, Oligomeric Mucus/Gel-Forming	5.55
Nucleoporin 210	4.58
Pleckstrin Homology Domain Containing A7	3.07
**Monocyte**	**17.99**
Mitogen-Activated Protein Kinase Kinase 3	5.42
Nucleoporin 210	5.45
RB Transcriptional Corepressor Like 2	3.62
Solute Carrier Family 12 Member 4	3.50
**Pancreatic Islet**	**18.45**
Mitogen-Activated Protein Kinase Kinase 3	4.85
Nucleoporin 210	4.89
Pleckstrin Homology Domain Containing A7	4.35
Solute Carrier Family 12 Member 4	4.37
**Blood Platelet**	**20.71**
HPS1 Biogenesis Of Lysosomal Organelles Complex 3 Subunit 1	4.15
Mitogen-Activated Protein Kinase Kinase 3	5.04
Mucin 5AC, Oligomeric Mucus/Gel-Forming	1.82
Nucleoporin 210	2.14
Ring Finger Protein 10	2.66
Solute Carrier Family 12 Member 4	2.52
Zinc Finger Protein 462	2.38
**Helper T-Lymphocyte**	**22.29**
Mitogen-Activated Protein Kinase Kinase 3	5.33
Nucleoporin 210	5.71
RB Transcriptional Corepressor Like 2	3.67
Signaling Lymphocytic Activation Molecule Family Member 1	4.33
Zinc Finger Protein 462	3.25
**Heart**	**22.30**
Mitogen-Activated Protein Kinase Kinase 3	4.45
Nucleoporin 210	4.03
Pleckstrin Homology Domain Containing A7	4.64
RB Transcriptional Corepressor Like 2	2.84
Solute Carrier Family 12 Member 4	3.75
Solute Carrier Family 25 Member 27	2.60
**Colonic Epithelial Cell**	**23.36**
Beta-1,3-Galactosyltransferase 5	5.33
Mitogen-Activated Protein Kinase Kinase 3	4.61
Mucin 5AC, Oligomeric Mucus/Gel-Forming	3.57
Nucleoporin 210	5.36
Solute Carrier Family 25 Member 27	4.50
**Ovary**	**23.41**
Mitogen-Activated Protein Kinase Kinase 3	4.14
Nucleoporin 210	4.88
Pleckstrin Homology Domain Containing A7	5.25
RB Transcriptional Corepressor Like 2	3.16
Solute Carrier Family 12 Member 4	3.73
Zinc Finger Protein 462	2.25
**Urinary Bladder**	**23.79**
Mitogen-Activated Protein Kinase Kinase 3	4.68
Mucin 5AC, Oligomeric Mucus/Gel-Forming	3.03
Nucleoporin 210	3.93
Pleckstrin Homology Domain Containing A7	4.16
RB Transcriptional Corepressor Like 2	3.60
Solute Carrier Family 12 Member 4	4.39
**B-lymphocyte**	**23.82**
HPS1 Biogenesis Of Lysosomal Organelles Complex 3 Subunit 1	3.49
Mitogen-Activated Protein Kinase Kinase 3	5.74
Nucleoporin 210	5.55
RB Transcriptional Corepressor Like 2	4.47
Signaling Lymphocytic Activation Molecule Family Member 1	4.57
**Cytotoxic T-lymphocyte**	**24.32**
HPS1 Biogenesis Of Lysosomal Organelles Complex 3 Subunit 1	3.80
Mitogen-Activated Protein Kinase Kinase 3	5.66
Nucleoporin 210	5.72
RB Transcriptional Corepressor Like 2	4.47
Signaling Lymphocytic Activation Molecule Family Member 1	4.66
**Kidney**	**24.46**
Mitogen-Activated Protein Kinase Kinase 3	4.00
Nucleoporin 210	3.89
Pleckstrin Homology Domain Containing A7	3.83
RB Transcriptional Corepressor Like 2	3.64
Solute Carrier Family 12 Member 4	4.06
Solute Carrier Family 22 Member 6	5.03
**Myometrium**	**25.05**
HPS1 Biogenesis Of Lysosomal Organelles Complex 3 Subunit 1	4.53
Mitogen-Activated Protein Kinase Kinase 3	4.07
RB Transcriptional Corepressor Like 2	3.11
Ring Finger Protein 10	3.69
Solute Carrier Family 12 Member 4	3.79
Solute Carrier Family 25 Member 27	5.85
**Retina**	**25.10**
Mitogen-Activated Protein Kinase Kinase 3	4.79
Nucleoporin 210	4.99
Pleckstrin Homology Domain Containing A7	4.27
Solute Carrier Family 12 Member 4	3.17
Solute Carrier Family 25 Member 27	4.17
Zinc Finger Protein 462	3.70
**Lymph node**	**25.12**
HPS1 Biogenesis Of Lysosomal Organelles Complex 3 Subunit 1	2.76
Mitogen-Activated Protein Kinase Kinase 3	5.21
Nucleoporin 210	5.09
RB Transcriptional Corepressor Like 2	4.04
Signaling Lymphocytic Activation Molecule Family Member 1	4.53
Solute Carrier Family 12 Member 4	3.49
**Esophagus**	**27.07**
Mitogen-Activated Protein Kinase Kinase 3	4.47
Mucin 5AC, Oligomeric Mucus/Gel-Forming	2.89
Nucleoporin 210	3.93
Pleckstrin Homology Domain Containing A7	2.84
RB Transcriptional Corepressor Like 2	4.78
Signaling Lymphocytic Activation Molecule Family Member 1	3.38
Solute Carrier Family 12 Member 4	4.79
**Liver**	**27.72**
HPS1 Biogenesis Of Lysosomal Organelles Complex 3 Subunit 1	3.66
Mitogen-Activated Protein Kinase Kinase 3	4.67
Nucleoporin 210	4.52
Pleckstrin Homology Domain Containing A7	4.28
RB Transcriptional Corepressor Like 2	3.66
Ring Finger Protein 10	3.12
Solute Carrier Family 12 Member 4	3.81
**Salivary Gland**	**30.07**
Mitogen-Activated Protein Kinase Kinase 3	5.17
Mucin 5AC, Oligomeric Mucus/Gel-Forming	2.56
Nucleoporin 210	4.00
Pleckstrin Homology Domain Containing A7	4.48
Presenilin 2	3.15
RB Transcriptional Corepressor Like 2	3.38
Solute Carrier Family 12 Member 4	4.17
Zinc Finger Protein 462	3.17
**Spleen**	**31.04**
Mitogen-Activated Protein Kinase Kinase 3	4.91
Mucin 5AC, Oligomeric Mucus/Gel-Forming	2.14
Nucleoporin 210	4.15
Pleckstrin Homology Domain Containing A7	2.72
Presenilin 2	3.78
RB Transcriptional Corepressor Like 2	4.72
Signaling Lymphocytic Activation Molecule Family Member 1	4.41
Solute Carrier Family 12 Member 4	4.19
**Adrenal Gland**	**32.13**
HPS1 Biogenesis Of Lysosomal Organelles Complex 3 Subunit 1	3.69
Mitogen-Activated Protein Kinase Kinase 3	4.85
Nucleoporin 210	4.26
Pleckstrin Homology Domain Containing A7	3.41
Presenilin 2	3.96
RB Transcriptional Corepressor Like 2	3.86
Solute Carrier Family 12 Member 4	4.82
Zinc Finger Protein 462	3.28
**Thyroid Gland**	**32.46**
Beta-1,3-Galactosyltransferase 5	3.50
HPS1 Biogenesis Of Lysosomal Organelles Complex 3 Subunit 1	3.90
Mitogen-Activated Protein Kinase Kinase 3	4.65
Mucin 5AC, Oligomeric Mucus/Gel-Forming	2.53
Nucleoporin 210	4.68
Pleckstrin Homology Domain Containing A7	4.45
RB Transcriptional Corepressor Like 2	3.89
Solute Carrier Family 12 Member 4	4.85
**Placenta**	**32.78**
HPS1 Biogenesis Of Lysosomal Organelles Complex 3 Subunit 1	3.94
Mitogen-Activated Protein Kinase Kinase 3	5.03
Nucleoporin 210	4.48
Pleckstrin Homology Domain Containing A7	4.47
RB Transcriptional Corepressor Like 2	3.52
Ring Finger Protein 10	3.46
Solute Carrier Family 12 Member 4	5.26
Zinc Finger Protein 462	2.63
**Prostate Gland**	**33.04**
HPS1 Biogenesis Of Lysosomal Organelles Complex 3 Subunit 1	4.16
Mitogen-Activated Protein Kinase Kinase 3	4.49
Nucleoporin 210	4.40
Pleckstrin Homology Domain Containing A7	4.37
Presenilin 2	4.24
RB Transcriptional Corepressor Like 2	3.92
Solute Carrier Family 12 Member 4	4.48
Zinc Finger Protein 462	2.99
**Testis**	**33.68**
HPS1 Biogenesis Of Lysosomal Organelles Complex 3 Subunit 1	3.25
Mitogen-Activated Protein Kinase Kinase 3	4.51
Mucin 5AC, Oligomeric Mucus/Gel-Forming	2.54
Nucleoporin 210	4.79
Pleckstrin Homology Domain Containing A7	3.37
Presenilin 2	4.11
RB Transcriptional Corepressor Like 2	3.57
Solute Carrier Family 12 Member 4	4.19
Zinc Finger Protein 462	3.35
**Stomach**	**33.79**
Beta-1,3-Galactosyltransferase 5	3.44
Mitogen-Activated Protein Kinase Kinase 3	5.02
Mucin 5AC, Oligomeric Mucus/Gel-Forming	6.17
Nucleoporin 210	4.05
Pleckstrin Homology Domain Containing A7	4.06
RB Transcriptional Corepressor Like 2	3.11
Signaling Lymphocytic Activation Molecule Family Member 1	3.85
Solute Carrier Family 12 Member 4	4.09
**Brain**	**37.57**
Mitogen-Activated Protein Kinase Kinase 3	3.85
Nucleoporin 210	4.39
Pleckstrin Homology Domain Containing A7	4.38
Presenilin 2	3.24
RB Transcriptional Corepressor Like 2	4.59
Rho Guanine Nucleotide Exchange Factor 4	4.82
Solute Carrier Family 12 Member 4	4.88
Solute Carrier Family 25 Member 27	4.11
Zinc Finger Protein 462	3.31
**Rectum**	**37.68**
Beta-1,3-Galactosyltransferase 5	4.68
Mitogen-Activated Protein Kinase Kinase 3	4.83
Mucin 5AC, Oligomeric Mucus/Gel-Forming	3.33
Nucleoporin 210	4.83
Pleckstrin Homology Domain Containing A7	3.69
RB Transcriptional Corepressor Like 2	4.75
Solute Carrier Family 12 Member 4	3.74
Solute Carrier Family 25 Member 27	5.41
Zinc Finger Protein 462	2.42
**Breast Cancer Cell**	**37.87**
Mitogen-Activated Protein Kinase Kinase 3	4.93
Mucin 5AC, Oligomeric Mucus/Gel-Forming	5.99
Nucleoporin 210	5.44
Pleckstrin Homology Domain Containing A7	3.46
Presenilin 2	3.46
RB Transcriptional Corepressor Like 2	3.27
Solute Carrier Family 12 Member 4	4.12
Solute Carrier Family 22 Member 6	4.15
Zinc Finger Protein 462	3.04
**Colon**	**39.21**
Beta-1,3-Galactosyltransferase 5	4.41
Mitogen-Activated Protein Kinase Kinase 3	4.96
Mucin 5AC, Oligomeric Mucus/Gel-Forming	4.38
Nucleoporin 210	4.74
Pleckstrin Homology Domain Containing A7	4.87
RB Transcriptional Corepressor Like 2	4.10
Solute Carrier Family 12 Member 4	3.99
Solute Carrier Family 25 Member 27	4.90
Zinc Finger Protein 462	2.87
**Tonsil**	**39.26**
HPS1 Biogenesis Of Lysosomal Organelles Complex 3 Subunit 1	3.07
Mitogen-Activated Protein Kinase Kinase 3	5.31
Nucleoporin 210	4.11
Pleckstrin Homology Domain Containing A7	4.69
RB Transcriptional Corepressor Like 2	4.31
Rho Guanine Nucleotide Exchange Factor 4	2.74
Ring Finger Protein 10	2.58
Signaling Lymphocytic Activation Molecule Family Member 1	4.52
Solute Carrier Family 12 Member 4	3.92
Zinc Finger Protein 462	4.01
**Pancreas**	**39.33**
Beta-1,3-Galactosyltransferase 5	3.69
HPS1 Biogenesis Of Lysosomal Organelles Complex 3 Subunit 1	3.31
Mitogen-Activated Protein Kinase Kinase 3	4.64
Mucin 5AC, Oligomeric Mucus/Gel-Forming	3.80
Nucleoporin 210	3.84
Pleckstrin Homology Domain Containing A7	4.94
Presenilin 2	3.86
RB Transcriptional Corepressor Like 2	3.44
Rho Guanine Nucleotide Exchange Factor 4	3.39
Solute Carrier Family 12 Member 4	4.43
**Gall Bladder**	**39.61**
Beta-1,3-Galactosyltransferase 5	4.49
HPS1 Biogenesis Of Lysosomal Organelles Complex 3 Subunit 1	3.52
Mitogen-Activated Protein Kinase Kinase 3	5.08
Mucin 5AC, Oligomeric Mucus/Gel-Forming	5.15
Nucleoporin 210	4.40
Pleckstrin Homology Domain Containing A7	4.25
Presenilin 2	3.38
RB Transcriptional Corepressor Like 2	4.87
Solute Carrier Family 12 Member 4	4.46
**Lung**	**39.90**
HPS1 Biogenesis Of Lysosomal Organelles Complex 3 Subunit 1	4.18
Mitogen-Activated Protein Kinase Kinase 3	4.48
Mucin 5AC, Oligomeric Mucus/Gel-Forming	3.98
Nucleoporin 210	3.91
Pleckstrin Homology Domain Containing A7	3.53
Presenilin 2	3.85
RB Transcriptional Corepressor Like 2	4.39
Signaling Lymphocytic Activation Molecule Family Member 1	4.04
Solute Carrier Family 12 Member 4	3.71
Zinc Finger Protein 462	3.84
**General Sum**	**1060.88**
