# Supplementary material for: Shared 6mer Peptides of Human and Omicron (21K and 21L) at SARS-CoV-2 Mutation Sites
Source: Antibodies (Basel). 2022 Oct 25;11(4):68. doi: 10.3390/antib11040068 (PMC9680445; doi:10.3390/antib11040068)
Supplement: Supplementary file 1 [file antibodies-11-00068-s001.zip › Descriptions-and-authors-note.pdf]

Descriptions of the supplementary file-contents are given below.

Figure S1:
Relevant mutations displayed at covariants.org

Document S1:
Blastp search input sequences involving mutations specific for Omicron 21K and mutations common to both Omicron 21K and Omicron 21L, along with the respective SARS-CoV-2 sequences

Document S2:
Blastp search input sequences involving mutations specific for Omicron 21L, along with the respective SARS-CoV-2 sequences

Document S3:
Alignment output of Blastp search with the input sequences involving mutations specific for Omicron 21K and mutations common to both Omicron 21K and Omicron 21L

Document S4:
Alignment output of Blastp search with the input sequences involving mutations specific for Omicron 21L

Document S5:
NetCTLpan HLA prediction-results of 364 human sequences sharing 6mers with sequences involving Omicron 21K-specific mutations and with sequences involving mutations common to both Omicron 21K and Omicron 21L (*Last 1-peptide prediction was performed after the initial 363-peptides prediction.*)

Document S6:
NetMHCcons HLA prediction-results of 364 human sequences sharing 6mers with sequences involving Omicron 21K-specific mutations and with sequences involving mutations common to both Omicron 21K and Omicron 21L (*Last 1-peptide prediction was performed after the initial 363-peptides prediction.*)

*Document S7:

NetCTLpan HLA prediction-results of 242 human sequences sharing 6mers with sequences involving Omicron 21L-specific mutations (*Last 9-peptide prediction was performed after the 233-peptides prediction results.*)

*Document S8:

NetMHCcons HLA prediction-results of 242 human sequences sharing 6mers with sequences involving Omicron 21L-specific mutations (*Last 9-peptide prediction was performed after the 233-peptides prediction results.*)

Document S9:

Source organisms of the initially predicted 363 sequences in documents S5 and S6. (*Includes deleted results after predictions indicated with a stroke-through the content at the respective lines.*)

*Document S10:

Source organisms of the initially predicted 233 sequences in documents S7 and S8. (*Includes corrected names after predictions, at ID#217–219.*)

Document S11:

NetCTLpan HLA prediction-results of 333 sequences involving Omicron 21K-specific mutations and sequences involving mutations common to both Omicron 21K and Omicron 21L. (*o at the Sequence Name indicates Omicron and q indicates SARS-CoV-2.*)

Document S12:

NetMHCcons HLA prediction-results of 333 sequences involving Omicron 21K-specific mutations and sequences involving mutations common to both Omicron 21K and Omicron 21L. (*o at the ID indicates Omicron and q indicates SARS-CoV-2.*)

Document S13:

NetCTLpan HLA prediction-results of 206 sequences involving Omicron 21L-specific mutations. (*o at the Sequence Name indicates Omicron and q indicates SARS-CoV-2.*)

Document S14:

NetMHCcons HLA prediction-results of 206 sequences involving Omicron 21L-specific mutations. (*o at the ID indicates Omicron and q indicates SARS-CoV-2.*)

Authors' note:

Orf1ab replaced the Orf1a and Orf1b expressions in the original files.

* Predictions could be done with at least 8mers, not with 7mers. Therefore, predictions with the Sequence Name (or IDs) 6, 48, 51, 120, 123, 189, and 192, are missing from documents S7 and S8 because 7mers were generated there, during Excel-processing. An amino acid (aa) was missing from the upstream or downstream of the matching 6 aa sequences, which were present towards the very end of the respective short human sequences. Accordingly, the related list-document S10 is also missing the same ID#s.
