# Supplementary material for: Shared 6mer Peptides of Human and Omicron (21K and 21L) at SARS-CoV-2 Mutation Sites
Source: Antibodies (Basel). 2022 Oct 25;11(4):68. doi: 10.3390/antib11040068 (PMC9680445; doi:10.3390/antib11040068)
Supplement: Supplementary file 1 [file antibodies-11-00068-s001.zip › DocumentS1.pdf]

Blastp search input sequences involving mutations specific for Omicron 21K and mutations common to both Omicron 21K and Omicron 21L

>S.2.pp1-YP_009724390.1

VTWFHA

>S.2.pp1-Omicron21K-A67V-delH69-delV70

VTWFHV

>S.2.pp2-YP_009724390.1

TWFHAI

>S.2.pp2-Omicron21K-A67V-delH69-delV70

TWFHVI

>S.2.pp3.1-YP_009724390.1

WFHAIH

>S.2.pp3.2-YP_009724390.1

FHAIHV

>S.2.pp3.3-YP_009724390.1

HAIHVS

>S.2.pp3-Omicron21K-A67V-delH69-delV70

WFHVIS

>S.2.pp4.1-YP_009724390.1

FHAIHV

>S.2.pp4.2-YP_009724390.1

HAIHVS

>S.2.pp4.3-YP_009724390.1

AIHVSG

>S.2.pp4-Omicron21K-A67V-delH69-delV70

FHVISG

>S.2.pp5.1-YP_009724390.1

HAIHVS

>S.2.pp5.2-YP_009724390.1

AIHVSG

>S.2.pp5.3-YP_009724390.1

IHVSGT

>S.2.pp5-Omicron21K-A67V-delH69-delV70

HVISGT

>2S.2.pp1-YP_009724390.1

TWFHAI

>2S.2.pp1-Omicron21K-A67V-delH69-delV70

TWFHVI

>2S.2.pp2.1-YP_009724390.1

WFHAIH

>2S.2.pp2.2-YP_009724390.1

FHAIHV

>2S.2.pp2.3-YP_009724390.1

HAIHVS

>2S.2.pp2-Omicron21K-A67V-delH69-delV70

WFHVIS

>2S.2.pp3.1-YP_009724390.1

FHAIHV

>2S.2.pp3.2-YP_009724390.1

HAIHVS

>2S.2.pp3.3-YP_009724390.1

AIHVSG

>2S.2.pp3-Omicron21K-A67V-delH69-delV70

FHVISG

>2S.2.pp4.1-YP_009724390.1

HAIHVS

>2S.2.pp4.2-YP_009724390.1

AIHVSG

>2S.2.pp4.3-YP_009724390.1

IHVSGT

>2S.2.pp4-Omicron21K-A67V-delH69-delV70

HVISGT

>2S.2.pp5.1-YP_009724390.1

AIHVSG

>2S.2.pp5.2-YP_009724390.1

IHVSGT

>2S.2.pp5.3-YP_009724390.1

HVSGTN

>2S.2.pp5-Omicron21K-A67V-delH69-delV70

VISGTN

>S.3.pp1-YP_009724390.1

VYFAST

>S.3.pp1-Omicron21K-T95I

VYFASI

>S.3.pp2-YP_009724390.1

YFASTE

>S.3.pp2-Omicron21K-T95I

YFASIE

>S.3.pp3-YP_009724390.1

FASTEK

>S.3.pp3-Omicron21K-T95I

FASIEK

>S.3.pp4-YP_009724390.1

ASTEKS

>S.3.pp4-Omicron21K-T95I

ASIEKS

>S.3.pp5-YP_009724390.1

STEKSN

>S.3.pp5-Omicron21K-T95I

SIEKSN

>2S.3.pp1-YP_009724390.1

YFASTE

>2S.3.pp1-Omicron21K-T95I

YFASIE

>2S.3.pp2-YP_009724390.1

FASTEK

>2S.3.pp2-Omicron21K-T95I

FASIEK

>2S.3.pp3-YP_009724390.1

ASTEKS

>2S.3.pp3-Omicron21K-T95I

ASIEKS

>2S.3.pp4-YP_009724390.1

STEKSN

>2S.3.pp4-Omicron21K-T95I

SIEKSN

>2S.3.pp5-YP_009724390.1

TEKSNI

>2S.3.pp5-Omicron21K-T95I

IEKSNI

>S.4.pp1-YP_009724390.1

NDPFLG

>S.4.1.pp1-Omicron21K-G142D-delV143-delY144-delY145

NDPFLD

>S.4.pp2.1-YP_009724390.1

DPFLGV

>S.4.pp2.2-YP_009724390.1

PFLGVY

>S.4.pp2.3-YP_009724390.1

FLGVYY

>S.4.pp2.4-YP_009724390.1

LGVYYH

>S.4.1.pp2-Omicron21K-G142D-delV143-delY144-delY145

DPFLDH

>S.4.pp3.1-YP_009724390.1

PFLGVY

>S.4.pp3.2-YP_009724390.1

FLGVYY

>S.4.pp3.3-YP_009724390.1

LGVYYH

>S.4.pp3.4-YP_009724390.1

GVYYHK

>S.4.1.pp3-Omicron21K-G142D-delV143-delY144-delY145

PFLDHK

>S.4.pp4.1-YP_009724390.1

FLGVYY

>S.4.pp4.2-YP_009724390.1

LGVYYH

>S.4.pp4.3-YP_009724390.1

GVYYHK

>S.4.pp4.4-YP_009724390.1

VYYHKN

>S.4.1.pp4-Omicron21K-G142D-delV143-delY144-delY145

FLDHKN

>S.4.pp5.1-YP_009724390.1

LGVYYH

>S.4.pp5.2-YP_009724390.1

GVYYHK

>S.4.pp5.3-YP_009724390.1

VYYHKN

>S.4.pp5.4-YP_009724390.1

YYHKNN

>S.4.1.pp5-Omicron21K-G142D-delV143-delY144-delY145

LDHKNN

>2S.4.pp1.1-YP_009724390.1

DPFLGV

>2S.4.pp1.2-YP_009724390.1

PFLGVY

>2S.4.pp1.3-YP_009724390.1

FLGVYY

>2S.4.pp1.4-YP_009724390.1

LGVYYH

>2S.4.1.pp1-Omicron21K-G142D-delV143-delY144-delY145

DPFLDH

>2S.4.pp2.1-YP_009724390.1

PFLGVY

>2S.4.pp2.2-YP_009724390.1

FLGVYY

>2S.4.pp2.3-YP_009724390.1

LGVYYH

>2S.4.pp2.4-YP_009724390.1

GVYYHK

>2S.4.1.pp2-Omicron21K-G142D-delV143-delY144-delY145

PFLDHK

>2S.4.pp3.1-YP_009724390.1

FLGVYY

>2S.4.pp3.2-YP_009724390.1

LGVYYH

>2S.4.pp3.3-YP_009724390.1

GVYYHK

>2S.4.pp3.4-YP_009724390.1

VYYHKN

>2S.4.1.pp3-Omicron21K-G142D-delV143-delY144-delY145

FLDHKN

>2S.4.pp4.1-YP_009724390.1

LGVYYH

>2S.4.pp4.2-YP_009724390.1

GVYYHK

>2S.4.pp4.3-YP_009724390.1

VYYHKN

>2S.4.pp4.4-YP_009724390.1

YYHKNN

>2S.4.1.pp4-Omicron21K-G142D-delV143-delY144-delY145

LDHKNN

>2S.4.pp5.1-YP_009724390.1

GVYYHK

>2S.4.pp5.2-YP_009724390.1

VYYHKN

>2S.4.pp5.3-YP_009724390.1

YYHKNN

>2S.4.pp5.4-YP_009724390.1

YHKNNK

>2S.4.1.pp5-Omicron21K-G142D-delV143-delY144-delY145

DHKNNK

>S.5.pp1.1-YP_009724390.1

KHTPIN

>S.5.pp1.2-pp2.1-YP_009724390.1

HTPINL

>S.5.pp2.2-pp3.1-YP_009724390.1

TPINLV

>S.5.pp3.2-pp4-YP_009724390.1

PINLVR

>S.5.pp5-YP_009724390.1

INLVRD

>S.5.pp6-YP_009724390.1

NLVRDL

>S.5.pp7-YP_009724390.1

LVRDLP

>S.5.pp8-YP_009724390.1

VRDLPQ

>S.5.1.pp1-Omicron21K-delN211-L212I-ins214EPE

KHTPII

>S.5.1.pp2-Omicron21K-delN211-L212I-ins214EPE

HTPIIV

>S.5.1.pp3-Omicron21K-delN211-L212I-ins214EPE

TPIIVR

>S.5.1.pp4.1-Omicron21K-delN211-L212I-ins214EPE

PIIVRE

>S.5.1.pp4.2-pp5.1-pp6.1-Omicron21K-delN211-L212I-ins214EPE

IIVREP

>S.5.1.pp4.3-pp5.2-pp6.2-pp7.1-Omicron21K-delN211-L212I-ins214EPE

IVREPE

>S.5.1.pp5.3-pp6.3-pp7.2-pp8.1-Omicron21K-delN211-L212I-ins214EPE

VREPED

>S.5.1.pp6.4-pp7.3-pp8.2-Omicron21K-delN211-L212I-ins214EPE

REPEDL

>S.5.1.pp7.4-pp8.3-Omicron21K-delN211-L212I-ins214EPE

EPEDLP

>S.5.1.pp8.4-Omicron21K-delN211-L212I-ins214EPE

PEDLPQ

>2S.5.pp1.1-YP_009724390.1

HTPINL

>2S.5.pp1.2-pp2.1-YP_009724390.1

TPINLV

>2S.5.pp2.2-pp3.1-YP_009724390.1

PINLVR

>2S.5.pp3.2-pp4-YP_009724390.1

INLVRD

>2S.5.pp5-YP_009724390.1

NLVRDL

>2S.5.pp6-YP_009724390.1

LVRDLP

>2S.5.pp7-YP_009724390.1

VRDLPQ

>2S.5.pp8-YP_009724390.1

RDLPQG

>2S.5.1.pp1-Omicron21K-delN211-L212I-ins214EPE

HTPIIV

>2S.5.1.pp2-Omicron21K-delN211-L212I-ins214EPE

TPIIVR

>2S.5.1.pp3-Omicron21K-delN211-L212I-ins214EPE

PIIVRE

>2S.5.1.pp4.1-Omicron21K-delN211-L212I-ins214EPE

IIVREP

>2S.5.1.pp4.2-pp5.1-pp6.1-Omicron21K-delN211-L212I-ins214EPE

IVREPE

>2S.5.1.pp4.3-pp5.2-pp6.2-pp7.1-Omicron21K-delN211-L212I-ins214EPE

VREPED

>2S.5.1.pp5.3-pp6.3-pp7.2-pp8.1-Omicron21K-delN211-L212I-ins214EPE

REPEDL

>2S.5.1.pp6.4-pp7.3-pp8.2-Omicron21K-delN211-L212I-ins214EPE

EPEDLP

>2S.5.1.pp7.4-pp8.3-Omicron21K-delN211-L212I-ins214EPE

PEDLPQ

>2S.5.1.pp8.4-Omicron21K-delN211-L212I-ins214EPE

EDLPQG

>S.6.pp1-YP_009724390.1

NLCPFG

>S.6.pp1-Omicron21K-G339D-21L-G339D

NLCPFD

>S.6.pp2-YP_009724390.1

LCPFGE

>S.6.pp2-Omicron21K-G339D-21L-G339D

LCPFDE

>S.6.pp3-YP_009724390.1

CPFGEV

>S.6.pp3-Omicron21K-G339D-21L-G339D

CPFDEV

>S.6.pp4-YP_009724390.1

PFGEVF

>S.6.pp4-Omicron21K-G339D-21L-G339D

PFDEVF

>S.6.pp5-YP_009724390.1

FGEVFN

>S.6.pp5-Omicron21K-G339D-21L-G339D

FDEVFN

>2S.6.pp1-YP_009724390.1

LCPFGE

>2S.6.pp1-Omicron21K-G339D-21L-G339D

LCPFDE

>2S.6.pp2-YP_009724390.1

CPFGEV

>2S.6.pp2-Omicron21K-G339D-21L-G339D

CPFDEV

>2S.6.pp3-YP_009724390.1

PFGEVF

>2S.6.pp3-Omicron21K-G339D-21L-G339D

PFDEVF

>2S.6.pp4-YP_009724390.1

FGEVFN

>2S.6.pp4-Omicron21K-G339D-21L-G339D

FDEVFN

>2S.6.pp5-YP_009724390.1

GEVFNA

>2S.6.pp5-Omicron21K-G339D-21L-G339D

DEVFNA

>S.7.pp1-YP_009724390.1

SVLYNS

>S.7.1.pp1-Omicron21K-S371L-S373P-S375F

SVLYNL

>S.7.pp2-YP_009724390.1

VLYNSA

>S.7.1.pp2-Omicron21K-S371L-S373P-S375F

VLYNLA

>S.7.pp3-YP_009724390.1

LYNSAS

>S.7.1.pp3-Omicron21K-S371L-S373P-S375F

LYNLAP

>S.7.pp4-YP_009724390.1

YNSASF

>S.7.1.pp4-Omicron21K-S371L-S373P-S375F

YNLAPF

>S.7.pp5-YP_009724390.1

NSASFS

>S.7.1.pp5-Omicron21K-S371L-S373P-S375F

NLAPFF

>S.7.pp6-YP_009724390.1

SASFST

>S.7.1.pp6-Omicron21K-S371L-S373P-S375F

LAPFFT

>S.7.pp7-YP_009724390.1

ASFSTF

>S.7.1.pp7-Omicron21K-S371L-S373P-S375F

APFFTF

>S.7.pp8-YP_009724390.1

SFSTFK

>S.7.1.pp8-Omicron21K-S371L-S373P-S375F

PFFTFK

>S.7.pp9-YP_009724390.1

FSTFKC

>S.7.1.pp9-Omicron21K-S371L-S373P-S375F

FFTFKC

>2S.7.pp1-YP_009724390.1

VLYNSA

>2S.7.1.pp1-Omicron21K-S371L-S373P-S375F

VLYNLA

>2S.7.pp2-YP_009724390.1

LYNSAS

>2S.7.1.pp2-Omicron21K-S371L-S373P-S375F

LYNLAP

>2S.7.pp3-YP_009724390.1

YNSASF

>2S.7.1.pp3-Omicron21K-S371L-S373P-S375F

YNLAPF

>2S.7.pp4-YP_009724390.1

NSASFS

>2S.7.1.pp4-Omicron21K-S371L-S373P-S375F

NLAPFF

>2S.7.pp5-YP_009724390.1

SASFST

>2S.7.1.pp5-Omicron21K-S371L-S373P-S375F

LAPFFT

>2S.7.pp6-YP_009724390.1

ASFSTF

>2S.7.1.pp6-Omicron21K-S371L-S373P-S375F

APFFTF

>2S.7.pp7-YP_009724390.1

SFSTFK

>2S.7.1.pp7-Omicron21K-S371L-S373P-S375F

PFFTFK

>2S.7.pp8-YP_009724390.1

FSTFKC

>2S.7.1.pp8-Omicron21K-S371L-S373P-S375F

FFTFKC

>2S.7.pp9-YP_009724390.1

STFKCY

>2S.7.1.pp9-Omicron21K-S371L-S373P-S375F

FTFKCY

>S.9.pp1-YP_009724390.1

PGQTGK

>S.9.pp1-Omicron21K-K417N-21L-K417N

PGQTGN

>S.9.pp2-YP_009724390.1

GQTGKI

>S.9.pp2-Omicron21K-K417N-21L-K417N

GQTGNI

>S.9.pp3-YP_009724390.1

QTGKIA

>S.9.pp3-Omicron21K-K417N-21L-K417N

QTGNIA

>S.9.pp4-YP_009724390.1

TGKIAD

>S.9.pp4-Omicron21K-K417N-21L-K417N

TGNIAD

>S.9.pp5-YP_009724390.1

GKIADY

>S.9.pp5-Omicron21K-K417N-21L-K417N

GNIADY

>2S.9.pp1-YP_009724390.1

GQTGKI

>2S.9.pp1-Omicron21K-K417N-21L-K417N

GQTGNI

>2S.9.pp2-YP_009724390.1

QTGKIA

>2S.9.pp2-Omicron21K-K417N-21L-K417N

QTGNIA

>2S.9.pp3-YP_009724390.1

TGKIAD

>2S.9.pp3-Omicron21K-K417N-21L-K417N

TGNIAD

>2S.9.pp4-YP_009724390.1

GKIADY

>2S.9.pp4-Omicron21K-K417N-21L-K417N

GNIADY

>2S.9.pp5-YP_009724390.1

KIADYN

>2S.9.pp5-Omicron21K-K417N-21L-K417N

NIADYN

>S.10.p1.pp1-YP_009724390.1

AWNSNN

>S.10.1.p1.pp1-Omicron21K-N440K-21L-N440K

AWNSNK

>S.10.p1.pp2-YP_009724390.1

WNSNNL

>S.10.1.p1.pp2-Omicron21K-N440K-21L-N440K

WNSNKL

>S.10.p1.pp3-YP_009724390.1

NSNNLD

>S.10.1.p1.pp3-Omicron21K-N440K-21L-N440K

NSNKLD

>S.10.p1.pp4-YP_009724390.1

SNNLDS

>S.10.1.p1.pp4-Omicron21K-N440K-21L-N440K

SNKLDS

>S.10.p1.pp5-YP_009724390.1

NNLDSK

>S.10.1.p1.pp5-Omicron21K-N440K-21L-N440K

NKLDSK

>2S.10.p1.pp1-YP_009724390.1

WNSNNL

>2S.10.1.p1.pp1-Omicron21K-N440K-21L-N440K

WNSNKL

>2S.10.p1.pp2-YP_009724390.1

NSNNLD

>2S.10.1.p1.pp2-Omicron21K-N440K-21L-N440K

NSNKLD

>2S.10.p1.pp3-YP_009724390.1

SNNLDS

>2S.10.1.p1.pp3-Omicron21K-N440K-21L-N440K

SNKLDS

>2S.10.p1.pp4-YP_009724390.1

NNLDSK

>2S.10.1.p1.pp4-Omicron21K-N440K-21L-N440K

NKLDSK

>2S.10.p1.pp5-YP_009724390.1

NLDSKV

>2S.10.1.p1.pp5-Omicron21K-N440K-21L-N440K

KLDSKV

>S.10.p2.pp1-YP_009724390.1

LDSKVG

>S.10.1.p2.pp1-Omicron21K-G446S

LDSKVS

>S.10.p2.pp2-YP_009724390.1

DSKVGG

>S.10.1.p2.pp2-Omicron21K-G446S

DSKVSG

>S.10.p2.pp3-YP_009724390.1

SKVGGN

>S.10.1.p2.pp3-Omicron21K-G446S

SKVSGN

>S.10.p2.pp4-YP_009724390.1

KVGGNY

>S.10.1.p2.pp4-Omicron21K-G446S

KVSGNY

>S.10.p2.pp5-YP_009724390.1

VGGNYN

>S.10.1.p2.pp5-Omicron21K-G446S

VSGNYN

>2S.10.p2.pp1-YP_009724390.1

DSKVGG

>2S.10.1.p2.pp1-Omicron21K-G446S

DSKVSG

>2S.10.p2.pp2-YP_009724390.1

SKVGGN

>2S.10.1.p2.pp2-Omicron21K-G446S

SKVSGN

>2S.10.p2.pp3-YP_009724390.1

KVGGNY

>2S.10.1.p2.pp3-Omicron21K-G446S

KVSGNY

>2S.10.p2.pp4-YP_009724390.1

VGGNYN

>2S.10.1.p2.pp4-Omicron21K-G446S

VSGNYN

>2S.10.p2.pp5-YP_009724390.1

GGNYNY

>2S.10.1.p2.pp5-Omicron21K-G446S

SGNYNY

>S.11.p1.pp1-YP_009724390.1

IYQAGS

>S.11.p1.pp1-Omicron21K-S477N-T478K-E484A-21L-S477N-T478K-E484A

IYQAGN

>S.11.p1.pp2-YP_009724390.1

YQAGST

>S.11.p1.pp2-Omicron21K-S477N-T478K-E484A-21L-S477N-T478K-E484A

YQAGNK

>S.11.p1.pp3-YP_009724390.1

QAGSTP

>S.11.p1.pp3-Omicron21K-S477N-T478K-E484A-21L-S477N-T478K-E484A

QAGNKP

>S.11.p1.pp4-YP_009724390.1

AGSTPC

>S.11.p1.pp4-Omicron21K-S477N-T478K-E484A-21L-S477N-T478K-E484A

AGNKPC

>S.11.p1.pp5-YP_009724390.1

GSTPCN

>S.11.p1.pp5-Omicron21K-S477N-T478K-E484A-21L-S477N-T478K-E484A

GNKPCN

>S.11.p1.pp6-YP_009724390.1

STPCNG

>S.11.p1.pp6-Omicron21K-S477N-T478K-E484A-21L-S477N-T478K-E484A

NKPCNG

>2S.11.p1.pp1-YP_009724390.1

YQAGST

>2S.11.p1.pp1-Omicron21K-S477N-T478K-E484A-21L-S477N-T478K-E484A

YQAGNK

>2S.11.p1.pp2-YP_009724390.1

QAGSTP

>2S.11.p1.pp2-Omicron21K-S477N-T478K-E484A-21L-S477N-T478K-E484A

QAGNKP

>2S.11.p1.pp3-YP_009724390.1

AGSTPC

>2S.11.p1.pp3-Omicron21K-S477N-T478K-E484A-21L-S477N-T478K-E484A

AGNKPC

>2S.11.p1.pp4-YP_009724390.1

GSTPCN

>2S.11.p1.pp4-Omicron21K-S477N-T478K-E484A-21L-S477N-T478K-E484A

GNKPCN

>2S.11.p1.pp5-YP_009724390.1

STPCNG

>2S.11.p1.pp5-Omicron21K-S477N-T478K-E484A-21L-S477N-T478K-E484A

NKPCNG

>2S.11.p1.pp6-YP_009724390.1

TPCNGV

>2S.11.p1.pp6-Omicron21K-S477N-T478K-E484A-21L-S477N-T478K-E484A

KPCNGV

>S.11.p2.pp1-YP_009724390.1

PCNGVE

>S.11.p2.pp1-Omicron21K-S477N-T478K-E484A-21L-S477N-T478K-E484A

PCNGVA

>S.11.p2.pp2-YP_009724390.1

CNGVEG

>S.11.p2.pp2-Omicron21K-S477N-T478K-E484A-21L-S477N-T478K-E484A

CNGVAG

>S.11.p2.pp3-YP_009724390.1

NGVEGF

>S.11.p2.pp3-Omicron21K-S477N-T478K-E484A-21L-S477N-T478K-E484A

NGVAGF

>S.11.p2.pp4-YP_009724390.1

GVEGFN

>S.11.p2.pp4-Omicron21K-S477N-T478K-E484A-21L-S477N-T478K-E484A

GVAGFN

>S.11.p2.pp5-YP_009724390.1

VEGFNC

>S.11.p2.pp5-Omicron21K-S477N-T478K-E484A-21L-S477N-T478K-E484A

VAGFNC

>2S.11.p2.pp1-YP_009724390.1

CNGVEG

>2S.11.p2.pp1-Omicron21K-S477N-T478K-E484A-21L-S477N-T478K-E484A

CNGVAG

>2S.11.p2.pp2-YP_009724390.1

NGVEGF

>2S.11.p2.pp2-Omicron21K-S477N-T478K-E484A-21L-S477N-T478K-E484A

NGVAGF

>2S.11.p2.pp3-YP_009724390.1

GVEGFN

>2S.11.p2.pp3-Omicron21K-S477N-T478K-E484A-21L-S477N-T478K-E484A

GVAGFN

>2S.11.p2.pp4-YP_009724390.1

VEGFNC

>2S.11.p2.pp4-Omicron21K-S477N-T478K-E484A-21L-S477N-T478K-E484A

VAGFNC

>2S.11.p2.pp5-YP_009724390.1

EGFNCY

>2S.11.p2.pp5-Omicron21K-S477N-T478K-E484A-21L-S477N-T478K-E484A

AGFNCY

>S.12.pp1-YP_009724390.1

CYFPLQ

>S.12.1.pp1-Omicron21K-Q493R-G496S-Q498R-N501Y-Y505H-21L-Q493R-Q498R-N501Y-Y505H

CYFPLR

>S.12.pp2-YP_009724390.1

YFPLQS

>S.12.1.pp2-Omicron21K-Q493R-G496S-Q498R-N501Y-Y505H-21L-Q493R-Q498R-N501Y-Y505H

YFPLRS

>S.12.pp3-YP_009724390.1

FPLQSY

>S.12.1.pp3-Omicron21K-Q493R-G496S-Q498R-N501Y-Y505H-21L-Q493R-Q498R-N501Y-Y505H

FPLRSY

>S.12.pp4-YP_009724390.1

PLQSYG

>S.12.1.pp4-Omicron21K-Q493R-G496S-Q498R-N501Y-Y505H-21L-Q493R-Q498R-N501Y-Y505H

PLRSYS

>S.12.pp5-YP_009724390.1

LQSYGF

>S.12.1.pp5-Omicron21K-Q493R-G496S-Q498R-N501Y-Y505H-21L-Q493R-Q498R-N501Y-Y505H

LRSYSF

>S.12.pp6-YP_009724390.1

QSYGFQ

>S.12.1.pp6-Omicron21K-Q493R-G496S-Q498R-N501Y-Y505H

RSYSFR

>S.12.pp7-YP_009724390.1

SYGFQP

>S.12.1.pp7-Omicron21K-Q493R-G496S-Q498R-N501Y-Y505H

SYSFRP

>S.12.pp8-YP_009724390.1

YGFQPT

>S.12.1.pp8-Omicron21K-Q493R-G496S-Q498R-N501Y-Y505H

YSFRPT

>S.12.pp9-YP_009724390.1

GFQPTN

>S.12.1.pp9-Omicron21K-Q493R-G496S-Q498R-N501Y-Y505H

SFRPTY

>S.12.pp10-YP_009724390.1

FQPTNG

>S.12.1.pp10-Omicron21K-Q493R-G496S-Q498R-N501Y-Y505H

FRPTYG

>S.12.pp11-YP_009724390.1

QPTNGV

>S.12.1.pp11-Omicron21K-Q493R-G496S-Q498R-N501Y-Y505H-21L-Q493R-Q498R-N501Y-Y505H

RPTYGV

>S.12.pp12-YP_009724390.1

PTNGVG

>S.12.1.pp12-Omicron21K-Q493R-G496S-Q498R-N501Y-Y505H-21L-Q493R-Q498R-N501Y-Y505H

PTYGVG

>S.12.pp13-YP_009724390.1

TNGVGY

>S.12.1.pp13-Omicron21K-Q493R-G496S-Q498R-N501Y-Y505H-21L-Q493R-Q498R-N501Y-Y505H

TYGVGH

>S.12.pp14-YP_009724390.1

NGVGYQ

>S.12.1.pp14-Omicron21K-Q493R-G496S-Q498R-N501Y-Y505H-21L-Q493R-Q498R-N501Y-Y505H

YGVGHQ

>S.12.pp15-YP_009724390.1

GVGYQP

>S.12.1.pp15-Omicron21K-Q493R-G496S-Q498R-N501Y-Y505H-21L-Q493R-Q498R-N501Y-Y505H

GVGHQP

>S.12.pp16-YP_009724390.1

VGYQPY

>S.12.1.pp16-Omicron21K-Q493R-G496S-Q498R-N501Y-Y505H-21L-Q493R-Q498R-N501Y-Y505H

VGHQPY

>S.12.pp17-YP_009724390.1

GYQPYR

>S.12.1.pp17-Omicron21K-Q493R-G496S-Q498R-N501Y-Y505H-21L-Q493R-Q498R-N501Y-Y505H

GHQPYR

>2S.12.pp1-YP_009724390.1

YFPLQS

>S.12.1.pp1-Omicron21K-Q493R-G496S-Q498R-N501Y-Y505H-21L-Q493R-Q498R-N501Y-Y505H

YFPLRS

>2S.12.pp2-YP_009724390.1

FPLQSY

>2S.12.1.pp2-Omicron21K-Q493R-G496S-Q498R-N501Y-Y505H-21L-Q493R-Q498R-N501Y-Y505H

FPLRSY

>2S.12.pp3-YP_009724390.1

PLQSYG

>2S.12.1.pp3-Omicron21K-Q493R-G496S-Q498R-N501Y-Y505H-21L-Q493R-Q498R-N501Y-Y505H

PLRSYS

>2S.12.pp4-YP_009724390.1

LQSYGF

>2S.12.1.pp4-Omicron21K-Q493R-G496S-Q498R-N501Y-Y505H-21L-Q493R-Q498R-N501Y-Y505H

LRSYSF

>2S.12.pp5-YP_009724390.1

QSYGFQ

>2S.12.1.pp5-Omicron21K-Q493R-G496S-Q498R-N501Y-Y505H-21L-Q493R-Q498R-N501Y-Y505H

RSYSFR

>2S.12.pp6-YP_009724390.1

SYGFQP

>2S.12.1.pp6-Omicron21K-Q493R-G496S-Q498R-N501Y-Y505H

SYSFRP

>2S.12.pp7-YP_009724390.1

YGFQPT

>2S.12.1.pp7-Omicron21K-Q493R-G496S-Q498R-N501Y-Y505H

YSFRPT

>2S.12.pp8-YP_009724390.1

GFQPTN

>2S.12.1.pp8-Omicron21K-Q493R-G496S-Q498R-N501Y-Y505H

SFRPTY

>2S.12.pp9-YP_009724390.1

FQPTNG

>2S.12.1.pp9-Omicron21K-Q493R-G496S-Q498R-N501Y-Y505H

FRPTYG

>2S.12.pp10-YP_009724390.1

QPTNGV

>2S.12.1.pp10-Omicron21K-Q493R-G496S-Q498R-N501Y-Y505H

RPTYGV

>2S.12.pp11-YP_009724390.1

PTNGVG

>2S.12.1.pp11-Omicron21K-Q493R-G496S-Q498R-N501Y-Y505H-21L-Q493R-Q498R-N501Y-Y505H

PTYGVG

>2S.12.pp12-YP_009724390.1

TNGVGY

>2S.12.1.pp12-Omicron21K-Q493R-G496S-Q498R-N501Y-Y505H-21L-Q493R-Q498R-N501Y-Y505H

TYGVGH

>2S.12.pp13-YP_009724390.1

NGVGYQ

>2S.12.1.pp13-Omicron21K-Q493R-G496S-Q498R-N501Y-Y505H-21L-Q493R-Q498R-N501Y-Y505H

YGVGHQ

>2S.12.pp14-YP_009724390.1

GVGYQP

>2S.12.1.pp14-Omicron21K-Q493R-G496S-Q498R-N501Y-Y505H-21L-Q493R-Q498R-N501Y-Y505H

GVGHQP

>2S.12.pp15-YP_009724390.1

VGYQPY

>2S.12.1.pp15-Omicron21K-Q493R-G496S-Q498R-N501Y-Y505H-21L-Q493R-Q498R-N501Y-Y505H

VGHQPY

>2S.12.pp16-YP_009724390.1

GYQPYR

>2S.12.1.pp16-Omicron21K-Q493R-G496S-Q498R-N501Y-Y505H-21L-Q493R-Q498R-N501Y-Y505H

GHQPYR

>2S.12.pp17-YP_009724390.1

YQPYRV

>2S.12.1.pp17-Omicron21K-Q493R-G496S-Q498R-N501Y-Y505H-21L-Q493R-Q498R-N501Y-Y505H

HQPYRV

>S.13.pp1-YP_009724390.1

NFNGLT

>S.13.pp1-Omicron21K-T547K

NFNGLK

>S.13.pp2-YP_009724390.1

FNGLTG

>S.13.pp2-Omicron21K-T547K

FNGLKG

>S.13.pp3-YP_009724390.1

NGLTGT

>S.13.pp3-Omicron21K-T547K

NGLKGT

>S.13.pp4-YP_009724390.1

GLTGTG

>S.13.pp4-Omicron21K-T547K

GLKGTG

>S.13.pp5-YP_009724390.1

LTGTGV

>S.13.pp5-Omicron21K-T547K

LKGTGV

>2S.13.pp1-YP_009724390.1

FNGLTG

>2S.13.pp1-Omicron21K-T547K

FNGLKG

>2S.13.pp2-YP_009724390.1

NGLTGT

>2S.13.pp2-Omicron21K-T547K

NGLKGT

>2S.13.pp3-YP_009724390.1

GLTGTG

>2S.13.pp3-Omicron21K-T547K

GLKGTG

>2S.13.pp4-YP_009724390.1

LTGTGV

>2S.13.pp4-Omicron21K-T547K

LKGTGV

>2S.13.pp5-YP_009724390.1

TGTGVL

>2S.13.pp5-Omicron21K-T547K

KGTGVL

>S.14.pp1-YP_009724390.1

AVLYQD

>S.14.pp1-Omicron21K-D614G-21L-D614G

AVLYQG

>S.14.pp2-YP_009724390.1

VLYQDV

>S.14.pp2-Omicron21K-D614G-21L-D614G

VLYQGV

>S.14.pp3-YP_009724390.1

LYQDVN

>S.14.pp3-Omicron21K-D614G-21L-D614G

LYQGVN

>S.14.pp4-YP_009724390.1

YQDVNC

>S.14.pp4-Omicron21K-D614G-21L-D614G

YQGVNC

>S.14.pp5-YP_009724390.1

QDVNCT

>S.14.pp5-Omicron21K-D614G-21L-D614G

QGVNCT

>2S.14.pp1-YP_009724390.1

VLYQDV

>2S.14.pp1-Omicron21K-D614G-21L-D614G

VLYQGV

>2S.14.pp2-YP_009724390.1

LYQDVN

>2S.14.pp2-Omicron21K-D614G-21L-D614G

LYQGVN

>2S.14.pp3-YP_009724390.1

YQDVNC

>2S.14.pp3-Omicron21K-D614G-21L-D614G

YQGVNC

>2S.14.pp4-YP_009724390.1

QDVNCT

>2S.14.pp4-Omicron21K-D614G-21L-D614G

QGVNCT

>2S.14.pp5-YP_009724390.1

DVNCTE

>2S.14.pp5-Omicron21K-D614G-21L-D614G

GVNCTE

>S.15.pp1-YP_009724390.1

LIGAEH

>S.15.pp1-Omicron21K-H655Y-21L-H655Y

LIGAEY

>S.15.pp2-YP_009724390.1

IGAEHV

>S.15.pp2-Omicron21K-H655Y-21L-H655Y

IGAEYV

>S.15.pp3-YP_009724390.1

GAEHVN

>S.15.pp3-Omicron21K-H655Y-21L-H655Y

GAEYVN

>S.15.pp4-YP_009724390.1

AEHVNN

>S.15.pp4-Omicron21K-H655Y-21L-H655Y

AEYVNN

>S.15.pp5-YP_009724390.1

EHVNNS

>S.15.pp5-Omicron21K-H655Y-21L-H655Y

EYVNNS

>2S.15.pp1-YP_009724390.1

IGAEHV

>2S.15.pp1-Omicron21K-H655Y-21L-H655Y

IGAEYV

>2S.15.pp2-YP_009724390.1

GAEHVN

>2S.15.pp2-Omicron21K-H655Y-21L-H655Y

GAEYVN

>2S.15.pp3-YP_009724390.1

AEHVNN

>2S.15.pp3-Omicron21K-H655Y-21L-H655Y

AEYVNN

>2S.15.pp4-YP_009724390.1

EHVNNS

>2S.15.pp4-Omicron21K-H655Y-21L-H655Y

EYVNNS

>2S.15.pp5-YP_009724390.1

HVNNSY

>2S.15.pp5-Omicron21K-H655Y-21L-H655Y

YVNNSY

>S.16.pp1-YP_009724390.1

YQTQTN

>S.16.pp1-Omicron21K-N679K-P681H-21L-N679K-P681H

YQTQTK

>S.16.pp2-YP_009724390.1

QTQTNS

>S.16.pp2-Omicron21K-N679K-P681H-21L-N679K-P681H

QTQTKS

>S.16.pp3-YP_009724390.1

TQTNSP

>S.16.pp3-Omicron21K-N679K-P681H-21L-N679K-P681H

TQTKSH

>S.16.pp4-YP_009724390.1

QTNSPR

>S.16.pp4-Omicron21K-N679K-P681H-21L-N679K-P681H

QTKSHR

>S.16.pp5-YP_009724390.1

TNSPRR

>S.16.pp5-Omicron21K-N679K-P681H-21L-N679K-P681H

TKSHRR

>S.16.pp6-YP_009724390.1

NSPRRA

>S.16.pp6-Omicron21K-N679K-P681H-21L-N679K-P681H

KSHRRA

>S.16.pp7-YP_009724390.1

SPRRAR

>S.16.pp7-Omicron21K-N679K-P681H-21L-N679K-P681H

SHRRAR

>2S.16.pp1-YP_009724390.1

QTQTNS

>2S.16.pp1-Omicron21K-N679K-P681H-21L-N679K-P681H

QTQTKS

>2S.16.pp2-YP_009724390.1

TQTNSP

>2S.16.pp2-Omicron21K-N679K-P681H-21L-N679K-P681H

TQTKSH

>2S.16.pp3-YP_009724390.1

QTNSPR

>2S.16.pp3-Omicron21K-N679K-P681H-21L-N679K-P681H

QTKSHR

>2S.16.pp4-YP_009724390.1

TNSPRR

>2S.16.pp4-Omicron21K-N679K-P681H-21L-N679K-P681H

TKSHRR

>2S.16.pp5-YP_009724390.1

NSPRRA

>2S.16.pp5-Omicron21K-N679K-P681H-21L-N679K-P681H

KSHRRA

>2S.16.pp6-YP_009724390.1

SPRRAR

>2S.16.pp6-Omicron21K-N679K-P681H-21L-N679K-P681H

SHRRAR

>2S.16.pp7-YP_009724390.1

PRRARS

>2S.16.pp7-Omicron21K-N679K-P681H-21L-N679K-P681H

HRRARS

>S.17.pp1-YP_009724390.1

FCTQLN

>S.17.pp1-Omicron21K-N764K-21L-N764K

FCTQLK

>S.17.pp2-YP_009724390.1

CTQLNR

>S.17.pp2-Omicron21K-N764K-21L-N764K

CTQLKR

>S.17.pp3-YP_009724390.1

TQLNRA

>S.17.pp3-Omicron21K-N764K-21L-N764K

TQLKRA

>S.17.pp4-YP_009724390.1

QLNRAL

>S.17.pp4-Omicron21K-N764K-21L-N764K

QLKRAL

>S.17.pp5-YP_009724390.1

LNRALT

>S.17.pp5-Omicron21K-N764K-21L-N764K

LKRALT

>2S.17.pp1-YP_009724390.1

CTQLNR

>2S.17.pp1-Omicron21K-N764K-21L-N764K

CTQLKR

>2S.17.pp2-YP_009724390.1

TQLNRA

>2S.17.pp2-Omicron21K-N764K-21L-N764K

TQLKRA

>2S.17.pp3-YP_009724390.1

QLNRAL

>2S.17.pp3-Omicron21K-N764K-21L-N764K

QLKRAL

>2S.17.pp4-YP_009724390.1

LNRALT

>2S.17.pp4-Omicron21K-N764K-21L-N764K

LKRALT

>2S.17.pp5-YP_009724390.1

NRALTG

>2S.17.pp5-Omicron21K-N764K-21L-N764K

KRALTG

>S.18.pp1-YP_009724390.1

TPPIKD

>S.18.pp1-Omicron21K-D796Y-21L-D796Y

TPPIKY

>S.18.pp2-YP_009724390.1

PPIKDF

>S.18.pp2-Omicron21K-D796Y-21L-D796Y

PPIKYF

>S.18.pp3-YP_009724390.1

PIKDFG

>S.18.pp3-Omicron21K-D796Y-21L-D796Y

PIKYFG

>S.18.pp4-YP_009724390.1

IKDFGG

>S.18.pp4-Omicron21K-D796Y-21L-D796Y

IKYFGG

>S.18.pp5-YP_009724390.1

KDFGGF

>S.18.pp5-Omicron21K-D796Y-21L-D796Y

KYFGGF

>2S.18.pp1-YP_009724390.1

PPIKDF

>2S.18.pp1-Omicron21K-D796Y-21L-D796Y

PPIKYF

>2S.18.pp2-YP_009724390.1

PIKDFG

>2S.18.pp2-Omicron21K-D796Y-21L-D796Y

PIKYFG

>2S.18.pp3-YP_009724390.1

IKDFGG

>2S.18.pp3-Omicron21K-D796Y-21L-D796Y

IKYFGG

>2S.18.pp4-YP_009724390.1

KDFGGF

>2S.18.pp4-Omicron21K-D796Y-21L-D796Y

KYFGGF

>2S.18.pp5-YP_009724390.1

DFGGFN

>2S.18.pp5-Omicron21K-D796Y-21L-D796Y

YFGGFN

>S.19.pp1-YP_009724390.1

CAQKFN

>S.19.pp1-Omicron21K-N856K

CAQKFK

>S.19.pp2-YP_009724390.1

AQKFNG

>S.19.pp2-Omicron21K-N856K

AQKFKG

>S.19.pp3-YP_009724390.1

QKFNGL

>S.19.pp3-Omicron21K-N856K

QKFKGL

>S.19.pp4-YP_009724390.1

KFNGLT

>S.19.pp4-Omicron21K-N856K

KFKGLT

>S.19.pp5-YP_009724390.1

FNGLTV

>S.19.pp5-Omicron21K-N856K

FKGLTV

>2S.19.pp1-YP_009724390.1

AQKFNG

>2S.19.pp1-Omicron21K-N856K

AQKFKG

>2S.19.pp2-YP_009724390.1

QKFNGL

>2S.19.pp2-Omicron21K-N856K

QKFKGL

>2S.19.pp3-YP_009724390.1

KFNGLT

>2S.19.pp3-Omicron21K-N856K

KFKGLT

>2S.19.pp4-YP_009724390.1

FNGLTV

>2S.19.pp4-Omicron21K-N856K

FKGLTV

>2S.19.pp5-YP_009724390.1

NGLTVL

>2S.19.pp5-Omicron21K-N856K

KGLTVL

>S.20.pp1-YP_009724390.1

QDVVNQ

>S.20.pp1-Omicron21K-Q954H-21L-Q954H

QDVVNH

>S.20.pp2-YP_009724390.1

DVVNQN

>S.20.pp2-Omicron21K-Q954H-21L-Q954H

DVVNHN

>S.20.pp3-YP_009724390.1

VVNQNA

>S.20.pp3-Omicron21K-Q954H-21L-Q954H

VVNHNA

>S.20.pp4-YP_009724390.1

VNQNAQ

>S.20.pp4-Omicron21K-Q954H-21L-Q954H

VNHNAQ

>S.20.pp5-YP_009724390.1

NQNAQA

>S.20.pp5-Omicron21K-Q954H-21L-Q954H

NHNAQA

>2S.20.pp1-YP_009724390.1

DVVNQN

>2S.20.pp1-Omicron21K-Q954H-21L-Q954H

DVVNHN

>2S.20.pp2-YP_009724390.1

VVNQNA

>2S.20.pp2-Omicron21K-Q954H-21L-Q954H

VVNHNA

>2S.20.pp3-YP_009724390.1

VNQNAQ

>2S.20.pp3-Omicron21K-Q954H-21L-Q954H

VNHNAQ

>S.20.pp4-YP_009724390.1

NQNAQA

>2S.20.pp4-Omicron21K-Q954H-21L-Q954H

NHNAQA

>2S.20.pp5-YP_009724390.1

QNAQAL

>S.20.pp5-Omicron21K-Q954H-21L-Q954H

HNAQAL

>S.21.pp1-YP_009724390.1

KQLSSN

>S.21.pp1-Omicron21K-N969K-21L-N969K

KQLSSK

>S.21.pp2-YP_009724390.1

QLSSNF

>S.21.pp2-Omicron21K-N969K-21L-N969K

QLSSKF

>S.21.pp3-YP_009724390.1

LSSNFG

>S.21.pp3-Omicron21K-N969K-21L-N969K

LSSKFG

>S.21.pp4-YP_009724390.1

SSNFGA

>S.21.pp4-Omicron21K-N969K-21L-N969K

SSKFGA

>S.21.pp5-YP_009724390.1

SNFGAI

>S.21.pp5-Omicron21K-N969K-21L-N969K

SKFGAI

>2S.21.pp1-YP_009724390.1

QLSSNF

>2S.21.pp1-Omicron21K-N969K-21L-N969K

QLSSKF

>2S.21.pp2-YP_009724390.1

LSSNFG

>2S.21.pp2-Omicron21K-N969K-21L-N969K

LSSKFG

>2S.21.pp3-YP_009724390.1

SSNFGA

>2S.21.pp3-Omicron21K-N969K-21L-N969K

SSKFGA

>2S.21.pp4-YP_009724390.1

SNFGAI

>2S.21.pp4-Omicron21K-N969K-21L-N969K

SKFGAI

>2S.21.pp5-YP_009724390.1

NFGAIS

>2S.21.pp5-Omicron21K-N969K-21L-N969K

KFGAIS

>S.22.pp1-YP_009724390.1

VLNDIL

>S.22.pp1-Omicron21K-L981F

VLNDIF

>S.22.pp2-YP_009724390.1

LNDILS

>S.22.pp2-Omicron21K-L981F

LNDIFS

>S.22.pp3-YP_009724390.1

NDILSR

>S.22.pp3-Omicron21K-L981F

NDIFSR

>S.22.pp4-YP_009724390.1

DILSRL

>S.22.pp4-Omicron21K-L981F

DIFSRL

>S.22.pp5-YP_009724390.1

ILSRLD

>S.22.pp5-Omicron21K-L981F

IFSRLD

>2S.22.pp1-YP_009724390.1

LNDILS

>2S.22.pp1-Omicron21K-L981F

LNDIFS

>2S.22.pp2-YP_009724390.1

NDILSR

>2S.22.pp2-Omicron21K-L981F

NDIFSR

>2S.22.pp3-YP_009724390.1

DILSRL

>2S.22.pp3-Omicron21K-L981F

DIFSRL

>2S.22.pp4-YP_009724390.1

ILSRLD

>2S.22.pp4-Omicron21K-L981F

IFSRLD

>2S.22.pp5-YP_009724390.1

LSRLDK

>2S.22.pp5-Omicron21K-L981F

FSRLDK

>N.1.pp1-YP_009724397.2

NQRNAP

>N.1.pp1-Omicron21K-P13L-21L-P13L

NQRNAL

>N.1.pp2-YP_009724397.2

QRNAPR

>N.1.pp2-Omicron21K-P13L-21L-P13L

QRNALR

>N.1.pp3-YP_009724397.2

RNAPRI

>N.1.pp3-Omicron21K-P13L-21L-P13L

RNALRI

>N.1.pp4-YP_009724397.2

NAPRIT

>N.1.pp4-Omicron21K-P13L-21L-P13L

NALRIT

>N.1.pp5-YP_009724397.2

APRITF

>N.1.pp5-Omicron21K-P13L-21L-P13L

ALRITF

>2N.1.pp1-YP_009724397.2

QRNAPR

>2N.1.pp1-Omicron21K-P13L-21L-P13L

QRNALR

>2N.1.pp2-YP_009724397.2

RNAPRI

>2N.1.pp2-Omicron21K-P13L-21L-P13L

RNALRI

>2N.1.pp3-YP_009724397.2

NAPRIT

>2N.1.pp3-Omicron21K-P13L-21L-P13L

NALRIT

>2N.1.pp4-YP_009724397.2

APRITF

>2N.1.pp4-Omicron21K-P13L-21L-P13L

ALRITF

>2N.1.pp5-YP_009724397.2

PRITFG

>2N.1.pp5-Omicron21K-P13L-21L-P13L

LRITFG

>N.2.pp1.1-YP_009724397.2

SNQNGE

>N.2.pp1.2-YP_009724397.2

NQNGER

>N.2.pp1.3-YP_009724397.2

QNGERS

>N.2.pp1.4-YP_009724397.2

NGERSG

>N.2.pp1-Omicron21K-delE31-delR32-delS33-21L-delE31-delR32-delS33

SNQNGG

>N.2.pp2.1-YP_009724397.2

NQNGER

>N.2.pp2.2-YP_009724397.2

QNGERS

>N.2.pp2.3-YP_009724397.2

NGERSG

>N.2.pp2.4-YP_009724397.2

GERSGA

>N.2.pp2-Omicron21K-delE31-delR32-delS33-21L-delE31-delR32-delS33

NQNGGA

>N.2.pp3.1-YP_009724397.2

QNGERS

>N.2.pp3.2-YP_009724397.2

NGERSG

>N.2.pp3.3-YP_009724397.2

GERSGA

>N.2.pp3.4-YP_009724397.2

ERSGAR

>N.2.pp3-Omicron21K-delE31-delR32-delS33-21L-delE31-delR32-delS33

QNGGAR

>N.2.pp4.1-YP_009724397.2

NGERSG

>N.2.pp4.2-YP_009724397.2

GERSGA

>N.2.pp4.3-YP_009724397.2

ERSGAR

>N.2.pp4.4-YP_009724397.2

RSGARS

>N.2.pp4-Omicron21K-delE31-delR32-delS33-21L-delE31-delR32-delS33

NGGARS

>2N.2.pp1.1-YP_009724397.2

NQNGER

>2N.2.pp1.2-YP_009724397.2

QNGERS

>2N.2.pp1.3-YP_009724397.2

NGERSG

>2N.2.pp1.4-YP_009724397.2

GERSGA

>2N.2.pp1-Omicron21K-delE31-delR32-delS33-21L-delE31-delR32-delS33

NQNGGA

>2N.2.pp2.1-YP_009724397.2

QNGERS

>2N.2.pp2.2-YP_009724397.2

NGERSG

>2N.2.pp2.3-YP_009724397.2

GERSGA

>2N.2.pp2.4-YP_009724397.2

ERSGAR

>2N.2.pp2-Omicron21K-delE31-delR32-delS33-21L-delE31-delR32-delS33

QNGGAR

>2N.2.pp3.1-YP_009724397.2

NGERSG

>2N.2.pp3.2-YP_009724397.2

GERSGA

>2N.2.pp3.3-YP_009724397.2

ERSGAR

>2N.2.pp3.4-YP_009724397.2

RSGARS

>2N.2.pp3-Omicron21K-delE31-delR32-delS33-21L-delE31-delR32-delS33

NGGARS

>2N.2.pp4.1-YP_009724397.2

GERSGA

>2N.2.pp4.2-YP_009724397.2

ERSGAR

>2N.2.pp4.3-YP_009724397.2

RSGARS

>2N.2.pp4.4-YP_009724397.2

SGARSK

>2N.2.pp4-Omicron21K-delE31-delR32-delS33-21L-delE31-delR32-delS33

GGARSK

>N.3.pp1-YP_009724397.2

TPGSSR

>N.3.pp1-Omicron21K-R203K-G204R-21L-R203K-G204R

TPGSSK

>N.3.pp2-YP_009724397.2

PGSSRG

>N.3.pp2-Omicron21K-R203K-G204R-21L-R203K-G204R

PGSSKR

>N.3.pp3-YP_009724397.2

GSSRGT

>N.3.pp3-Omicron21K-R203K-G204R-21L-R203K-G204R

GSSKRT

>N.3.pp4-YP_009724397.2

SSRGTS

>N.3.pp4-Omicron21K-R203K-G204R-21L-R203K-G204R

SSKRTS

>N.3.pp5-YP_009724397.2

SRGTSP

>N.3.pp5-Omicron21K-R203K-G204R-21L-R203K-G204R

SKRTSP

>N.3.pp6-YP_009724397.2

RGTSPA

>N.3.pp6-Omicron21K-R203K-G204R-21L-R203K-G204R

KRTSPA

>2N.3.pp1-YP_009724397.2

PGSSRG

>2N.3.pp1-Omicron21K-R203K-G204R-21L-R203K-G204R

PGSSKR

>2N.3.pp2-YP_009724397.2

GSSRGT

>2N.3.pp2-Omicron21K-R203K-G204R-21L-R203K-G204R

GSSKRT

>2N.3.pp3-YP_009724397.2

SSRGTS

>2N.3.pp3-Omicron21K-R203K-G204R-21L-R203K-G204R

SSKRTS

>2N.3.pp4-YP_009724397.2

SRGTSP

>2N.3.pp4-Omicron21K-R203K-G204R-21L-R203K-G204R

SKRTSP

>2N.3.pp5-YP_009724397.2

RGTSPA

>2N.3.pp5-Omicron21K-R203K-G204R-21L-R203K-G204R

KRTSPA

>2N.3.pp6-YP_009724397.2

GTSPAR

>2N.3.pp6-Omicron21K-R203K-G204R-21L-R203K-G204R

RTSPAR

>Orf1ab.3.pp1-YP_009724389.1

KVLNEK

>Orf1ab.3.pp1-Omicron21K-K856R

KVLNER

>Orf1ab.3.pp2-YP_009724389.1

VLNEKC

>Orf1ab.3.pp2-Omicron21K-K856R

VLNERC

>Orf1ab.3.pp3-YP_009724389.1

LNEKCS

>Orf1ab.3.pp3-Omicron21K-K856R

LNERCS

>Orf1ab.3.pp4-YP_009724389.1

NEKCSA

>Orf1ab.3.pp4-Omicron21K-K856R

NERCSA

>Orf1ab.3.pp5-YP_009724389.1

EKCSAY

>Orf1ab.3.pp5-Omicron21K-K856R

ERCSAY

>2Orf1ab.3.pp1-YP_009724389.1

VLNEKC

>2Orf1ab.3.pp1-Omicron21K-K856R

VLNERC

>2Orf1ab.3.pp2-YP_009724389.1

LNEKCS

>2Orf1ab.3.pp2-Omicron21K-K856R

LNERCS

>2Orf1ab.3.pp3-YP_009724389.1

NEKCSA

>2Orf1ab.3.pp3-Omicron21K-K856R

NERCSA

>2Orf1ab.3.pp4-YP_009724389.1

EKCSAY

>2Orf1ab.3.pp4-Omicron21K-K856R

ERCSAY

>2Orf1ab.3.pp5-YP_009724389.1

KCSAYT

>2Orf1ab.3.pp5-Omicron21K-K856R

RCSAYT

>Orf1ab.5.pp1.1-YP_009724389.1

KPANNS

>Orf1ab.5.pp1.2-YP_009724389.1

PANNSL

>Orf1ab.5.pp1-Omicron21K-delS2083-L2084I

KPANNI

>Orf1ab.5.pp2.1-YP_009724389.1

PANNSL

>Orf1ab.5.pp2.2-YP_009724389.1

ANNSLK

>Orf1ab.5.pp2-Omicron21K-delS2083-L2084I

PANNIK

>Orf1ab.5.pp3.1-YP_009724389.1

ANNSLK

>Orf1ab.5.pp3.2-YP_009724389.1

NNSLKI

>Orf1ab.5.pp3-Omicron21K-delS2083-L2084I

ANNIKI

>Orf1ab.5.pp4.1-YP_009724389.1

NNSLKI

>Orf1ab.5.pp4.2-YP_009724389.1

NSLKIT

>Orf1ab.5.pp4-Omicron21K-delS2083-L2084I

NNIKIT

>Orf1ab.5.pp5.1-YP_009724389.1

NSLKIT

>Orf1ab.5.pp5.2-YP_009724389.1

SLKITE

>Orf1ab.5.pp5-Omicron21K-delS2083-L2084I

NIKITE

>2Orf1ab.5.pp1.1-YP_009724389.1

PANNSL

>2Orf1ab.5.pp1.2-YP_009724389.1

ANNSLK

>2Orf1ab.5.pp1-Omicron21K-delS2083-L2084I

PANNIK

>2Orf1ab.5.pp2.1-YP_009724389.1

ANNSLK

>2Orf1ab.5.pp2.2-YP_009724389.1

NNSLKI

>2Orf1ab.5.pp2-Omicron21K-delS2083-L2084I

ANNIKI

>2Orf1ab.5.pp3.1-YP_009724389.1

NNSLKI

>2Orf1ab.5.pp3.2-YP_009724389.1

NSLKIT

>2Orf1ab.5.pp3-Omicron21K-delS2083-L2084I

NNIKIT

>2Orf1ab.5.pp4.1-YP_009724389.1

NSLKIT

>2Orf1ab.5.pp4.2-YP_009724389.1

SLKITE

>2Orf1ab.5.pp4-Omicron21K-delS2083-L2084I

NIKITE

>2Orf1ab.5.pp5.1-YP_009724389.1

SLKITE

>2Orf1ab.5.pp5.2-YP_009724389.1

LKITEE

>2Orf1ab.5.pp5-Omicron21K-delS2083-L2084I

IKITEE

>Orf1ab.6.pp1-YP_009724389.1

KSHNIA

>Orf1ab.6.pp1-Omicron21K-A2710T

KSHNIT

>Orf1ab.6.pp2-YP_009724389.1

SHNIAL

>Orf1ab.6.pp2-Omicron21K-A2710T

SHNITL

>Orf1ab.6.pp3-YP_009724389.1

HNIALI

>Orf1ab.6.pp3-Omicron21K-A2710T

HNITLI

>Orf1ab.6.pp4-YP_009724389.1

NIALIW

>Orf1ab.6.pp4-Omicron21K-A2710T

NITLIW

>Orf1ab.6.pp5-YP_009724389.1

IALIWN

>Orf1ab.6.pp5-Omicron21K-A2710T

ITLIWN

>2Orf1ab.6.pp1-YP_009724389.1

SHNIAL

>2Orf1ab.6.pp1-Omicron21K-A2710T

SHNITL

>2Orf1ab.6.pp2-YP_009724389.1

HNIALI

>2Orf1ab.6.pp2-Omicron21K-A2710T

HNITLI

>2Orf1ab.6.pp3-YP_009724389.1

NIALIW

>2Orf1ab.6.pp3-Omicron21K-A2710T

NITLIW

>2Orf1ab.6.pp4-YP_009724389.1

IALIWN

>2Orf1ab.6.pp4-Omicron21K-A2710T

ITLIWN

>2Orf1ab.6.pp5-YP_009724389.1

ALIWNV

>2Orf1ab.6.pp5-Omicron21K-A2710T

TLIWNV

>Orf1ab.10.pp1-YP_009724389.1

YQPPQT

>Orf1ab.10.pp1-Omicron21K-T3255I-21L-T3255I

YQPPQI

>Orf1ab.10.pp2-YP_009724389.1

QPPQTS

>Orf1ab.10.pp2-Omicron21K-T3255I-21L-T3255I

QPPQIS

>Orf1ab.10.pp3-YP_009724389.1

PPQTSI

>Orf1ab.10.pp3-Omicron21K-T3255I-21L-T3255I

PPQISI

>Orf1ab.10.pp4-YP_009724389.1

PQTSIT

>Orf1ab.10.pp4-Omicron21K-T3255I-21L-T3255I

PQISIT

>Orf1ab.10.pp5-YP_009724389.1

QTSITS

>Orf1ab.10.pp5-Omicron21K-T3255I-21L-T3255I

QISITS

>2Orf1ab.10.pp1-YP_009724389.1

QPPQTS

>2Orf1ab.10.pp1-Omicron21K-T3255I-21L-T3255I

QPPQIS

>2Orf1ab.10.pp2-YP_009724389.1

PPQTSI

>2Orf1ab.10.pp2-Omicron21K-T3255I-21L-T3255I

PPQISI

>2Orf1ab.10.pp3-YP_009724389.1

PQTSIT

>2Orf1ab.10.pp3-Omicron21K-T3255I-21L-T3255I

PQISIT

>2Orf1ab.10.pp4-YP_009724389.1

QTSITS

>2Orf1ab.10.pp4-Omicron21K-T3255I-21L-T3255I

QISITS

>2Orf1ab.10.pp5-YP_009724389.1

TSITSA

>2Orf1ab.10.pp5-Omicron21K-T3255I-21L-T3255I

ISITSA

>Orf1ab.11.pp1-YP_009724389.1

QCAMRP

>Orf1ab.11.pp1-Omicron21K-P3395H-21L-P3395H

QCAMRH

>Orf1ab.11.pp2-YP_009724389.1

CAMRPN

>Orf1ab.11.pp2-Omicron21K-P3395H-21L-P3395H

CAMRHN

>Orf1ab.11.pp3-YP_009724389.1

AMRPNF

>Orf1ab.11.pp3-Omicron21K-P3395H-21L-P3395H

AMRHNF

>Orf1ab.11.pp4-YP_009724389.1

MRPNFT

>Orf1ab.11.pp4-Omicron21K-P3395H-21L-P3395H

MRHNFT

>Orf1ab.11.pp5-YP_009724389.1

RPNFTI

>Orf1ab.11.pp5-Omicron21K-P3395H-21L-P3395H

RHNFTI

>2Orf1ab.11.pp1-YP_009724389.1

CAMRPN

>2Orf1ab.11.pp1-Omicron21K-P3395H-21L-P3395H

CAMRHN

>2Orf1ab.11.pp2-YP_009724389.1

AMRPNF

>2Orf1ab.11.pp2-Omicron21K-P3395H-21L-P3395H

AMRHNF

>2Orf1ab.11.pp3-YP_009724389.1

MRPNFT

>2Orf1ab.11.pp3-Omicron21K-P3395H-21L-P3395H

MRHNFT

>2Orf1ab.11.pp4-YP_009724389.1

RPNFTI

>2Orf1ab.11.pp4-Omicron21K-P3395H-21L-P3395H

RHNFTI

>2Orf1ab.11.pp5-YP_009724389.1

PNFTIK

\>2Orf1ab.11.pp5-Omicron21K-P3395H-21L-P3395H

HNFTIK

\>Orf1ab.12.pp1.1-YP_009724389.1

MVDTSL

\>Orf1ab.12.pp1.2-YP_009724389.1

VDTSLS

\>Orf1ab.12.pp1.3-YP_009724389.1

DTSLSG

\>Orf1ab.12.pp1.4-YP_009724389.1

TSLSGF

\>Orf1ab.12.1.pp1-Omicron21K-delL3674-delS3675-delG3676

MVDTSF

\>Orf1ab.12.pp2.1-YP_009724389.1

VDTSLS

\>Orf1ab.12.pp2.2-YP_009724389.1

DTSLSG

\>Orf1ab.12.pp2.3-YP_009724389.1

TSLSGF

\>Orf1ab.12.pp2.4-YP_009724389.1

SLSGFK

\>Orf1ab.12.1.pp2-Omicron21K-delL3674-delS3675-delG3676

VDTSFK

\>Orf1ab.12.pp3.1-YP_009724389.1

DTSLSG

\>Orf1ab.12.pp3.2-YP_009724389.1

TSLSGF

>Orf1ab.12.pp3.3-YP_009724389.1

SLSGFK

>Orf1ab.12.pp3.4-YP_009724389.1

LSGFKL

>Orf1ab.12.1.pp3-Omicron21K-delL3674-delS3675-delG3676

DTSFKL

>Orf1ab.12.pp4.1-YP_009724389.1

TSLSGF

>Orf1ab.12.pp4.2-YP_009724389.1

SLSGFK

>Orf1ab.12.pp4.3-YP_009724389.1

LSGFKL

>Orf1ab.12.pp4.4-YP_009724389.1

SGFKLK

>Orf1ab.12.1.pp4-Omicron21K-delL3674-delS3675-delG3676

TSFKLK

>2Orf1ab.12.pp1.1-YP_009724389.1

VDTSLS

>2Orf1ab.12.pp1.2-YP_009724389.1

DTSLSG

>2Orf1ab.12.pp1.3-YP_009724389.1

TSLSGF

>2Orf1ab.12.pp1.4-YP_009724389.1

SLSGFK

>2Orf1ab.12.1.pp1-Omicron21K-delL3674-delS3675-delG3676

VDTSFK

>2Orf1ab.12.pp2.1-YP_009724389.1

DTSLSG

>2Orf1ab.12.pp2.2-YP_009724389.1

TSLSGF

>2Orf1ab.12.pp2.3-YP_009724389.1

SLSGFK

>2Orf1ab.12.pp2.4-YP_009724389.1

LSGFKL

>2Orf1ab.12.1.pp2-Omicron21K-delL3674-delS3675-delG3676

DTSFKL

>2Orf1ab.12.pp3.1-YP_009724389.1

TSLSGF

>2Orf1ab.12.pp3.2-YP_009724389.1

SLSGFK

>2Orf1ab.12.pp3.3-YP_009724389.1

LSGFKL

>2Orf1ab.12.pp3.4-YP_009724389.1

SGFKLK

>2Orf1ab.12.1.pp3-Omicron21K-delL3674-delS3675-delG3676

TSFKLK

>2Orf1ab.12.pp4.1-YP_009724389.1

SLSGFK

>2Orf1ab.12.pp4.2-YP_009724389.1

LSGFKL

>2Orf1ab.12.pp4.3-YP_009724389.1

SGFKLK

>2Orf1ab.12.pp4.4-YP_009724389.1

GFKLKD

>2Orf1ab.12.1.pp4-Omicron21K-delL3674-delS3675-delG3676

SFKLKD

>Orf1ab.13.pp1-YP_009724389.1

FLARGI

>Orf1ab.13.pp1-Omicron21K-I3758V

FLARGV

>Orf1ab.13.pp2-YP_009724389.1

LARGIV

>Orf1ab.13.pp2-Omicron21K-I3758V

LARGVV

>Orf1ab.13.pp3-YP_009724389.1

ARGIVF

>Orf1ab.13.pp3-Omicron21K-I3758V

ARGVVF

>Orf1ab.13.pp4-YP_009724389.1

RGIVFM

>Orf1ab.13.pp4-Omicron21K-I3758V

RGVVFM

>Orf1ab.13.pp5-YP_009724389.1

GIVFMC

>Orf1ab.13.pp5-Omicron21K-I3758V

GVVFMC

>2Orf1ab.13.pp1-YP_009724389.1

LARGIV

>2Orf1ab.13.pp1-Omicron21K-I3758V

LARGVV

>2Orf1ab.13.pp2-YP_009724389.1

ARGIVF

>2Orf1ab.13.pp2-Omicron21K-I3758V

ARGVVF

>2Orf1ab.13.pp3-YP_009724389.1

RGIVFM

>2Orf1ab.13.pp3-Omicron21K-I3758V

RGVVFM

>2Orf1ab.13.pp4-YP_009724389.1

GIVFMC

>2Orf1ab.13.pp4-Omicron21K-I3758V

GVVFMC

>2Orf1ab.13.pp5-YP_009724389.1

IVFMCV

>2Orf1ab.13.pp5-Omicron21K-I3758V

VVFMCV

>Orf1ab.1.pp1-YP_009724389.1

STVFPP

>Orf1ab.1.pp1-Omicron21K-P314L-21L-P314L

STVFPL

>Orf1ab.1.pp2-YP_009724389.1

TVFPPT

>Orf1ab.1.pp2-Omicron21K-P314L-21L-P314L

TVFPLT

>Orf1ab.1.pp3-YP_009724389.1

VFPPTS

>Orf1ab.1.pp3-Omicron21K-P314L-21L-P314L

VFPLTS

>Orf1ab.1.pp4-YP_009724389.1

FPPTSF

>Orf1ab.1.pp4-Omicron21K-P314L-21L-P314L

FPLTSF

>Orf1ab.1.pp5-YP_009724389.1

PPTSFG

>Orf1ab.1.pp5-Omicron21K-P314L-21L-P314L

PLTSFG

>2Orf1ab.1.pp1-YP_009724389.1

TVFPPT

>2Orf1ab.1.pp1-Omicron21K-P314L-21L-P314L

TVFPLT

>2Orf1ab.1.pp2-YP_009724389.1

VFPPTS

>2Orf1ab.1.pp2-Omicron21K-P314L-21L-P314L

VFPLTS

>2Orf1ab.1.pp3-YP_009724389.1

FPPTSF

>2Orf1ab.1.pp3-Omicron21K-P314L-21L-P314L

FPLTSF

>2Orf1ab.1.pp4-YP_009724389.1

PPTSFG

>2Orf1ab.1.pp4-Omicron21K-P314L-21L-P314L

PLTSFG

>2Orf1ab.1.pp5-YP_009724389.1

PTSFGP

>2Orf1ab.1.pp5-Omicron21K-P314L-21L-P314L

LTSFGP

>Orf1ab.3.pp1-YP_009724389.1

GLCVDI

>Orf1ab.3.pp1-Omicron21K-I1566V-21L-I1566V

GLCVDV

>Orf1ab.3.pp2-YP_009724389.1

LCVDIP

>Orf1ab.3.pp2-Omicron21K-I1566V-21L-I1566V

LCVDVP

>Orf1ab.3.pp3-YP_009724389.1

CVDIPG

>Orf1ab.3.pp3-Omicron21K-I1566V-21L-I1566V

CVDVPG

>Orf1ab.3.pp4-YP_009724389.1

VDIPGI

>Orf1ab.3.pp4-Omicron21K-I1566V-21L-I1566V

VDVPGI

>Orf1ab.3.pp5-YP_009724389.1

DIPGIP

>Orf1ab.3.pp5-Omicron21K-I1566V-21L-I1566V

DVPGIP

>2Orf1ab.3.pp1-YP_009724389.1

LCVDIP

>2Orf1ab.3.pp1-Omicron21K-I1566V-21L-I1566V

LCVDVP

>2Orf1ab.3.pp2-YP_009724389.1

CVDIPG

>2Orf1ab.3.pp2-Omicron21K-I1566V-21L-I1566V

CVDVPG

>2Orf1ab.3.pp3-YP_009724389.1

VDIPGI

>2Orf1ab.3.pp3-Omicron21K-I1566V-21L-I1566V

VDVPGI

>2Orf1ab.3.pp4-YP_009724389.1

DIPGIP

>2Orf1ab.3.pp4-Omicron21K-I1566V-21L-I1566V

DVPGIP

>2Orf1ab.3.pp5-YP_009724389.1

IPGIPK

>2Orf1ab.3.pp5-Omicron21K-I1566V-21L-I1566V

VPGIPK

>Orf9b.1.pp1-P0DTD2

ISEMHP

>Orf9b.1.pp1-Omicron21K-P10S-21L-LP10S

ISEMHS

>Orf9b.1.pp2-P0DTD2

SEMHPA

>Orf9b.1.pp2-Omicron21K-P10S-21L-LP10S

SEMHSA

>Orf9b.1.pp3-P0DTD2

EMHPAL

>Orf9b.1.pp3-Omicron21K-P10S-21L-LP10S

EMHSAL

>Orf9b.1.pp4-P0DTD2

MHPALR

>Orf9b.1.pp4-Omicron21K-P10S-21L-LP10S

MHSALR

>Orf9b.1.pp5-P0DTD2

HPALRL

>Orf9b.1.pp5-Omicron21K-P10S-21L-LP10S

HSALRL

>2Orf9b.1.pp1-P0DTD2

SEMHPA

>2Orf9b.1.pp1-Omicron21K-P10S-21L-LP10S

SEMHSA

>2Orf9b.1.pp2-P0DTD2

EMHPAL

>2Orf9b.1.pp2-Omicron21K-P10S-21L-LP10S

EMHSAL

>Orf9b.1.pp3-P0DTD2

MHPALR

>2Orf9b.1.pp3-Omicron21K-P10S-21L-LP10S

MHSALR

>2Orf9b.1.pp4-P0DTD2

HPALRL

>2Orf9b.1.pp4-Omicron21K-P10S-21L-LP10S

HSALRL

>2Orf9b.1.pp5-P0DTD2

PALRLV

>2Orf9b.1.pp5-Omicron21K-P10S-21L-LP10S

SALRLV

>Orf9b.2.pp1.1-P0DTD2

AVTRME

>Orf9b.2.pp1.2-P0DTD2

VTRMEN

>Orf9b.2.pp1.3-P0DTD2

TRMENA

>Orf9b.2.pp1.4-P0DTD2

RMENAV

>Orf9b.2.pp1-Omicron21K-delE27-delN28-delA29-21L-delE27-delN28-delA29

AVTRMV

>Orf9b.2.pp2.1-P0DTD2

VTRMEN

>Orf9b.2.pp2.2-P0DTD2

TRMENA

>Orf9b.2.pp2.3-P0DTD2

RMENAV

>Orf9b.2.pp2.4-P0DTD2

MENAVG

>Orf9b.2.pp2-Omicron21K-delE27-delN28-delA29-21L-delE27-delN28-delA29

VTRMVG

>Orf9b.2.pp3.1-P0DTD2

TRMENA

>Orf9b.2.pp3.2-P0DTD2

RMENAV

>Orf9b.2.pp3.3-P0DTD2

MENAVG

>Orf9b.2.pp3.4-P0DTD2

ENAVGR

>Orf9b.2.pp3-Omicron21K-delE27-delN28-delA29-21L-delE27-delN28-delA29

TRMVGR

>Orf9b.2.pp4.1-P0DTD2

RMENAV

>Orf9b.2.pp4.2-P0DTD2

MENAVG

>Orf9b.2.pp4.3-P0DTD2

ENAVGR

>Orf9b.2.pp4.4-P0DTD2

NAVGRD

>Orf9b.2.pp4-Omicron21K-delE27-delN28-delA29-21L-delE27-delN28-delA29

RMVGRD

>2Orf9b.2.pp1.1-P0DTD2

VTRMEN

>2Orf9b.2.pp1.2-P0DTD2

TRMENA

>2Orf9b.2.pp1.3-P0DTD2

RMENAV

>2Orf9b.2.pp1.4-P0DTD2

MENAVG

>2Orf9b.2.pp1-Omicron21K-delE27-delN28-delA29-21L-delE27-delN28-delA29

VTRMVG

>2Orf9b.2.pp2.1-P0DTD2

TRMENA

>2Orf9b.2.pp2.2-P0DTD2

RMENAV

>2Orf9b.2.pp2.3-P0DTD2

MENAVG

>2Orf9b.2.pp2.4-P0DTD2

ENAVGR

>2Orf9b.2.pp2-Omicron21K-delE27-delN28-delA29-21L-delE27-delN28-delA29

TRMVGR

>2Orf9b.2.pp3.1-P0DTD2

RMENAV

>2Orf9b.2.pp3.2-P0DTD2

MENAVG

>2Orf9b.2.pp3.3-P0DTD2

ENAVGR

>2Orf9b.2.pp3.4-P0DTD2

NAVGRD

>2Orf9b.2.pp3-Omicron21K-delE27-delN28-delA29-21L-delE27-delN28-delA29

RMVGRD

>2Orf9b.2.pp4.1-P0DTD2

MENAVG

>2Orf9b.2.pp4.2-P0DTD2

ENAVGR

>2Orf9b.2.pp4.3-P0DTD2

NAVGRD

>2Orf9b.2.pp4.4-P0DTD2

AVGRDQ

>2Orf9b.2.pp4-Omicron21K-delE27-delN28-delA29-21L-delE27-delN28-delA29

MVGRDQ

>E.1.pp1-YP_009724392.1

FVSEET

>E.1.pp1-Omicron21K-T9I-21L-T9I

FVSEEI

>E.1.pp2-YP_009724392.1

VSEETG

>E.1.pp2-Omicron21K-T9I-21L-T9I

VSEEIG

>E.1.pp3-YP_009724392.1

SEETGT

>E.1.pp3-Omicron21K-T9I-21L-T9I

SEEIGT

>E.1.pp4-YP_009724392.1

EETGTL

>E.1.pp4-Omicron21K-T9I-21L-T9I

EEIGTL

>E.1.pp5-YP_009724392.1

ETGTLI

>E.1.pp5-Omicron21K-T9I-21L-T9I

EIGTLI

>2E.1.pp1-YP_009724392.1

VSEETG

>2E.1.pp1-Omicron21K-T9I-21L-T9I

VSEEIG

>2E.1.pp2-YP_009724392.1

SEETGT

>2E.1.pp2-Omicron21K-T9I-21L-T9I

SEEIGT

>2E.1.pp3-YP_009724392.1

EETGTL

>2E.1.pp3-Omicron21K-T9I-21L-T9I

EEIGTL

>2E.1.pp4-YP_009724392.1

ETGTLI

>2E.1.pp4-Omicron21K-T9I-21L-T9I

EIGTLI

>2E.1.pp5-YP_009724392.1

TGTLIV

>2E.1.pp5-Omicron21K-T9I-21L-T9I

IGTLIV

>2M.1.pp1-YP_009724393.1

MADSNG

>2M.1.pp1-Omicron21K-D3G

MAGSNG

>2M.1.pp2-YP_009724393.1

ADSNGT

>2M.1.pp2-Omicron21K-D3G

AGSNGT

>2M.1.pp3-YP_009724393.1

DSNGTI

>2M.1.pp3-Omicron21K-D3G

GSNGTI

>M.2.pp1-YP_009724393.1

KKLLEQ

>M.2.pp1-Omicron21K-Q19E-21L-Q19E

KKLLEE

>M.2.pp2-YP_009724393.1

KLLEQW

>M.2.pp2-Omicron21K-Q19E-21L-Q19E

KLLEEW

>M.2.pp3-YP_009724393.1

LLEQWN

>M.2.pp3-Omicron21K-Q19E-21L-Q19E

LLEEWN

>M.2.pp4-YP_009724393.1

LEQWNL

>M.2.pp4-Omicron21K-Q19E-21L-Q19E

LEEWNL

>M.2.pp5-YP_009724393.1

EQWNLV

>M.2.pp5-Omicron21K-Q19E-21L-Q19E

EEWNLV

>2M.2.pp1-YP_009724393.1

KLLEQW

>2M.2.pp1-Omicron21K-Q19E-21L-Q19E

KLLEEW

>2M.2.pp2-YP_009724393.1

LLEQWN

>2M.2.pp2-Omicron21K-Q19E-21L-Q19E

LLEEWN

>2M.2.pp3-YP_009724393.1

LEQWNL

>2M.2.pp3-Omicron21K-Q19E-21L-Q19E

LEEWNL

>2M.2.pp4-YP_009724393.1

EQWNLV

>2M.2.pp4-Omicron21K-Q19E-21L-Q19E

EEWNLV

>2M.2.pp5-YP_009724393.1

QWNLVI

>2M.2.pp5-Omicron21K-Q19E-21L-Q19E

EWNLVI

>M.3.pp1-YP_009724393.1

WPVTLA

>M.3.pp1-Omicron21K-A63T-21L-A63T

WPVTLT

>M.3.pp2-YP_009724393.1

PVTLAC

>M.3.pp2-Omicron21K-A63T-21L-A63T

PVTLTC

>M.3.pp3-YP_009724393.1

VTLACF

>M.3.pp3-Omicron21K-A63T-21L-A63T

VTLTCF

>M.3.pp4-YP_009724393.1

TLACFV

>M.3.pp4-Omicron21K-A63T-21L-A63T

TLTCFV

>M.3.pp5-YP_009724393.1

LACFVL

>M.3.pp5-Omicron21K-A63T-21L-A63T

LTCFVL

>2M.3.pp1-YP_009724393.1

PVTLAC

>2M.3.pp1-Omicron21K-A63T-21L-A63T

PVTLTC

>2M.3.pp2-YP_009724393.1

VTLACF

>2M.3.pp2-Omicron21K-A63T-21L-A63T

VTLTCF

>2M.3.pp3-YP_009724393.1

TLACFV

>2M.3.pp3-Omicron21K-A63T-21L-A63T

TLTCFV

>2M.3.pp4-YP_009724393.1

LACFVL

>2M.3.pp4-Omicron21K-A63T-21L-A63T

LTCFVL

>2M.3.pp5-YP_009724393.1

ACFVLA

>2M.3.pp5-Omicron21K-A63T-21L-A63T

TCFVLA
