# Supplementary material for: Shared 6mer Peptides of Human and Omicron (21K and 21L) at SARS-CoV-2 Mutation Sites
Source: Antibodies (Basel). 2022 Oct 25;11(4):68. doi: 10.3390/antib11040068 (PMC9680445; doi:10.3390/antib11040068)
Supplement: Supplementary file 1 [file antibodies-11-00068-s001.zip › DocumentS2.pdf]

Blastp search input sequences involving mutations specific for Omicron 21L

>S.1.p1.pp1-YP_009724390.1

QCVNLT

>S.1.p1.pp1-Omicron21L-T19I

QCVNLI

>S.1.p1.pp2-YP_009724390.1

CVNLTT

>S.1.p1.pp2-Omicron21L-T19I

CVNLIT

>S.1.p1.pp3-YP_009724390.1

VNLTTR

>S.1.p1.pp3-Omicron21L-T19I

VNLITR

>S.1.p1.pp4-YP_009724390.1

NLTTRT

>S.1.p1.pp4-Omicron21L-T19I

NLITRT

>S.1.p1.pp5-YP_009724390.1

LTTRTQ

>S.1.p1.pp5-Omicron21L-T19I

LITRTQ

>2S.1.p1.pp1-YP_009724390.1

CVNLTT

>2S.1.p1.pp1-Omicron21L-T19I

CVNLIT

>2S.1.p1.pp2-YP_009724390.1

VNLTTR

>2S.1.p1.pp2-Omicron21L-T19I

VNLITR

>2S.1.p1.pp3-YP_009724390.1

NLTTRT

>2S.1.p1.pp3-Omicron21L-T19I

NLITRT

>2S.1.p1.pp4-YP_009724390.1

LTTRTQ

>2S.1.p1.pp4-Omicron21L-T19I

LITRTQ

>2S.1.p1.pp5-YP_009724390.1

TTRTQL

>2S.1.p1.pp5-Omicron21L-T19I

ITRTQS

>S.1.p2.pp1-YP_009724390.1

TTRTQL

>S.1.p2.pp1-Omicron21L-T19I-delL24-delP25-delP26-A27S

ITRTQS

>S.1.p2.pp2.1-YP_009724390.1

TRTQLP

>S.1.p2.pp2.2-YP_009724390.1

RTQLPP

>S.1.p2.pp2.3-YP_009724390.1

TQLPPA

>S.1.p2.pp2.4-YP_009724390.1

QLPPAY

>S.1.p2.pp2-Omicron21L-T19I-delL24-delP25-delP26-A27S

TRTQSY

>S.1.p2.pp3.1-YP_009724390.1

RTQLPP

>S.1.p2.pp3.2-YP_009724390.1

TQLPPA

>S.1.p2.pp3.3-YP_009724390.1

QLPPAY

>S.1.p2.pp3.4-YP_009724390.1

LPPAYT

>S.1.p2.pp3-Omicron21L-T19I-delL24-delP25-delP26-A27S

RTQSYT

>S.1.p2.pp4.1-YP_009724390.1

TQLPPA

>S.1.p2.pp4.2-YP_009724390.1

QLPPAY

>S.1.p2.pp4.3-YP_009724390.1

LPPAYT

>S.1.p2.pp4.4-YP_009724390.1

PPAYTN

>S.1.p2.pp4-Omicron21L-T19I-delL24-delP25-delP26-A27S

TQSYTN

>S.1.p2.pp5.1-YP_009724390.1

QLPPAY

>S.1.p2.pp5.2-YP_009724390.1

LPPAYT

>S.1.p2.pp5.3-YP_009724390.1

PPAYTN

>S.1.p2.pp5.4-YP_009724390.1

PAYTNS

>S.1.p2.pp5-Omicron21L-T19I-delL24-delP25-delP26-A27S

QSYTNS

>2S.1.p2.pp1.1-YP_009724390.1

TRTQLP

>2S.1.p2.pp1.2-YP_009724390.1

RTQLPP

>2S.1.p2.pp1.3-YP_009724390.1

TQLPPA

>2S.1.p2.pp1.4-YP_009724390.1

QLPPAY

>2S.1.p2.pp1-Omicron21L-T19I-delL24-delP25-delP26-A27S

TRTQSY

>2S.1.p2.pp2.1-YP_009724390.1

RTQLPP

>2S.1.p2.pp2.2-YP_009724390.1

TQLPPA

>2S.1.p2.pp2.3-YP_009724390.1

QLPPAY

>2S.1.p2.pp2.4-YP_009724390.1

LPPAYT

>2S.1.p2.pp2-Omicron21L-T19I-delL24-delP25-delP26-A27S

RTQSYT

>2S.1.p2.pp3.1-YP_009724390.1

TQLPPA

>2S.1.p2.pp3.2-YP_009724390.1

QLPPAY

>2S.1.p2.pp3.3-YP_009724390.1

LPPAYT

>2S.1.p2.pp3.4-YP_009724390.1

PPAYTN

>2S.1.p2.pp3-Omicron21L-T19I-delL24-delP25-delP26-A27S

TQSYTN

>2S.1.p2.pp4.1-YP_009724390.1

QLPPAY

>2S.1.p2.pp4.2-YP_009724390.1

LPPAYT

>2S.1.p2.pp4.3-YP_009724390.1

PPAYTN

>2S.1.p2.pp4.4-YP_009724390.1

PAYTNS

>2S.1.p2.pp4-Omicron21L-T19I-delL24-delP25-delP26-A27S

QSYTNS

>2S.1.p2.pp5.1-YP_009724390.1

LPPAYT

>2S.1.p2.pp5.2-YP_009724390.1

PPAYTN

>2S.1.p2.pp5.3-YP_009724390.1

PAYTNS

>2S.1.p2.pp5.4-YP_009724390.1

AYTNSF

>2S.1.p2.pp5-Omicron21L-T19I-delL24-delP25-delP26-A27S

SYTNSF

>S.4.pp1-YP_009724390.1

NDPFLG

>S.4.2.pp1-Omicron21L-G142D

NDPFLD

>S.4.pp2.1-YP_009724390.1

DPFLGV

>S.4.2.pp2.1-Omicron21L-G142D

DPFLDV

>S.4.pp3.1-YP_009724390.1

PFLGVY

>S.4.2.pp3.1-Omicron21L-G142D

PFLDVY

>S.4.pp4.1-YP_009724390.1

FLGVYY

>S.4.2.pp4.1-Omicron21L-G142D

FLDVYY

>S.4.pp5.1-YP_009724390.1

LGVYYH

>S.4.2.pp5-Omicron21L-G142D

LDVYYH

>2S.4.pp1-YP_009724390.1

DPFLGV

>2S.4.2.pp1-Omicron21L-G142D

DPFLDV

>2S.4.pp2.1-YP_009724390.1

PFLGVY

>2S.4.2.pp2.1-Omicron21L-G142D

PFLDVY

>2S.4.pp3.1-YP_009724390.1

FLGVYY

>2S.4.2.pp3.1-Omicron21L-G142D

FLDVYY

>2S.4.pp4.1-YP_009724390.1

LGVYYH

>2S.4.2.pp4.1-Omicron21L-G142D

LDVYYH

>2S.4.pp5.1-YP_009724390.1

GVYYHK

>2S.4.2.pp5-Omicron21L-G142D

DVYYHK

>S.5.pp1-YP_009724390.1

TPINLV

>S.5.2.pp1-Omicron21L-V213G

TPINLG

>S.5.pp2-YP_009724390.1

PINLVR

>S.5.2.pp2-Omicron21L-V213G

PINLGR

>S.5.pp3-YP_009724390.1

INLVRD

>S.5.2.pp3-Omicron21L-V213G

INLGRD

>S.5.pp4-YP_009724390.1

NLVRDL

>S.5.2.pp4-Omicron21L-V213G

NLGRDL

>S.5.pp5-YP_009724390.1

LVRDLP

>S.5.2.pp5-Omicron21L-V213G

LGRDLP

>2S.5.pp1-YP_009724390.1

PINLVR

>2S.5.2.pp1-Omicron21L-V213G

PINLGR

>2S.5.pp2-YP_009724390.1

INLVRD

>2S.5.2.pp2-Omicron21L-V213G

INLGRD

>2S.5.pp3-YP_009724390.1

NLVRDL

>2S.5.2.pp3-Omicron21L-V213G

NLGRDL

>2S.5.pp4-YP_009724390.1

LVRDLP

>2S.5.2.pp4-Omicron21L-V213G

LGRDLP

>2S.5.pp5-YP_009724390.1

VRDLPQ

>2S.5.2.pp5-Omicron21L-V213G

GRDLPQ

>S.7.pp1-YP_009724390.1

SVLYNS

>S.7.2.pp1-Omicron21L-S371F-S373P-S375F-T376A

SVLYNF

>S.7.pp2-YP_009724390.1

VLYNSA

>S.7.2.pp2-Omicron21L-S371F-S373P-S375F-T376A

VLYNFA

>S.7.pp3-YP_009724390.1

LYNSAS

>S.7.2.pp3-Omicron21L-S371F-S373P-S375F-T376A

LYNFAP

>S.7.pp4-YP_009724390.1

YNSASF

>S.7.2.pp4-Omicron21L-S371F-S373P-S375F-T376A

YNFAPF

>S.7.pp5-YP_009724390.1

NSASFS

>S.7.2.pp5-Omicron21L-S371F-S373P-S375F-T376A

NFAPFF

>S.7.pp6-YP_009724390.1

SASFST

>S.7.2.pp6-Omicron21L-S371F-S373P-S375F-T376A

FAPFFA

>S.7.pp7-YP_009724390.1

ASFSTF

>S.7.2.pp7-Omicron21L-S371F-S373P-S375F-T376A

APFFAF

>S.7.pp8-YP_009724390.1

SFSTFK

>S.7.2.pp8-Omicron21L-S371F-S373P-S375F-T376A

PFFAFK

>S.7.pp9-YP_009724390.1

FSTFKC

>S.7.2.pp9-Omicron21L-S371F-S373P-S375F-T376A

FFAFKC

>S.7.pp10-YP_009724390.1

STFKCY

>S.7.2.pp10-Omicron21L-S371F-S373P-S375F-T376A

FAFKCY

>2S.7.pp1-YP_009724390.1

VLYNSA

>2S.7.2.pp1-Omicron21L-S371F-S373P-S375F-T376A

VLYNFA

>2S.7.pp2-YP_009724390.1

LYNSAS

>2S.7.2.pp2-Omicron21L-S371F-S373P-S375F-T376A

LYNFAP

>2S.7.pp3-YP_009724390.1

YNSASF

>2S.7.2.pp3-Omicron21L-S371F-S373P-S375F-T376A

YNFAPF

>2S.7.pp4-YP_009724390.1

NSASFS

>2S.7.2.pp4-Omicron21L-S371F-S373P-S375F-T376A

NFAPFF

>2S.7.pp5-YP_009724390.1

SASFST

>2S.7.2.pp5-Omicron21L-S371F-S373P-S375F-T376A

FAPFFA

>2S.7.pp6-YP_009724390.1

ASFSTF

>2S.7.2.pp6-Omicron21L-S371F-S373P-S375F-T376A

APFFAF

>2S.7.pp7-YP_009724390.1

SFSTFK

>2S.7.2.pp7-Omicron21L-S371F-S373P-S375F-T376A

PFFAFK

>2S.7.pp8-YP_009724390.1

FSTFKC

>2S.7.2.pp8-Omicron21L-S371F-S373P-S375F-T376A

FFAFKC

>2S.7.pp9-YP_009724390.1

STFKCY

>2S.7.2.pp9-Omicron21L-S371F-S373P-S375F-T376A

FAFKCY

>2S.7.pp10-YP_009724390.1

TFKCYG

>2S.7.2.pp10-Omicron21L-S371F-S373P-S375F-T376A

AFKCYG

>S.8.pp1-YP_009724390.1

FVIRGD

>S.8.pp1-Omicron21L-D405N-R408S

FVIRGN

>S.8.pp2-YP_009724390.1

VIRGDE

>S.8.pp2-Omicron21L-D405N-R408S

VIRGNE

>S.8.pp3-YP_009724390.1

IRGDEV

>S.8.pp3-Omicron21L-D405N-R408S

IRGNEV

>S.8.pp4-YP_009724390.1

RGDEVR

>S.8.pp4-Omicron21L-D405N-R408S

RGNEVS

>S.8.pp5-YP_009724390.1

GDEVRQ

>S.8.pp5-Omicron21L-D405N-R408S

GNEVSQ

>S.8.pp6-YP_009724390.1

DEVRQI

>S.8.pp6-Omicron21L-D405N-R408S

NEVSQI

>S.8.pp7-YP_009724390.1

EVRQIA

>S.8.pp7-Omicron21L-D405N-R408S

EVSQIA

>S.8.pp8-YP_009724390.1

VRQIAP

>S.8.pp8-Omicron21L-D405N-R408S

VSQIAP

>2S.8.pp1-YP_009724390.1

VIRGDE

>2S.8.pp1-Omicron21L-D405N-R408S

VIRGNE

>2S.8.pp2-YP_009724390.1

IRGDEV

>2S.8.pp2-Omicron21L-D405N-R408S

IRGNEV

>2S.8.pp3-YP_009724390.1

RGDEVR

>2S.8.pp3-Omicron21L-D405N-R408S

RGNEVS

>2S.8.pp4-YP_009724390.1

GDEVRQ

>2S.8.pp4-Omicron21L-D405N-R408S

GNEVSQ

>2S.8.pp5-YP_009724390.1

DEVRQI

>2S.8.pp5-Omicron21L-D405N-R408S

NEVSQI

>2S.8.pp6-YP_009724390.1

EVRQIA

>2S.8.pp6-Omicron21L-D405N-R408S

EVSQIA

>2S.8.pp7-YP_009724390.1

VRQIAP

>2S.8.pp7-Omicron21L-D405N-R408S

VSQIAP

>2S.8.pp8-YP_009724390.1

RQIAPG

>2S.8.pp8-Omicron21L-D405N-R408S

SQIAPG

>S.12.pp4-YP_009724390.1

PLQSYG

>S.12.1.pp4-Omicron21K-Q493R-G496S-Q498R-N501Y-Y505H-21L-Q493R-Q498R-N501Y-Y505H

PLRSYG

>S.12.pp5-YP_009724390.1

LQSYGF

>S.12.1.pp5-Omicron21K-Q493R-G496S-Q498R-N501Y-Y505H-21L-Q493R-Q498R-N501Y-Y505H

LRSYGF

>S.12.pp6-YP_009724390.1

QSYGFQ

>S.12.1.pp6-Omicron21K-Q493R-G496S-Q498R-N501Y-Y505H

RSYGFR

>S.12.pp7-YP_009724390.1

SYGFQP

>S.12.1.pp7-Omicron21K-Q493R-G496S-Q498R-N501Y-Y505H

SYGFRP

>S.12.pp8-YP_009724390.1

YGFQPT

>S.12.1.pp8-Omicron21K-Q493R-G496S-Q498R-N501Y-Y505H

YGFRPT

>S.12.pp9-YP_009724390.1

GFQPTN

>S.12.1.pp9-Omicron21K-Q493R-G496S-Q498R-N501Y-Y505H

GFRPTY

>2S.12.pp3-YP_009724390.1

PLQSYG

>2S.12.1.pp3-Omicron21K-Q493R-G496S-Q498R-N501Y-Y505H-21L-Q493R-Q498R-N501Y-Y505H

PLRSYG

>2S.12.pp4-YP_009724390.1

LQSYGF

>2S.12.1.pp4-Omicron21K-Q493R-G496S-Q498R-N501Y-Y505H-21L-Q493R-Q498R-N501Y-Y505H

LRSYGF

>2S.12.pp5-YP_009724390.1

QSYGFQ

>2S.12.1.pp5-Omicron21K-Q493R-G496S-Q498R-N501Y-Y505H-21L-Q493R-Q498R-N501Y-Y505H

RSYGFR

>2S.12.pp6-YP_009724390.1

SYGFQP

>2S.12.1.pp6-Omicron21K-Q493R-G496S-Q498R-N501Y-Y505H

SYGFRP

>2S.12.pp7-YP_009724390.1

YGFQPT

>2S.12.1.pp7-Omicron21K-Q493R-G496S-Q498R-N501Y-Y505H

YGFRPT

>2S.12.pp8-YP_009724390.1

GFQPTN

>2S.12.1.pp8-Omicron21K-Q493R-G496S-Q498R-N501Y-Y505H

GFRPTY

>N.4.pp1-YP_009724397.2

QQSMSS

>N.4.pp1-Omicron21L-S413R

QQSMSR

>N.4.pp2-YP_009724397.2

QSMSSA

>N.4.pp2-Omicron21L-S413R

QSMSRA

>N.4.pp3-YP_009724397.2

SMSSAD

>N.4.pp3-Omicron21L-S413R

SMSRAD

>N.4.pp4-YP_009724397.2

MSSADS

>N.4.pp4-Omicron21L-S413R

MSRADS

>N.4.pp5-YP_009724397.2

SSADST

>N.4.pp5-Omicron21L-S413R

SRADST

>2N.4.pp1-YP_009724397.2

QSMSSA

>2N.4.pp1-Omicron21L-S413R

QSMSRA

>2N.4.pp2-YP_009724397.2

SMSSAD

>2N.4.pp2-Omicron21L-S413R

SMSRAD

>2N.4.pp3-YP_009724397.2

MSSADS

>2N.4.pp3-Omicron21L-S413R

MSRADS

>2N.4.pp4-YP_009724397.2

SSADST

>2N.4.pp4-Omicron21L-S413R

SRADST

>2N.4.pp5-YP_009724397.2

SADSTQ

>2N.4.pp5-Omicron21L-S413R

RADSTQ

>Orf1ab.1.pp1-YP_009724389.1

GAGGHS

>Orf1ab.1.pp1-Omicron21L-S135R

GAGGHR

>Orf1ab.1.pp2-YP_009724389.1

AGGHSY

>Orf1ab.1.pp2-Omicron21L-S135R

AGGHRY

>Orf1ab.1.pp3-YP_009724389.1

GGHSYG

>Orf1ab.1.pp3-Omicron21L-S135R

GGHRYG

>Orf1ab.1.pp4-YP_009724389.1

GHSYGA

>Orf1ab.1.pp4-Omicron21L-S135R

GHRYGA

>Orf1ab.1.pp5-YP_009724389.1

HSYGAD

>Orf1ab.1.pp5-Omicron21L-S135R

HRYGAD

>2Orf1ab.1.pp1-YP_009724389.1

AGGHSY

>2Orf1ab.1.pp1-Omicron21L-S135R

AGGHRY

>2Orf1ab.1.pp2-YP_009724389.1

GGHSYG

>2Orf1ab.1.pp2-Omicron21L-S135R

GGHRYG

>2Orf1ab.1.pp3-YP_009724389.1

GHSYGA

>2Orf1ab.1.pp3-Omicron21L-S135R

GHRYGA

>2Orf1ab.1.pp4-YP_009724389.1

HSYGAD

>2Orf1ab.1.pp4-Omicron21L-S135R

HRYGAD

>2Orf1ab.1.pp5-YP_009724389.1

SYGADL

>2Orf1ab.1.pp5-Omicron21L-S135R

RYGADL

>Orf1ab.2.pp1-YP_009724389.1

KSVNIT

>Orf1ab.2.pp1-Omicron21L-T842I

KSVNII

>Orf1ab.2.pp2-YP_009724389.1

SVNITF

>Orf1ab.2.pp2-Omicron21L-T842I

SVNIIF

>Orf1ab.2.pp3-YP_009724389.1

VNITFE

>Orf1ab.2.pp3-Omicron21L-T842I

VNIIFE

>Orf1ab.2.pp4-YP_009724389.1

NITFEL

>Orf1ab.2.pp4-Omicron21L-T842I

NIIFEL

>Orf1ab.2.pp5-YP_009724389.1

ITFELD

>Orf1ab.2.pp5-Omicron21L-T842I

IIFELD

>2Orf1ab.2.pp1-YP_009724389.1

SVNITF

>2Orf1ab.2.pp1-Omicron21L-T842I

SVNIIF

>2Orf1ab.2.pp2-YP_009724389.1

VNITFE

>2Orf1ab.2.pp2-Omicron21L-T842I

VNIIFE

>2Orf1ab.2.pp3-YP_009724389.1

NITFEL

>2Orf1ab.2.pp3-Omicron21L-T842I

NIIFEL

>2Orf1ab.2.pp4-YP_009724389.1

ITFELD

>2Orf1ab.2.pp4-Omicron21L-T842I

IIFELD

>2Orf1ab.2.pp5-YP_009724389.1

TFELDE

>2Orf1ab.2.pp5-Omicron21L-T842I

IFELDE

>Orf1ab.4.pp1-YP_009724389.1

PTKKAG

>Orf1ab.4.pp1-Omicron21L-G1307S

PTKKAS

>Orf1ab.4.pp2-YP_009724389.1

TKKAGG

>Orf1ab.4.pp2-Omicron21L-G1307S

TKKASG

>Orf1ab.4.pp3-YP_009724389.1

KKAGGT

>Orf1ab.4.pp3-Omicron21L-G1307S

KKASGT

>Orf1ab.4.pp4-YP_009724389.1

KAGGTT

>Orf1ab.4.pp4-Omicron21L-G1307S

KASGTT

>Orf1ab.4.pp5-YP_009724389.1

AGGTTE

>Orf1ab.4.pp5-Omicron21L-G1307S

ASGTTE

>2Orf1ab.4.pp1-YP_009724389.1

TKKAGG

>2Orf1ab.4.pp1-Omicron21L-G1307S

TKKASG

>2Orf1ab.4.pp2-YP_009724389.1

KKAGGT

>2Orf1ab.4.pp2-Omicron21L-G1307S

KKASGT

>2Orf1ab.4.pp3-YP_009724389.1

KAGGTT

>2Orf1ab.4.pp3-Omicron21L-G1307S

KASGTT

>2Orf1ab.4.pp4-YP_009724389.1

AGGTTE

>2Orf1ab.4.pp4-Omicron21L-G1307S

ASGTTE

>2Orf1ab.4.pp5-YP_009724389.1

GGTTEM

>2Orf1ab.4.pp5-Omicron21L-G1307S

SGTTEM

>Orf1ab.7.pp1-YP_009724389.1

DAVNLL

>Orf1ab.7.pp1-Omicron21L-L3027F

DAVNLF

>Orf1ab.7.pp2-YP_009724389.1

AVNLLT

>Orf1ab.7.pp2-Omicron21L-L3027F

AVNLFT

>Orf1ab.7.pp3-YP_009724389.1

VNLLTN

>Orf1ab.7.pp3-Omicron21L-L3027F

VNLFTN

>Orf1ab.7.pp4-YP_009724389.1

NLLTNM

>Orf1ab.7.pp4-Omicron21L-L3027F

NLFTNM

>Orf1ab.7.pp5-YP_009724389.1

LLTNMF

>Orf1ab.7.pp5-Omicron21L-L3027F

LFTNMF

>2Orf1ab.7.pp1-YP_009724389.1

AVNLLT

>2Orf1ab.7.pp1-Omicron21L-L3027F

AVNLFT

>2Orf1ab.7.pp2-YP_009724389.1

VNLLTN

>2Orf1ab.7.pp2-Omicron21L-L3027F

VNLFTN

>2Orf1ab.7.pp3-YP_009724389.1

NLLTNM

>2Orf1ab.7.pp3-Omicron21L-L3027F

NLFTNM

>2Orf1ab.7.pp4-YP_009724389.1

LLTNMF

>2Orf1ab.7.pp4-Omicron21L-L3027F

LFTNMF

>2Orf1ab.7.pp5-YP_009724389.1

LTNMFT

>2Orf1ab.7.pp5-Omicron21L-L3027F

FTNMFT

>Orf1ab.8.pp1-YP_009724389.1

FLMSFT

>Orf1ab.8.pp1-Omicron21L-T3090I

FLMSFI

>Orf1ab.8.pp2-YP_009724389.1

LMSFTV

>Orf1ab.8.pp2-Omicron21L-T3090I

LMSFIV

>Orf1ab.8.pp3-YP_009724389.1

MSFTVL

>Orf1ab.8.pp3-Omicron21L-T3090I

MSFIVL

>Orf1ab.8.pp4-YP_009724389.1

SFTVLC

>Orf1ab.8.pp4-Omicron21L-T3090I

SFIVLC

>Orf1ab.8.pp5-YP_009724389.1

FTVLCL

>Orf1ab.8.pp5-Omicron21L-T3090I

FIVLCL

>2Orf1ab.8.pp1-YP_009724389.1

LMSFTV

>2Orf1ab.8.pp1-Omicron21L-T3090I

LMSFIV

>2Orf1ab.8.pp2-YP_009724389.1

MSFTVL

>2Orf1ab.8.pp2-Omicron21L-T3090I

MSFIVL

>2Orf1ab.8.pp3-YP_009724389.1

SFTVLC

>2Orf1ab.8.pp3-Omicron21L-T3090I

SFIVLC

>2Orf1ab.8.pp4-YP_009724389.1

FTVLCL

>2Orf1ab.8.pp4-Omicron21L-T3090I

FIVLCL

>2Orf1ab.8.pp5-YP_009724389.1

TVLCLT

>2Orf1ab.8.pp5-Omicron21L-T3090I

IVLCLT

>Orf1ab.9.pp1-YP_009724389.1

DVLLPL

>Orf1ab.9.pp1-Omicron21L-L3201F

DVLLPF

>Orf1ab.9.pp2-YP_009724389.1

VLLPLT

>Orf1ab.9.pp2-Omicron21L-L3201F

VLLPFT

>Orf1ab.9.pp3-YP_009724389.1

LLPLTQ

>Orf1ab.9.pp3-Omicron21L-L3201F

LLPFTQ

>Orf1ab.9.pp4-YP_009724389.1

LPLTQY

>Orf1ab.9.pp4-Omicron21L-L3201F

LPFTQY

>Orf1ab.9.pp5-YP_009724389.1

PLTQYN

>Orf1ab.9.pp5-Omicron21L-L3201F

PFTQYN

>2Orf1ab.9.pp1-YP_009724389.1

VLLPLT

>2Orf1ab.9.pp1-Omicron21L-L3201F

VLLPFT

>2Orf1ab.9.pp2-YP_009724389.1

LLPLTQ

>2Orf1ab.9.pp2-Omicron21L-L3201F

LLPFTQ

>2Orf1ab.9.pp3-YP_009724389.1

LPLTQY

>2Orf1ab.9.pp3-Omicron21L-L3201F

LPFTQY

>2Orf1ab.9.pp4-YP_009724389.1

PLTQYN

>2Orf1ab.9.pp4-Omicron21L-L3201F

PFTQYN

>2Orf1ab.9.pp5-YP_009724389.1

LTQYNR

>2Orf1ab.9.pp5-Omicron21L-L3201F

FTQYNR

>Orf1ab.12.pp1.-YP_009724389.1

VDTSLS

>Orf1ab.12.pp1.-YP_009724389.1

DTSLSG

>Orf1ab.12.pp1.-YP_009724389.1

TSLSGF

>Orf1ab.12.pp1.-YP_009724389.1

SLSGFK

>Orf1ab.12.2.pp1-Omicron21L-delS3675-delG3676-delF3677

VDTSLK

>Orf1ab.12.pp2.1-YP_009724389.1

DTSLSG

>Orf1ab.12.pp2.2-YP_009724389.1

TSLSGF

>Orf1ab.12.pp2.3-YP_009724389.1

SLSGFK

>Orf1ab.12.pp2.4-YP_009724389.1

LSGFKL

>Orf1ab.12.2.pp2-Omicron21L-delS3675-delG3676-delF3677

DTSLKL

>Orf1ab.12.pp3.1-YP_009724389.1

TSLSGF

>Orf1ab.12.pp3.2-YP_009724389.1

SLSGFK

>Orf1ab.12.pp3.3-YP_009724389.1

LSGFKL

>Orf1ab.12.pp3.4-YP_009724389.1

SGFKLK

>Orf1ab.12.2.pp3-Omicron21L-delS3675-delG3676-delF3677

TSLKLK

>Orf1ab.12.pp4.1-YP_009724389.1

SLSGFK

>Orf1ab.12.pp4.2-YP_009724389.1

LSGFKL

>Orf1ab.12.pp4.3-YP_009724389.1

SGFKLK

>Orf1ab.12.pp4.4-YP_009724389.1

GFKLKD

>Orf1ab.12.2.pp4-Omicron21L-delS3675-delG3676-delF3677

SLKLKD

>2Orf1ab.12.pp1.-YP_009724389.1

DTSLSG

>2Orf1ab.12.pp1.-YP_009724389.1

TSLSGF

>2Orf1ab.12.pp1.-YP_009724389.1

SLSGFK

>2Orf1ab.12.pp1.-YP_009724389.1

LSGFKL

>2Orf1ab.12.2.pp1-Omicron21L-delS3675-delG3676-delF3677

DTSLKL

>2Orf1ab.12.pp2.1-YP_009724389.1

TSLSGF

>2Orf1ab.12.pp2.2-YP_009724389.1

SLSGFK

>2Orf1ab.12.pp2.3-YP_009724389.1

LSGFKL

>2Orf1ab.12.pp2.4-YP_009724389.1

SGFKLK

>2Orf1ab.12.2.pp2-Omicron21L-delS3675-delG3676-delF3677

TSLKLK

>2Orf1ab.12.pp3.1-YP_009724389.1

SLSGFK

>2Orf1ab.12.pp3.2-YP_009724389.1

LSGFKL

>2Orf1ab.12.pp3.3-YP_009724389.1

SGFKLK

>2Orf1ab.12.pp3.4-YP_009724389.1

GFKLKD

>2Orf1ab.12.2.pp3-Omicron21L-delS3675-delG3676-delF3677

SLKLKD

>2Orf1ab.12.pp4.1-YP_009724389.1

LSGFKL

>2Orf1ab.12.pp4.2-YP_009724389.1

SGFKLK

>2Orf1ab.12.pp4.3-YP_009724389.1

GFKLKD

>2Orf1ab.12.pp4.4-YP_009724389.1

FKLKDC

>2Orf1ab.12.2.pp4-Omicron21L-delS3675-delG3676-delF3677

LKLKDC

>Orf1ab.2.pp1-YP_009724389.1

VNARLR

>Orf1ab.2.pp1-Omicron21L-R1315C

VNARLC

>Orf1ab.2.pp2-YP_009724389.1

NARLRA

>Orf1ab.2.pp2-Omicron21L-R1315C

NARLCA

>Orf1ab.2.pp3-YP_009724389.1

ARLRAK

>Orf1ab.2.pp3-Omicron21L-R1315C

ARLCAK

>Orf1ab.2.pp4-YP_009724389.1

RLRAKH

>Orf1ab.2.pp4-Omicron21L-R1315C

RLCAKH

>Orf1ab.2.pp5-YP_009724389.1

LRAKHY

>Orf1ab.2.pp5-Omicron21L-R1315C

LCAKHY

>2Orf1ab.2.pp1-YP_009724389.1

NARLRA

>2Orf1ab.2.pp1-Omicron21L-R1315C

NARLCA

>2Orf1ab.2.pp2-YP_009724389.1

ARLRAK

>2Orf1ab.2.pp2-Omicron21L-R1315C

ARLCAK

>2Orf1ab.2.pp3-YP_009724389.1

RLRAKH

>2Orf1ab.2.pp3-Omicron21L-R1315C

RLCAKH

>2Orf1ab.2.pp4-YP_009724389.1

LRAKHY

>2Orf1ab.2.pp4-Omicron21L-R1315C

LCAKHY

>2Orf1ab.2.pp5-YP_009724389.1

RAKHYV

>2Orf1ab.2.pp5-Omicron21L-R1315C

CAKHYV

>Orf1ab.4.pp1-YP_009724389.1

IAKKPT

>Orf1ab.4.pp1-Omicron21L-T2163I

IAKKPI

>Orf1ab.4.pp2-YP_009724389.1

AKKPTE

>Orf1ab.4.pp2-Omicron21L-T2163I

AKKPIE

>Orf1ab.4.pp3-YP_009724389.1

KKPTET

>Orf1ab.4.pp3-Omicron21L-T2163I

KKPIET

>Orf1ab.4.pp4-YP_009724389.1

KPTETI

>Orf1ab.4.pp4-Omicron21L-T2163I

KPIETI

>Orf1ab.4.pp5-YP_009724389.1

PTETIC

>Orf1ab.4.pp5-Omicron21L-T2163I

PIETIC

>2Orf1ab.4.pp1-YP_009724389.1

AKKPTE

>2Orf1ab.4.pp1-Omicron21L-T2163I

AKKPIE

>2Orf1ab.4.pp2-YP_009724389.1

KKPTET

>2Orf1ab.4.pp2-Omicron21L-T2163I

KKPIET

>2Orf1ab.4.pp3-YP_009724389.1

KPTETI

>2Orf1ab.4.pp3-Omicron21L-T2163I

KPIETI

>2Orf1ab.4.pp4-YP_009724389.1

PTETIC

>2Orf1ab.4.pp4-Omicron21L-T2163I

PIETIC

>2Orf1ab.4.pp5-YP_009724389.1

TETICA

>2Orf1ab.4.pp5-Omicron21L-T2163I

IETICA

>Orf3a.1.pp1-YP_009724391.1

QLSTDT

>Orf3a.1.pp1-Omicron21L-T223I

QLSTDI

>Orf3a.1.pp2-YP_009724391.1

LSTDTG

>Orf3a.1.pp2-Omicron21L-T223I

LSTDIG

>Orf3a.1.pp3-YP_009724391.1

STDTGV

>Orf3a.1.pp3-Omicron21L-T223I

STDIGV

>Orf3a.1.pp4-YP_009724391.1

TDTGVE

>Orf3a.1.pp4-Omicron21L-T223I

TDIGVE

>Orf3a.1.pp5-YP_009724391.1

DTGVEH

>Orf3a.1.pp5-Omicron21L-T223I

DIGVEH

>2Orf3a.1.pp1-YP_009724391.1

LSTDTG

>2Orf3a.1.pp1-Omicron21L-T223I

LSTDIG

>2Orf3a.1.pp2-YP_009724391.1

STDTGV

>2Orf3a.1.pp2-Omicron21L-T223I

STDIGV

>2Orf3a.1.pp3-YP_009724391.1

TDTGVE

>2Orf3a.1.pp3-Omicron21L-T223I

TDIGVE

>2Orf3a.1.pp4-YP_009724391.1

DTGVEH

>2Orf3a.1.pp4-Omicron21L-T223I

DIGVEH

>2Orf3a.1.pp5-YP_009724391.1

TGVEHV

>2Orf3a.1.pp5-Omicron21L-T223I

IGVEHV

>Orf6.1.pp1-YP_009724394.1

QPMEID

>Orf6.1.pp1-Omicron21L-D61L

QPMEIL
