# Supplementary figures and images for: Shared 6mer Peptides of Human and Omicron (21K and 21L) at SARS-CoV-2 Mutation Sites
Source: Antibodies (Basel). 2022 Oct 25;11(4):68. doi: 10.3390/antib11040068 (PMC9680445; doi:10.3390/antib11040068)

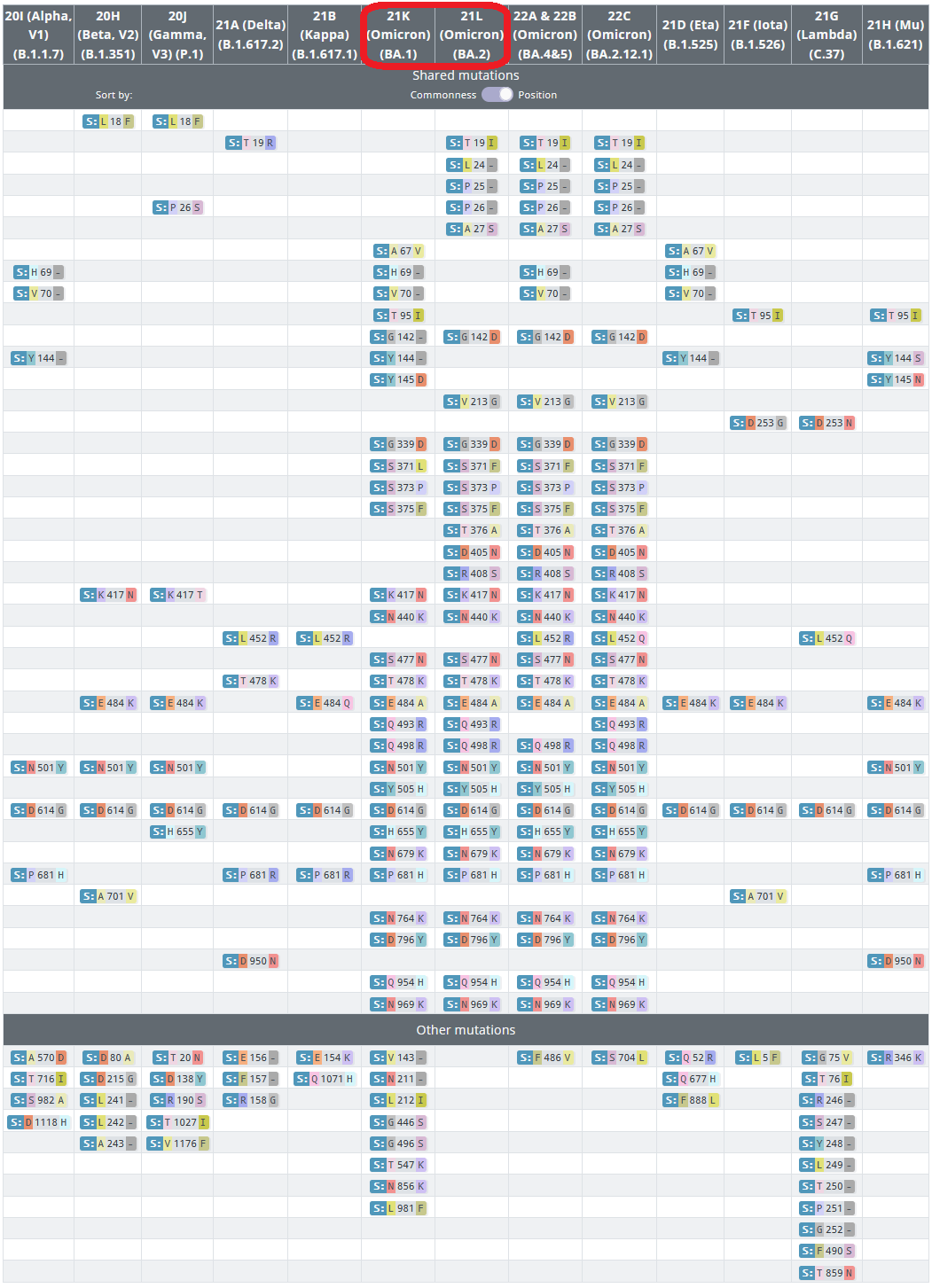

Supplement: Supplementary file 1 [file antibodies-11-00068-s001.zip › FigureS1_mutations of interest from covariants.org.png]
